# Design, Synthesis, and Biological Application of Novel Photoaffinity Probes of Dihydropyridine Derivatives, BAY R3401

**DOI:** 10.3390/molecules24132394

**Published:** 2019-06-28

**Authors:** Liying Zhang, Zhiwei Yan, Youde Wang, Chengjun Song, Guangxin Miao

**Affiliations:** 1 Key Laboratory of Traditional Chinese Medicine Research and Development of Hebei Province, Institute of Traditional Chinese Medicine, Chengde Medical University, Chengde 067000, China; 2 Department of Human Anatomy, Chengde Medical University, Chengde 067000, China

**Keywords:** BAY R3401, molecular mechanism, type 2 diabetes, photoaffinity probe, glycogenolysis, photoaffinity labeling, target proteins

## Abstract

To explore the molecular mechanisms of BAY R3401, four types of novel photoaffinity probes bearing different secondary tags were synthesized. Their potency for glycogenolysis was evaluated in primary human liver HL-7702 cells and HepG2 cells. Probe 2d showed the best activity in primary human liver HL-7702 cells and HepG2 cells, with IC_50_ values of 4.45 μM and 28.49 μM, respectively. Likewise, probe 5d showed IC_50_ values of 6.46 μM in primary human liver HL-7702 cells and 15.29 μM in HepG2 cells, respectively. Photoaffinity labeling experiments were also performed and protein bands larger than 170 kDa were specifically tagged by probe 2d. The results suggest that the synthesized probe 2d might be a very promising tool for the isolation of the target proteins of BAY R3401.

Academic Editor: Run Zhang, D. Amilan Jose, Hang Thu Ta and Mingqian Tan

## 1. Introduction

BAY R3401 is an orally bioavailable hypoglycemic agent for the treatment of type 2 diabetes, as reported by the Bayer Pharmaceutical Company [1]. This agent allows irreversible, nonselective suppression of hepatic glycogenolysis by inhibiting glycogen phosphorylase, which is the rate controlling enzyme of the glycogenolytic pathway [2]. The active metabolite, W1807, contributes significantly to its activity [3]. Nonetheless, much to the researchers’ surprise, BAY R3401 inactivated glycogen phosphorylase by 63%, but glucose output dropped by 83% in the perfused liver [4]. It is difficult to explain the effects based only on the reported mechanism. Therefore, the exact mode of action of BAY R3401 has not been established.

Photoaffinity labeling is one of the major methods to directly capture small-molecule binding proteins [5]. The conventional approach, however, usually relies on the synthesis of photoaffinity probes and the identification of photolabeled fragments in proteins [6]. In general, a typical photoaffinity probe contains three functional groups. A bioactive scaffold ferries the probe to the enzyme active site, a photoreactive group generates a covalent and irreversible linkage between the probe and its target macromolecule after UV irradiation, and a tag (such as biotin or fluorophore) detects and/or visualizes the modified target enzymes [7].

**Figure 1:**
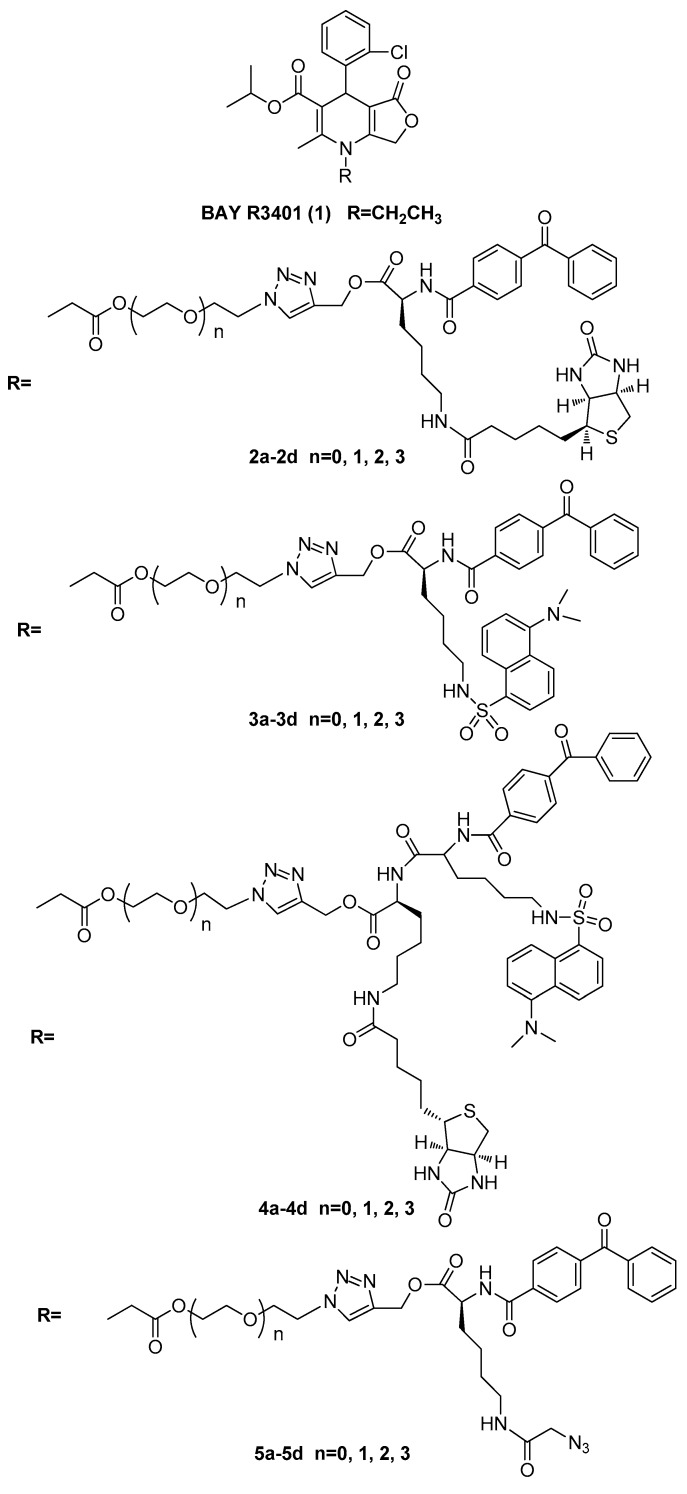
Structures of BAY R3401 (**1**), and synthetic photoaffinity probes possessing biotin (**2a**–**2d**), dansyl (**3a**–**3d**), a dual-functional tag (**4a**–**4d**), or azide (**5a**–**5d**).

Herein, we report the synthesis and biological application of four types of photoaffinity probes based on BAY R3401, which contains both a benzophenone photophore for covalent labeling of target proteins and a secondary handle for the subsequent detection or manipulation of labeled proteins (Figure 1). Probes bearing different secondary tags were exploited, either by direct attachment of a dansyl fluorescent or a biotin tag for detection and enrichment. Moreover, we developed a dual-functional tag containing both a dansyl group and a biotin, which is suitable for both affinity purification and fluorescence applications. In order to avoid the sterically hindrance caused by the large tags, we designed another tag-free probe that employed an azide handle for downstream conjugation to the reporter tag via the click-chemistry reaction after proteome labeling. According to the previous primary structure-activity relationship of the dihydropyridine derivative, modification of the *N1* position of BAY R3401 had little effect on its potency. However, the molecular size of the secondary tags is not very small compared to BAY R3401. Thus, it is not hard to speculate that hindrance might sterically occur due to interfere from the interaction between BAY R3401 and its target proteins when secondary tags are introduced to the *N1* position of BAY R3401 directly. Therefore, appropriate linkages were designed to provide enough space between BAY R3401 and the secondary tags.

## 2. Results and Discussion

### 2.1. Chemistry

**Figure 2:**
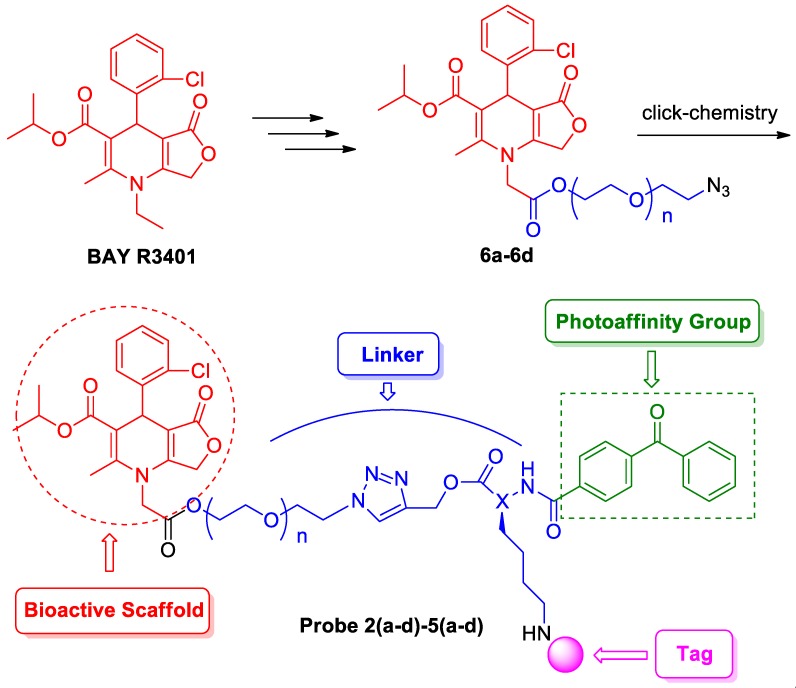
General synthetic strategy for photoaffinity probes by click-chemistry reaction.

The general synthetic strategy employing a click reaction for the described activity probes is outlined in Figure 2. 

**Scheme 1:**
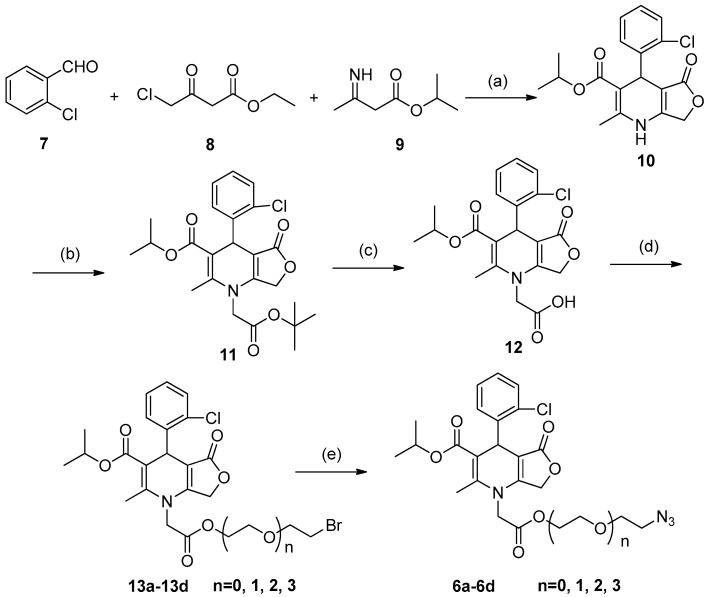
Reagents and conditions: (**a**) isopropyl alcohol, reflux (17%); (**b**) (i) NaH, 0 °C to 80 °C; (ii) tert-butyl 2-chloroacetate, 80 °C (40% for 2 steps); (**c**) trifluoroacetic acid (TFA), CH_2_Cl_2_, 0 °C to r.t. (46%); (**d**) 1,2-dibromoethane or 2,2’-Dibromodiethyl ether or 1,2-Bis(2-bromoethoxy)ethane, K_2_CO_3_, anhydrous CH_3_CN, 0 °C to 80 °C (58–62%); (**e**) NaN_3_, DMF, r.t. (59–90%).

To realize the click chemistry between BAY R3401 and the photoaffinity label moiety, azide functions were first introduced into the position of the ethyl branches based on previous SAR studies (Scheme 1) [8]. Treatment of 2-chlorobenzaldehyde **7** and ethyl 4-chloroacetoacetate **8** with excess isopropyl 3-aminocrotonate **9** in a one-pot reaction at reflux overnight in isopropanol yielded the 1,4-dihydropyridine nucleus **10** with a 17% yield. The alkylation of **10** with tert-butyl chloroacetate produced 1-alkyl-1,4-dihydropyridine derivative **11** with a 40% yield. Deprotection of **11** with trifluoroacetic acid (TFA) produced carboxylic acid **12** (46%). Esterification of **12** with three different linker groups (such as 1,2-dibromoethane, 2,2’-Dibromodiethyl ether, and 1,2-Bis(2-bromoethoxy)ethane) yielded the corresponding bromides, **13a**–**13d** (58–62%), which were converted to azides **6a**–**6d** via a nucleophilic substitution reaction with sodium azide (NaN_3_) with satisfactory yields (59–90%).

**Scheme 2:**
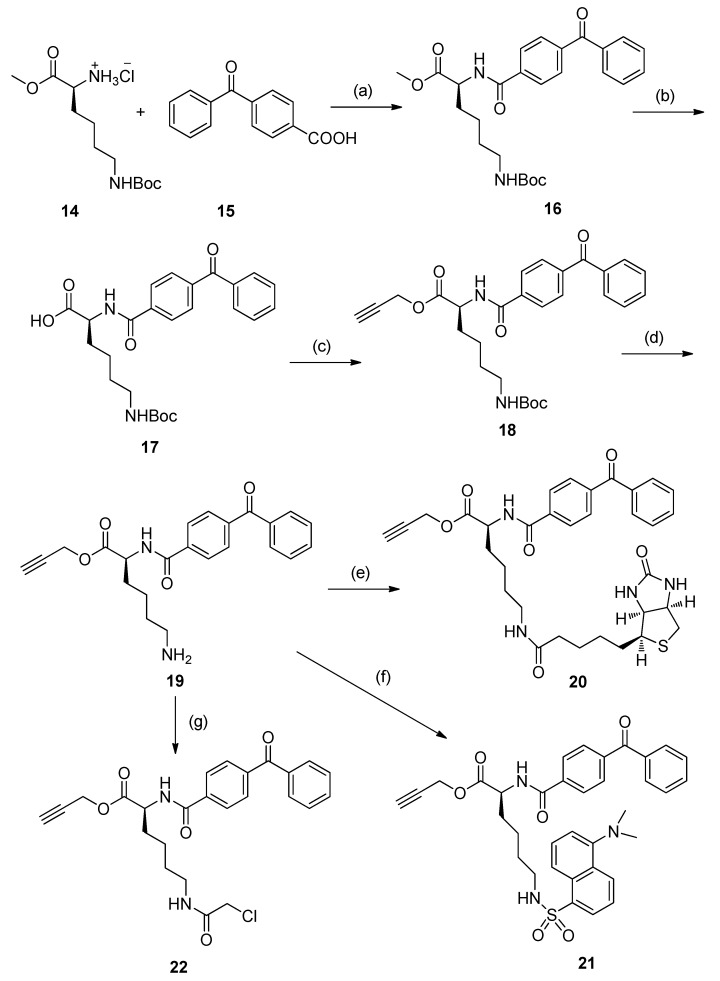
Reagents and conditions: (**a**) EDCI, NMM, DMAP, DMF, r.t. (87%); (**b**) 2 N NaOH, CH_3_OH, 0 °C to r.t.; (**c**) 3-Bromopropyne, K_2_CO_3_, DMF, r.t. (68%); (**d**) TFA, CH_2_Cl_2_, 0 °C to r.t.; (**e**) D-biotin, EDCI, HOBt, DIPEA, DMF (37%); (**f**) dansyl chloride (DNS-Cl), Et_3_N, CH_2_Cl_2_, r.t. (81%); (**g**) ClCH_2_COCl, Et_3_N, CH_2_Cl_2_, 0 °C to r.t. (86%).

The key intermediates, **20**–**22**, were prepared individually via procedures similar to those reported previously, with some modifications (Scheme 2) [9]. Treatment of Lys(Boc)-OMe with 4-benzoylbenzoic acid using EDCI/DMAP afforded compound **16**, bearing a benzophenone photophore. Hydrolysis of **16** with aqueous NaOH yielded carboxylic acid **17**, followed by an esterification with propargyl bromide to produce the alkyne **18**. Subsequent deprotection of **18** using TFA gave amide **19**, then coupling **19** with D-biotin in DMF in the presence of EDCI, HOBt, and DIPEA as condensing agents produced biotin conjugate **20** with a biotin tag. Treatment of amino **19** with dansyl chloride (DNS-Cl) gave the desired fluorescent derivative **21**. Amidation of amino **19** with ClCH_2_COCl was carried out to produce chloroacetyl compound **22** for the next step of azide displacement.

**Scheme 3:**
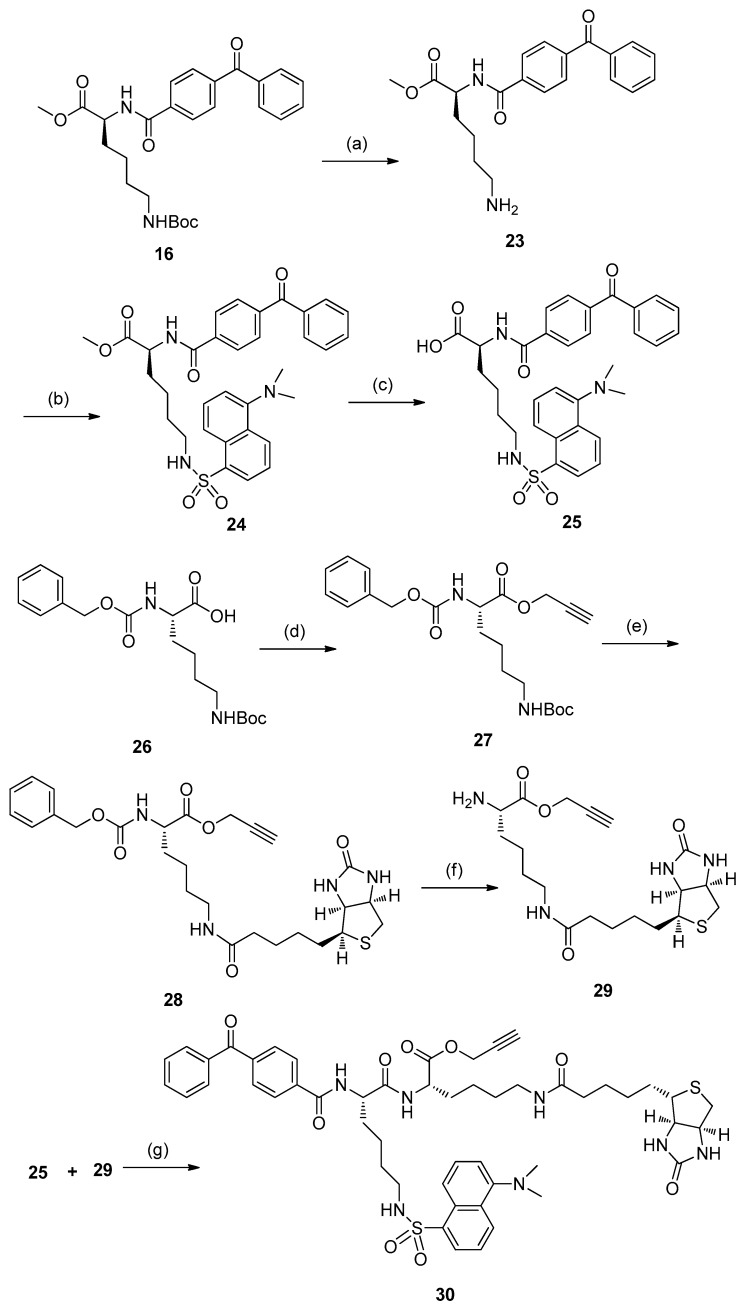
Reagents and conditions: (**a**) TFA, CH_2_Cl_2_, 0 °C to r.t.; (**b**) DNS-Cl, Et_3_N, CH_2_Cl_2_, r.t. (67%); (**c**) 6 N LiOH, THF/H_2_O, 0 °C to r.t. (88%); (**d**) 3-Bromopropyne, K_2_CO_3_, DMF, r.t. (72%); (**e**) (i) TFA, CH_2_Cl_2_, 0 °C to r.t.; (ii) D-biotin, isobutyl chloroformate, Et_3_N, DMF (77%); (**f**) HBr/HOAC, 0 °C to r.t.; (**g**) HATU, DIPEA, DMF, 0 °C to r.t. (43%).

The preparation of a dual-labeled moiety was accomplished as follows (Scheme 3). The synthesis cycle began with deprotection of **16** using TFA, producing amide **23**. Then, amidation with DNS-Cl gave compound **24**. Hydrolysis of **24** with aqueous LiOH afforded carboxylic acid **25** with an 87% yield. Treatment of *N*-Cbz-*N*’-Boc-L-lysine with propargyl bromide gave alkyne **27**. Subsequent deprotection of **27** using TFA, followed by coupling with D-biotin in DMF in the presence of isobutyl chloroformate and Et_3_N as condensing agents, produced biotin conjugate **28**. The *N*-Cbz group was hydrolyzed off in 30% HBr in acetic acid at room temperature to give the amide compound **29**. Reaction of **29** with carboxylic acid derivative **25** in the presence of HATU and DIPEA produced the dual label moiety with a 43% yield.

**Scheme 4:**
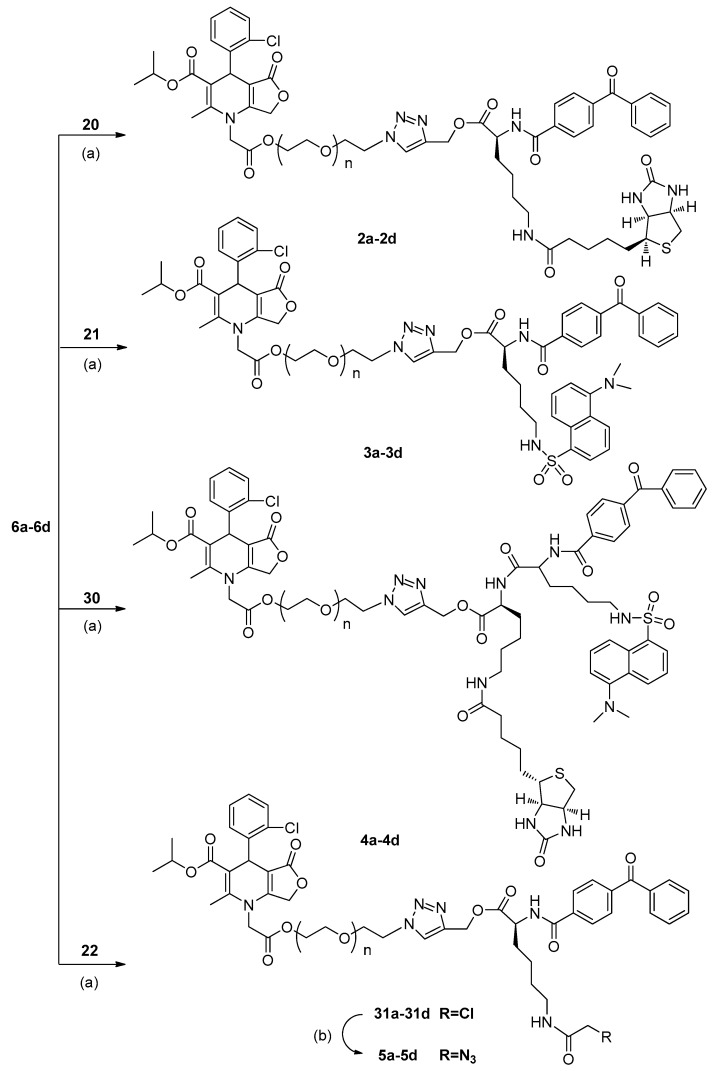
Reagents and conditions: (**a**) CuSO_4_·5H_2_O, sodium ascorbate, CH_2_Cl_2_-H_2_O, r.t.; (**b**) NaN_3_, DMF, r.t.

Click chemistry was handled as shown in Scheme 4. The corresponding azides and alkynes were dissolved in CH_2_Cl_2_-H_2_O, followed by the addition of catalytic sodium ascorbate and CuSO_4_·5H_2_O. Reaction of the above azides **6a**–**6d** with alkynes **20**, **21**, and **30** gave, respectively, probes **2a**–**2d** (21–25%), **3a**–**3d** (13–27%), and **4a**–**4d** (13–27%). In a similar way, the conversion of **6a**–**6d** to the corresponding chloroacetyl compounds, **31a**–**31d**, was followed by a reaction with NaN_3_ to give probes **5a**–**5d**.

**Scheme 5:**
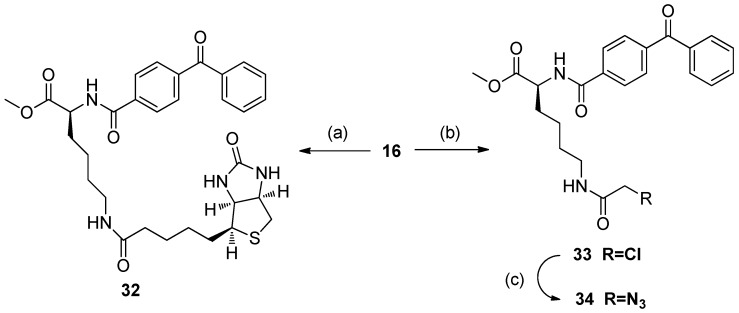
(**a**) i) TFA, CH_2_Cl_2_, 0 °C to r.t.; ii) D-biotin, EDCI, HOBt, DIPEA, DMF (51%); (**b**) ClCH_2_COCl, Et_3_N, CH_2_Cl_2_, 0 °C to r.t. (68%); (**c**) NaN_3_, DMF, r.t. (77%).

The synthesis of control compounds **32** and **34**, which lack the bioactive ligand BAY R3401 and only contain the photoaffinity and tag groups, is shown in Scheme 5. The cycle was started with the deprotection step, as above, followed by amidation with D-biotin and ClCH_2_COCl to give the control compound **32**, and intermediate haloester **33**. Then, treatment of **33** with NaN_3_ produced the control compound **34** with a 77% yield.

**Scheme 6:**
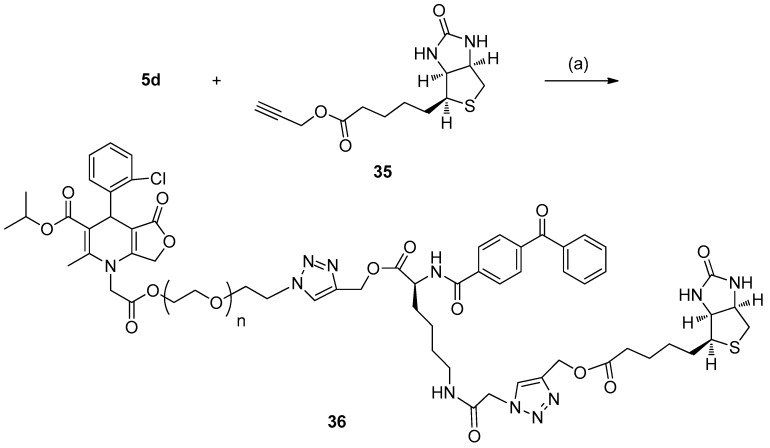
(**a**) CuSO_4_·5H_2_O, sodium ascorbate, CH_2_Cl_2_-H_2_O, r.t. (13%).

To confirm the efficacy of designed probes **5a**–**5d** in combining the tag after protein labeling, the reaction of the tag-free probe **5d** with the alkyne-coupled biotin derivative in a simple model system was also studied (Scheme 6). The biotin derivative **35** was prepared based on a previously reported procedure described in [10]. Treatment of probe **5d** with alkyne-biotin **35** under typical click conditions (CuSO_4_·5H_2_O, with sodium ascorbate as the reducing agent) in CH_2_Cl_2_-H_2_O, the conjugate product **36** could be isolated with a 13% yield as a white solid. These results demonstrated that the azide handle of the tag-free probe can be subsequently conjugated with an alkyne-tag through biocompatible copper-catalyzed azide-alkyne cycloaddition.

### 2.2. Cell Assay and SAR Analysis. 

It is obviously important that the synthesized probes retain potency in bioassays. To evaluate the effects of all probes, the glycogenolysis assays were established in vitro, with primary human liver HL-7702 cells and HepG2 cells, based on the published method [11]. A well-known chloroindole inhibitor of glycogenolysis, CP-91149, was used as a positive control in these experiments.

The IC_50_ values of the tested derivatives are listed in Table 1. Most of the newly synthesized probes maintained moderate inhibitory activity against glucagon-stimulated glycogenolysis in primary human liver HL-7702 cells and HepG2 cells. It is interesting to note that modification of dihydropyridine scaffold with bulky substituents resulted in a great increase in IC_50_ both in primary human liver HL-7702 cells and HepG2 cells (e.g., BAY R3401 vs. **2b**–**2d**, **3b**–**3d**, **5a**, **5c**, **5d**, **6a**). The results are consistent with the SAR analysis of W1807, the active metabolite in cells of BAY R3401, which revealed that the *N1*-substituent may productive van der Waals interactions between the substitutions and its target proteins [8]. With different linkers, SAR analysis in HepG2 cells shows that the distance between the BAY R3401 moiety and secondary tags’ moiety is important—a longer linker led to better inhibitory activity (e.g., **2a** vs. **2d,** **3a** vs. **3d,** and **5a** vs. **5d**). However, data analysis indicated no clear SAR for the distance in primary human liver HL-7702 cells. Within this series of compounds, probe **2d** showed the best activity in primary human liver HL-7702 cells and HepG2 cells, with IC_50_ values of 4.45 μM and 28.49 μM, respectively. Likewise, probe **5d** showed an IC_50_ value of 6.46 μM in primary human liver HL-7702 cells and 15.29 μM in HepG2 cells, respectively. Therefore, probe **2d** and **5d** may be very promising tools for isolation of the target proteins of BAY R3401.

### 2.3. Application of Activity-Based Profiling to Target Discovery 

Based on the results of the potency in cell assay, probe **2d** was selected for photolabeling studies to detect the binding proteins of BAY R3401 by PAGE and chemiluminescence [12]. The soluble proteomes prepared from HepG2 cells, were incubated with the 10 μM probe **2d**, exposed to UV light for 30 min, followed by SDS-PAGE electrophoresis, and then transfer onto a PVDF membrane for detection with streptavidin-HRP [10]. Samples were prepared by incubating 2.0 mg/mL proteomes at different conditions: (Lane 1 and 6) marker; (Lane 2) with the 10 μM probe **2d** and exposed to UV light for 30 min; (Lane 3) with the 10 μM probe **2d** and BAY R3401, and then exposed to UV light for 30 min; (Lane 4) With the 10 μM control compound **32** and exposed to UV light for 30 min; and (Lane 5) with the 0 μM probe **2d** and exposed to UV light for 30 min.

**Figure 3:**
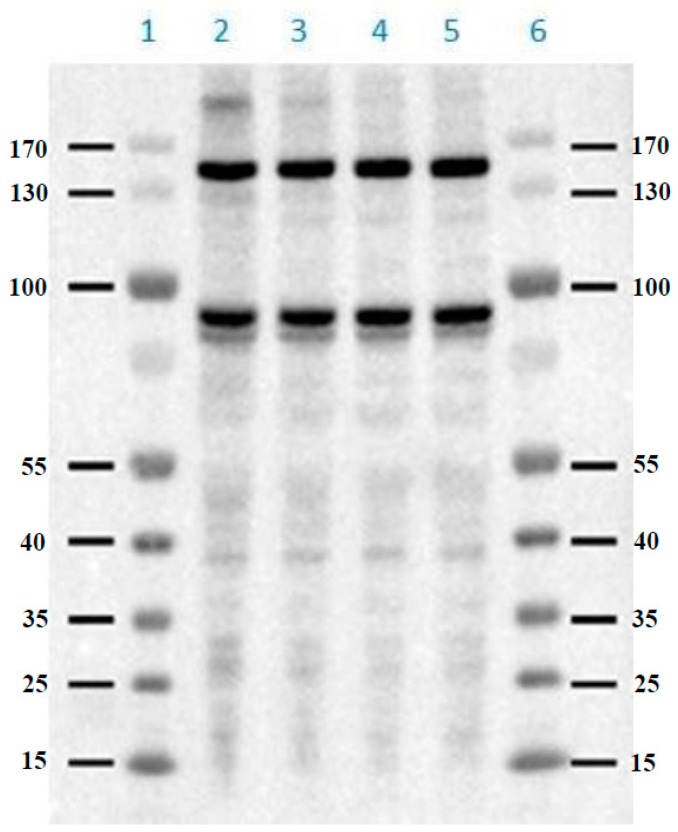
Results of SDS-PAGE analysis of photoaffinity labeling of experiment by synthesized probe **2d**.

The results showed that a protein band larger than 170 kDa was seen in samples incubated with **2d**. This labeling was specific since it was completed by BAY R3401, and no such labeling was seen when samples were incubated with control compound **32** rather than **2d** (Figure 3). These data demonstrate that the synthesized probe **2d** can efficiently and specifically identify the binding protein(s) of BAY R3401 and suggest that **2d** will be suitable for isolating the binding protein(s) of BAY R3401 by avidin–agarose chromatography. 

Then, we used a more detailed proteomic analysis, with a mass spectrometric analysis method, to detect the protein band larger than 170 kDa. Several proteins have been identified, and some of the results are summarized in Table 2. We hypothesise that the PH-interacting protein, histone H4, hexokinase-2 and solute carrier family 2 might be the target proteins of BAY R3401 based on the protein probability scores in MaxQuant. We are now in the process of verifying these proteins. A comparison between the streptavidin blot analysis and Coomassie brilliant blue (CBB) staining was carried out. The detailed information of CBB is in Supplementary information. The results also showed that the streptavidin blot analysis was specific since the protein labeled by probe **2d** is clearly shown after the streptavidin blot analysis while CBB staining had a strong background with the gel image under identical staining conditions.

## 3. Materials and Methods 

### 3.1. Chemistry

NMR experiments were performed on a Bruker Avance III 400 MHz and a Bruker Fourier 300 MHz. The spectra are referenced internally according to the residual solvent signals of TMS (δ = 0.00 ppm). Positive or negative ion LCMS data were obtained at 303 K by a quadrupole Mass Spectrometer on Agilent LC/MSD 1200 Series using a 50 × 4.6 mm (5 μm) ODS column. Prep-HPLC experiments were performed by flash welchrom C18 column (150 × 20 mm) chromatography.

#### 3.1.1. Isopropyl 2-methyl-4-(2-chlorophenyl)-5-oxo-1,4,5,7-tetrahydrofuro [3,4-b]pyridine-3-carboxylate (**10**)

A mixture of **9** (224.9 mL, 1.55 mol), ethyl 4-chloro-3-oxobutanoate (209.4 mL, 1.55 mol) and 2-chlorobenzaldehyde (174.4 mL, 1.55 mol) in isopropyl alcohol (1.5 L) was stirred at reflux temperature overnight. After cooling to r.t. overnight, the mixture was filtered. The residue was purified by recrystallized from isopropyl ether to give product **10** (90.0 g, 17%) as a yellow solid. HPLC analysis: 100.0%. M.p. 225–227 °C. ^1^H-NMR (400 MHz, *d*_6_-DMSO): 0.71 (d, *J* = 8.4 Hz, 3H), 1.12 (d, *J* = 8.4 Hz, 3H), 2.30 (s, 3H), 4.68–4.76 (m, 1H), 4.79 (s, 2H), 5.16 (s, 1H), 7.12–7.18 (m, 1H), 7.25–7.32 (m, 3H), 9.79 (s, 1H). ^13^C-NMR (100 MHz, *d*_6_-DMSO): 171.5, 166.3, 156.7, 146.6, 144.3, 132.4, 131.4, 129.2, 128.2, 127.7, 103.4, 100.7, 66.5, 65.5, 34.8, 22.0, 21.3, 19.2. ESI (MS) *m*/*z*: 348.1 (*M* + H)^+^. HRMS (ESI-TOF) calculated for C_18_H_18_ClNNaO_4_ (*M* + Na)^+^: 370.08166; found: 370.08298.

#### 3.1.2. Isopropyl 2-methyl-4-(2-chlorophenyl)-1-(2-tert-butoxy-2-oxoethyl)-5-oxo-1,4,5,7-tetrahydrofuro[3,4-b]pyridine-3-carboxylate (**11**)

To a solution of **10** (62.60 g, 0.18 mol) in anhydrous DMF (1.5 L) was added a NaH (60% dispersion in mineral oil, 8.85 g, 0.22 mol) at 0 °C under a N_2_ atmosphere. The mixture was heated at 80 °C for 2 h and then tert-butyl 2-chloroacetate (36.10 g, 0.24 mol) was added slowly dropwise at r.t.. The mixture was stirred at 80 °C for 1 h. After cooling to r.t., the mixture was diluted with water (2.5 L) and extracted by EtOAc (1 L × 3). The combined organic phase was washed with brine (1 L × 2), dried over anhydrous Na_2_SO_4_, and evaporated. The residue was purified by being recrystallized with EtOAc (200 mL) to give product **11** (33.0 g, 40%) as a white solid. HPLC analysis: 90.3%. M.p. 174–176 °C. ^1^H-NMR (400 MHz, CDCl_3_): 0.85 (d, *J* = 8.4 Hz, 3H), 1.22 (d, *J* = 6.4 Hz, 3H), 1.55 (s, 9H), 2.42 (s, 3H), 4.07 (dd, *J* = 42.8, 18.4 Hz, 2H), 4.68 (s, 2H), 4.84–4.93 (m, 1H), 5.47 (s, 1H), 7.11 (td, *J* = 7.6, 1.6 Hz, 1H), 7.21 (td, *J* = 7.6, 1.2 Hz, 1H), 7.31 (dd, *J* = 8.0, 1.2 Hz, 1H), 7.42 (dd, *J* = 8.0, 1.6 Hz, 1H). ^13^C-NMR (100 MHz, CDCl_3_): 171.1, 166.9, 166.8, 156.4, 144.9, 142.2, 133.2, 131.1, 129.4, 127.9, 127.0, 109.0, 103.3, 84.0, 67.8, 65.0, 48.1, 35.0, 28.0, 21.7, 20.9, 15.1. ESI (MS) *m*/*z*: 462.0 (*M* + H)^+^. HRMS (ESI-TOF) calculated for C_24_H_29_ClNO_6_ (*M* + H)^+^: 462.16779; found: 462.16878.

#### 3.1.3. 2-[2-Methyl-3-isopropoxycarbonyl-4-(2-chlorophenyl)-5-oxo-furo[3,4-b]pyridine-1(4*H*,5*H*,7*H*)-yl]acetic acid (**12**)

To a solution of **11** (10.0 g, 21.65 mmol) in DCM (100 mL) under a N_2_ atmosphere, TFA (15.0 mL, 0.20 mol) was added, dropwise, at 0 °C. The mixture was stirred at 0 °C for 2 h and then quenched with KHCO_3_ aq (3 N). The aqueous layer was acidified with acetic acid to pH = 2 and extracted by EtOAc (100 mL × 5). The combined organic phase was washed with brine (200 mL × 2), dried over anhydrous Na_2_SO_4_, and concentrated. The residue was purified by flash silica gel column chromatography [CH_2_Cl_2_-MeOH (100:1)] to obtain a white solid product 12 (4.0 g, 45%). HPLC analysis: 92.6%. M.p. 155–157 °C. ^1^H-NMR (400 MHz, *d*_6_-DMSO): 0.79 (d, *J* = 6.0 Hz, 3H), 1.14 (d, *J* = 6.4 Hz, 3H), 2.32 (s, 3H), 4.27 (q, *J* = 18.4 Hz, 2H), 4.71–4.78 (m, 1H), 4.87 (dd, *J* = 42.8, 16.4 Hz, 2H), 5.22 (s, 1H), 7.17 (td, *J* = 7.6, 1.6 Hz, 1H), 7.27 (td, *J* = 7.2, 0.8 Hz, 1H), 7.32 (dd, *J* = 7.6, 0.8 Hz, 1H), 7.49 (dd, *J* = 7.6, 0.8 Hz, 1H). ^13^C-NMR (100 MHz, *d*_6_-DMSO): 171.5, 170.9, 166.9, 159.1, 146.9, 143.7, 132.3, 131.6, 129.1, 128.4, 127.8, 107.4, 101.1, 67.2, 66.0, 48.6, 34.6, 21.9, 21.2, 15.3. ESI (MS) *m*/*z*: 405.9 (*M* + H)^+^. HRMS (ESI-TOF) calculated for C_20_H_20_ClNNaO_6_ (*M* + Na)^+^: 428.08714; found: 428.08765.

#### 3.1.4. General Procedure for Synthesis of compounds **13a**–**13d**

To a solution of **12** (12.20 g, 0.03 mol) in anhydrous acetonitrile (120 mL) was added K_2_CO_3_ (12.50 g, 0.09 mol) in groups, and then Bromo-PEG(n)-bromide (0.15 mol) was added to the reaction mixture slowly under an ice bath, and the temperature was raised to 80 °C for 1.5 h. After cooling to r.t., the mixture was diluted with water (150 mL) and extracted by EtOAc (100 mL × 2). The combined organic phase was washed with brine (100 mL × 2), dried over anhydrous Na_2_SO_4_, and evaporated. The residue was purified by flash column chromatography [petroleum ether-EtOAc (1:1)] to give the products **13a**–**13d**.

*Isopropyl 1-[2-(2-bromoethoxy)-2-oxoethyl]-4-(2-chlorophenyl)-2-methyl-5-oxo-1, 4, 5, 7-tetrahydrofuro[3,4-b]pyridine-3-carboxylate* (**13a**). HPLC analysis: 96.8%. M.p. 154–156 °C. ^1^H-NMR (400 MHz, CDCl_3_): 0.85 (d, *J* = 6.4 Hz, 3H), 1.20 (d, *J* = 6.4 Hz, 3H), 2.41 (s, 3H), 3.58 (t, *J* = 6.0 Hz, 2H), 4.25 (dd, *J* = 57.6, 18.8 Hz, 2H), 4.57 (t, *J* = 5.6 Hz, 2H), 4.69 (s, 2H), 4.85–4.91 (m, 1H), 5.44 (s, 1H), 7.11 (td, *J* = 8.0, 1.6 Hz, 1H), 7.21 (t, *J* = 7.6, Hz, 1H), 7.30 (d, *J* = 7.6, 1H), 7.41 (dd, *J* = 7.6, 1.6 Hz, 1H). ^13^C-NMR (100 MHz, CDCl_3_): 171.0, 167.6, 166.7, 156.2, 144.4, 141.9, 133.3, 131.1, 129.4, 127.9, 127.0, 109.4, 103.6, 67.9, 65.3, 65.0, 47.2, 35.1, 28.3, 21.7, 20.9, 15.2. ESI (MS) *m*/*z*: 513.8 (*M* + H)^+^. HRMS (ESI-TOF) calculated for C_22_H_24_BrClNO_6_ (*M* + H)^+^: 512.047; found: 512.04884.

*Isopropyl 1-{2-[2-(2-bromoethoxy)ethoxy]-2-oxoethyl}-4-(2-chlorophenyl)-2-methyl-5-oxo-1, 4, 5, 7-tetrahydrofuro[3, 4-b]pyridine-3-carboxylate* (**13b**). HPLC analysis: 90.0%. M.p. 113–115 °C. ^1^H-NMR (400 MHz, CDCl_3_): 0.85 (d, *J* = 6.4 Hz, 3H), 1.21 (d, *J* = 6.4 Hz, 3H), 2.42 (s, 3H), 3.47 (t, *J* = 6.0 Hz, 2H), 3.78 (t, *J* = 4.8 Hz, 2H), 3.82 (t, *J* = 5.6 Hz, 2H), 4.24 (dd, *J* = 51.2, 18.8 Hz, 2H), 4.43–4.46 (m, 2H), 4.70 (s, 2H), 4.83–4.93 (m, 1H), 5.45 (s, 1H), 7.11 (td, *J* = 8.0, 1.6 Hz, 1H), 7.21 (td, *J* = 7.6, 1.2 Hz, 1H), 7.31 (dd, *J* = 7.6, 1.2 Hz, 1H), 7.41 (dd, *J* = 7.6, 1.6 Hz, 1H). ^13^C-NMR (100 MHz, CDCl_3_): 171.1, 167.9, 166.8, 156.4, 144.6, 142.0, 133.2, 131.1, 129.4, 127.9, 127.0, 109.3, 103.5, 71.0, 68.5, 67.9, 65.1, 64.8, 47.3, 35.0, 30.3, 21.7, 20.9, 15.2. ESI (MS) *m*/*z*: 557.9 (*M* + H)^+^. HRMS (ESI-TOF) calculated for C_24_H_28_BrClNO_7_ (*M* + H)^+^: 556.07322; found: 556.07444.

*Isopropyl 1-(2-{2-[2-(2-bromoethoxy)ethoxy]ethoxy}-2-oxoethyl)-4-(2-chlorophenyl)-2-methyl-5-oxo-1,4,5,7-tetrahydrofuro[3,4-b]pyridine-3-carboxylate* (**13c**). HPLC analysis: 94.9%. M.p. 77–78 °C. ^1^H-NMR (400 MHz, CDCl_3_): 0.85 (d, *J* = 6.0 Hz, 3H), 1.21 (d, *J* = 6.4 Hz, 3H), 2.42 (s, 3H), 3.49 (t, *J* = 6.0 Hz, 2H), 3.67 (s, 4H), 3.77–3.82 (m, 4H), 4.24 (dd, *J* = 51.2, 18.8 Hz, 2H), 4.43–4.45 (m, 2H), 4.71 (s, 2H), 4.84–4.93 (m, 1H), 5.46 (s, 1H), 7.11 (td, *J* = 7.6, 1.6 Hz, 1H), 7.21 (td, *J* = 7.6, 0.8 Hz, 1H), 7.31 (dd, *J* = 8.0, 0.8 Hz, 1H), 7.41 (dd, *J* = 7.6, 1.6 Hz, 1H). ^13^C-NMR (100 MHz, CDCl_3_): 171.1, 167.9, 166.8, 156.4, 144.7, 142.0, 133.3, 131.1, 129.4, 127.9, 127.0, 109.3, 103.6, 71.2, 70.5, 68.8, 67.9, 65.1, 47.3, 35.1, 30.5, 21.7, 20.9, 15.2. ESI (MS) *m*/*z*: 602.0 (*M* + H)^+^. HRMS (ESI-TOF) calculated for C_26_H_31_BrClNNaO_8_ (*M* + Na)^+^: 622.08138; found: 622.08153.

*Isopropyl 1-(14-bromo-2-oxo-3,6,9,12-tetraoxatetradecyl)-4- (2-chlorophenyl)-2-methyl-5-oxo-1,4,5,7-tetrahydrofuro[3,4-b]pyridine-3-carboxylate* (**13d**). HPLC analysis: 92.7%. ^1^H-NMR (400 MHz, CDCl_3_): 0.85 (d, *J* = 6.0 Hz, 3H), 1.21 (d, *J* = 6.4 Hz, 3H), 2.41 (s, 3H), 3.49 (t, *J* = 6.4Hz, 2H), 3.63–3.69 (m, 8H), 3.76 (t, *J* = 4.8 Hz, 2H), 3.81 (t, *J* = 6.4Hz, 2H), 4.24 (dd, *J* = 48.8, 18.4 Hz, 2H), 4.42–4.44 (m, 2H), 4.71 (s, 2H), 4.83–4.92 (m, 1H), 5.45 (s, 1H), 7.11 (td, *J* = 8.0, 1.6 Hz, 1H), 7.21 (td, *J* = 7.6, 1.2 Hz, 1H), 7.31 (dd, *J* = 7.6, 1.2 Hz, 1H), 7.41 (dd, *J* = 7.6, 1.6 Hz, 1H). ^13^C-NMR (100 MHz, CDCl_3_): 171.1, 167.9, 166.8, 156.5, 144.7, 142.1, 133.2, 131.1, 129.3, 127.9, 127.0, 109.2, 103.5, 71.1, 70.6, 70.5, 70.4, 68.7, 67.8, 65.1, 65.0, 47.3, 35.0, 30.5, 21.7, 20.9, 15.2. ESI (MS) *m*/*z*: 646.1 (*M* + H)^+^. HRMS (ESI-TOF) calculated for C_28_H_35_BrClNNaO_9_ (*M* + Na)^+^: 666.10759; found: 666.10903.

#### 3.1.5. General Procedure for Synthesis of Compounds **6a**–**6d**

A mixture of **13a**–**13d** (1.0 equiv.) and NaN_3_ (1.5 equiv.) in DMF (20 mL) was stirred at r.t. for 26 h. The mixture was diluted with ice water (100 mL) and extracted by EtOAc (100 mL × 2). The combined organic phase was washed with brine (100 mL × 2), dried over anhydrous Na_2_SO_4_, and concentrated. The residue was purified by flash column chromatography [petroleum ether-EtOAc (2:1)] to give products **6a**–**6d** as white solids.

*1-[2-(2-Azidoethoxy)-2-oxoethyl]-4-(2-chlorophenyl)-2-methyl-5-oxo-1, 4, 5, 7-tetrahydrofuro[3,4-b]pyridine-3-carboxylate* (**6a**). HPLC analysis: 94.2%. M.p. 142–144 °C. ^1^H-NMR (400 MHz, CDCl_3_): 0.86 (d, *J* = 6.0 Hz, 3H), 1.21 (d, *J* = 6.4 Hz, 3H), 2.42 (s, 3H), 3.59 (t, *J* = 4.8Hz, 2H), 4.25 (dd, *J* = 54.0, 18.4 Hz, 2H), 4.44 (t, *J* = 4.8 Hz, 2H), 4.69 (s, 2H), 4.84–4.93 (m, 1H), 5.46 (s, 1H), 7.12 (td, *J* = 7.6, 1.6 Hz, 1H), 7.22 (t, *J* = 7.6, 1H), 7.31 (d, *J* = 7.6, Hz, 1H), 7.42 (dd, *J* = 7.6, 1.2 Hz, 1H). ^13^C-NMR (100 MHz, CDCl_3_): 171.0, 167.8, 166.7, 156.2, 144.4, 141.9, 133.3, 131.1, 129.4, 127.9, 127.0, 109.5, 103.7, 67.9, 65.0, 64.6, 49.5, 47.1, 35.1, 21.7, 20.9, 15.2. ESI (MS) *m*/*z*: 474.9 (*M* + H)^+^. HRMS (ESI-TOF) calculated for C_22_H_24_ClN_4_O_6_ (*M* + H)^+^: 475.13789; found: 475.13970.

*Isopropyl 1-{2-[2-(2-azidoethoxy)ethoxy]-2-oxoethyl}-4-(2-chlorophenyl)-2-methyl-5-oxo-1, 4, 5, 7-tetrahydrofuro[3,4-b]pyridine-3-carboxylate* (**6b**). HPLC analysis: 96.8%. M.p. 118–120 °C. ^1^H-NMR (400 MHz, CDCl_3_): 0.84 (d, *J* = 6.4 Hz, 3H), 1.19 (d, *J* = 6.4 Hz, 3H), 2.39 (s, 3H), 3.37 (t, *J* = 4.8 Hz, 2H), 3.68 (t, *J* = 4.8 Hz, 2H), 3.75 (t, *J* = 4.4 Hz, 2H), 4.22 (dd, *J* = 57.2, 18.8 Hz, 2H), 4.42 (t, *J* = 7.6 Hz, 2H), 4.66 (s, 2H), 4.82–4.91 (m, 1H), 5.44 (s, 1H), 7.10 (td, *J* = 8.0, 1.6 Hz, 1H), 7.20 (t, *J* = 7.6 Hz, 1H), 7.29 (d, *J* = 7.2 Hz, 1H), 7.41 (dd, *J* = 7.6, 1.2 Hz, 1H). ^13^C-NMR (100 MHz, CDCl_3_): 171.2, 168.0, 166.8, 156.6, 144.7, 142.2, 133.2, 131.2, 129.3, 127.9, 127.1, 109.2, 103.4, 70.2, 68.6, 67.8, 65.1, 64.9, 50.5, 47.2, 35.0, 21.7, 20.9, 15.2. ESI (MS) *m*/*z*: 518.9 (*M* + H)^+^. HRMS (ESI-TOF) calculated for C_24_H_28_ClN_4_O_7_ (*M* + H)^+^: 519.1641; found: 519.16577.

*Isopropyl 1-(2-{2-[2-(2- azidoethoxy)ethoxy]ethoxy}-2-oxoethyl)-4-(2-chlorophenyl)-2-methyl-5-oxo-1, 4, 5, 7-tetrahydrofuro[3, 4-b]pyridine-3-carboxylate* (**6c**). HPLC analysis: 94.0%. M.p. 44–45 °C. ^1^H-NMR (400 MHz, CDCl_3_): 0.85 (d, *J* = 6.0 Hz, 3H), 1.21 (d, *J* = 6.4 Hz, 3H), 2.42 (s, 3H), 3.39 (t, *J* = 4.8Hz, 2H), 3.63–3.68 (m, 6H), 3.78 (t, *J* = 4.8Hz, 2H), 4.23 (dd, *J* = 51.6, 18.8 Hz, 2H), 4.43–4.45 (m, 2H), 4.70 (s, 2H), 4.83–4.93 (m, 1H), 5.46 (s, 1H), 7.11 (td, *J* = 7.6, 1.6 Hz, 1H), 7.21 (td, *J* = 7.6, 1.2 Hz, 1H), 7.31 (dd, *J* = 7.6, 1.2 Hz, 1H), 7.41 (dd, *J* = 7.6, 1.6 Hz, 1H). ^13^C-NMR (100 MHz, CDCl_3_): 171.1, 167.9, 166.8, 156.4, 144.7, 142.0, 133.3, 131.1, 129.4, 127.9, 127.0, 109.3, 103.5, 70.7, 70.6, 70.1, 68.8, 67.8, 65.1, 65.0, 50.6, 47.3, 35.1, 21.7, 20.9, 15.2. ESI (MS) *m*/*z*: 563.1 (*M* + H)^+^. HRMS (ESI-TOF) calculated for C_26_H_31_ClN_4_NaO_8_ (*M* + Na)^+^: 585.17226; found: 585.17324.

*Isopropyl 1-(14-azido-2-oxo-3,6,9,12-tetraoxatetradecyl)-4-(2-chlorophenyl)-2-methyl-5-oxo-1,4,5,7-tetrahydrofuro[3,4-b]pyridine-3-carboxylate* (**6d**). HPLC analysis: 94.3%. M.p. 37–38 °C. ^1^H-NMR (400 MHz, CDCl_3_): 0.84 (d, *J* = 6.4 Hz, 3H), 1.20 (d, *J* = 6.4 Hz, 3H), 2.41 (s, 3H), 3.39 (t, *J* = 4.8 Hz, 2H), 3.65–3.68 (m, 10H), 3.75 (t, *J* = 4.4 Hz, 2H), 4.22 (dd, *J* = 50.4, 18.4 Hz, 2H), 4.41–4.43 (m, 2H), 4.70 (s, 2H), 4.84–4.90 (m, 1H), 5.45 (s, 1H), 7.11 (td, *J* = 7.6, 1.6 Hz, 1H), 7.21 (td, *J* = 7.6, 1.2 Hz, 1H), 7.30 (d, *J* = 8.0 Hz, 1H), 7.41 (dd, *J* = 7.6, 1.2 Hz, 1H). ^13^C-NMR (100 MHz, CDCl_3_): 171.1, 167.9, 166.8, 156.5, 144.7, 142.1, 133.2, 131.1, 129.3, 127.9, 127.0, 109.2, 103.5, 70.7, 70.6, 70.5, 70.4, 70.0, 68.7, 67.8, 65.1, 65.0, 50.7, 47.3, 35.0, 21.7, 20.9, 15.2. ESI (MS) *m*/*z*: 607.1 (*M* + H)^+^. HRMS (ESI-TOF) calculated for C_28_H_35_ClN_4_NaO_9_ (*M* + Na)^+^: 629.19848; found: 629.19933.

#### 3.1.6. (*S*)-Methyl 2-(4-benzoylbenzamido)-6-(tert-butoxycarbonylamino)hexanoate (**16**)

Lys(Boc)-OMe·HCl (0.20 g, 0.67 mmol) was dissolved in 20 mL of DMF, to which 4-benzoylbenzoic acid (0.15 g, 0.67 mmol), *N*-[3-(dimethylamino)propyl]-*N*-ethylcarbodiimide hydrochloride (EDCI, 0.15 g,0.78 mmol), 4-(dimethylamino)pyridine (DMAP, 0.097 g, 0.79 mmol), and *N*-methylmorpholine (NMM, 0.26 mL, 2.36 mmol) were added. The reaction mixture was stirred at r.t. overnight and then poured into ice water (50 mL). The aqueous layer was extracted with EtOAc (50 mL × 2). The combined organic layer was washed with saturated brine (50 mL × 2), dried over anhydrous Na_2_SO_4_, filtered, and concentrated. The residue was purified by flash silica gel column chromatography [petroleum ether-EtOAc (4:1)] to give benzophenone-Lys(Boc)-OMe as a transparent solid (0.28 g, 89%). HPLC analysis: 98.6%. M.p. 66–67 °C. ^1^H-NMR (400 MHz, CDCl_3_): 1.40(s, 9H), 1.45–1.58 (m, 4H), 1.83–2.04 (m, 2H), 3.06–3.18 (m, 2H), 3.80 (s, 3H), 4.64 (br s, 1H), 4.79–4.84 (m, 1H), 6.97 (d, *J* = 5.6 Hz, 1H), 7.50 (t, *J* = 7.6 Hz, 2H), 7.62 (t, *J* = 7.2 Hz, 1H), 7.80 (d, *J* = 7.6 Hz, 2H), 7.85 (d, *J* = 8.0 Hz, 2H), 7.94 (d, *J* = 8.4 Hz, 2H). ^13^C-NMR (100 MHz, CDCl_3_): 195.9, 172.9, 166.4, 156.2, 140.3, 137.1, 137.0, 132.9, 130.1, 128.4, 127.2, 79.2, 52.64, 52.59, 40.0, 32.0, 29.7, 28.4, 22.5. ESI (MS) *m*/*z*: 491.2 (*M* + Na)^+^. HRMS (ESI-TOF) calculated for C_26_H_32_N_2_NaO_6_ (*M* + Na)^+^: 491.2158; found: 491.2164.

#### 3.1.7. (*S*)-Prop-2-ynyl 2-(4-benzoylbenzamido)-6-(tert-butoxycarbonylamino)hexanoate (**18**)

To a solution of **16** (0.50 g, 1.07 mmol) in MeOH (10 mL), a NaOH aqueous (2 N, 10 mL) was added under an ice bath. The reaction mixture was stirred at r.t. for 3 h and then concentrated in vacuo. The residue was redissolved in H_2_O (20 mL), and then the aqueous was adjusted to pH = 2 with HCl aqueous (1 N), the suspension was extracted with EtOAc (20 mL × 3), and the combined organic layer was dried over anhydrous Na_2_SO_4_, filtered, and concentrated to give a white solid product (0.37 g, 76%), which was used directly in the next reaction without further purification. The above product **17** (10.0 g, 22.0 mmol) and K_2_CO_3_ (6.08 g, 44.0 mmol) in anhydrous DMF (120 mL) were slowly added to 3-Bromopropyne (2.85 mL, 33.04 mmol, 80% in toluene). The reaction mixture was stirred at r.t. overnight and then filtered. The filtrate was diluted with water (200 mL) and extracted with EtOAc (200 mL × 2). The combined organic layer was washed with 1 N HCl aqueous solution (200 mL), saturated NaHCO_3_ solution (200 mL), and saturated brine (200 mL), dried over anhydrous Na_2_SO_4_ and evaporated. The mixture was purified by column chromatography on silica gel [petroleum ether-EtOAc (4:1)] to give **18** (9.70 g, 90%) of a white solid. HPLC analysis: 98.6%. M.p. 79–81 °C. ^1^H-NMR (400 MHz, CDCl_3_): 1.40 (s, 8H), 1.53 (ddd, *J* = 26.4, 14.9, 7.2 Hz, 4H), 1.89 (dt, *J* = 13.2, 8.4 Hz, 1H), 1.96–2.10 (m, 1H), 2.53 (s, 1H),3.13 (s, 2H), 4.61 (s, 1H), 4.74 (dd, *J* = 15.4, 2.2 Hz, 1H), 4.85 (dt, *J* = 11.2, 4.0 Hz, 2H), 6.94 (d, *J* = 6.0 Hz, 1H), 7.50 (t, *J* = 7.6 Hz, 2H), 7.62 (t, *J* = 7.4 Hz, 1H), 7.80 (d, *J* = 7.6 Hz, 2H), 7.85 (d, *J* = 8.0 Hz, 2H), 7.94 (d, *J* = 8.2 Hz, 2H). ^13^C-NMR(100 MHz, CDCl_3_): 195.9, 171.7, 166.5, 156.2, 140.4, 137.1, 137.0, 132.9, 130.1, 128.5, 127.2, 79.2, 75.6, 52.9, 52.6, 39.9, 31.8, 29.7, 28.4, 22.4. ESI (MS) *m*/*z*: 515.2 (*M* + Na)^+^. HRMS (ESI-TOF) calculated for C_28_H_32_N_2_NaO_6_ (*M* + Na)^+^: 515.2158; found: 515.2165. 

#### 3.1.8. General Procedure for Synthesis of compounds **20** and **32**

The crude product **19** or **23** (12.80 mmol) and D-Biotin (19.50 mmol) were dissolved in 200 mL of anhydrous DMF, to which EDCI (0.69 mmol), HOBt (0.69 mmol), and DIPEA (1.38 mmol) were added at 0 °C. The mixture was stirred at r.t. overnight under a N_2_ atmosphere and then diluted with water (10 mL) and extracted with EtOAc (10 mL × 3). The combined organic layer was washed with brine (10 mL × 2), dried over Na_2_SO_4_, filtered, and concentrated in vacuo. The crude product was purified by column chromatography over silica gel [CH_2_Cl_2_-MeOH (40:1)] to give target products **20** and **32** as white solids.

*(S)-Prop-2-ynyl 2-(4-benzoylbenzamido)-6-(5-{(3aS,4S,6aR)-2-oxohexahydro-1H-thieno[3,4-d]imidazole-4-yl}pentanamido)hexanoate* (**20**). HPLC analysis: 95.4%. M.p. 130–132 °C. ^1^H-NMR (400 MHz, *d*_6_-DMSO): 1.30–1.59 (m, 10H), 1.83 (br *s*, 2H), 2.04 (t, *J* = 5.6 Hz, 2H), 2.57 (d, *J* = 12.4 Hz, 1H), 2.80–2.83 (m, 1H), 3.05 (br *s*, 3H), 3.63 (d, *J* = 36.0 Hz, 1H), 4.12 (br s, 1H), 4.30 (br s, 1H), 4.44–4.49 (m, 1H), 4.77 (br *s*, 2H), 6.35 (br s, 1H), 6.40 (br s, 1H), 7.55–7.64 (m, 2H), 7.72–7.84 (m, 5H), 8.06 (d, *J* = 7.2 Hz, 1H), 8.97 (d, *J* = 6.4 Hz, 1H). ^13^C-NMR (100 MHz, *d*_6_-DMSO): 195.4, 171.9, 171.4, 162.7, 139.4, 136.9, 136.6, 133.0, 129.7, 129.4, 128.7, 127.7, 78.2, 77.9, 61.0, 59.2, 55.4, 52.7, 52.2, 38.0, 35.2, 29.9, 28.7, 28.2, 28.0, 25.3, 23.1, 23.0. ESI (MS) *m*/*z*: 641.2 (*M* + H)^+^. HRMS (ESI-TOF) calculated for C_33_H_38_N_4_NaO_6_ (*M* + Na)^+^: 641.2410; found: 641.2418.

*(S)-Methyl 2-(4-benzoylbenzamido)-6-(5-{(3aS,4S,6aR)-2-oxohexahydro-1H-thieno[3,4-d]imidazole-4-yl}pentanamido)hexanoate* (**32**). HPLC analysis: 90.2%. M.p. 148–150 °C. ^1^H-NMR (400 MHz, CDCl_3_): 1.34–1.62 (m, 10H), 1.92–1.95 (m, 2H), 2.16–2.18 (m, 2H), 2.69 (d, *J* = 12.4 Hz, 1H), 2.84–2.85 (m, 1H), 3.07–3.08 (m, 1H), 3.24 (br *s*, 2H), 3.77 (s, 3H), 4.22–4.29 (m, 1H), 4.44–4.50 (m, 1H), 4.73 (dd, *J* = 13.2, 8.0 Hz, 1H), 6.40 (br *s*, 1H), 6.65 (br *s*, 1H), 7.49 (t, *J* = 8.0 Hz, 2H), 7.59–7.66 (m, 2H), 7.80 (dd, *J* = 16.8, 7.2 Hz, 4H), 8.03 (d, *J* = 8.0 Hz, 2H). ^13^C-NMR (100 MHz, *d*_6_-DMSO): 195.4, 172.6, 171.9, 165.9, 162.7, 139.4, 137.0, 136.6, 133.0, 129.7, 129.4, 128.7, 127.7, 61.0, 59.1, 55.4, 52.8, 51.9, 38.0, 35.2, 33.5, 30.1, 28.7, 28.0, 25.3, 24.5, 23.1. ESI (MS) *m*/*z*: 595.3 (*M* + H)^+^. HRMS (ESI-TOF) calculated for C_31_H_39_N_4_O_6_S (*M* + H)^+^: 595.2590; found: 595.2598.

#### 3.1.9. General Procedure for Synthesis of Compounds **21** and **24**

To a solution of **16** or **18** (21.34 mmol) in anhydrous CH_2_Cl_2_ (50 mL), trifluoroacetic acid (TFA, 32 mL, 0.43 mol) was added. The reaction mixture was stirred at 0 °C for 2 h. Then, the reaction mixture was concentrated in vacuo. The residue was redissolved in EtOAc (20 mL) and washed with saturated NaHCO_3_ (20 mL), 1 N HCl aqueous (20 mL), and brine (20 mL), dried over Na_2_SO_4_, filtered, and concentrated to give the crude target product **19** or **23** as a white solid, which was used for the next reaction without further purification. To a mixture of the above crude product **19** or **23** (21.71 mmol) and Et_3_N (4.75 mL, 34.22 mmol) in anhydrous CH_2_Cl_2_/CH_3_OH (1:1, *v*/*v*, 200 mL) were slowly added dansyl chloride (9.23 g, 34.22 mmol) in groups under an ice bath. The reaction mixture was stirred at r.t. overnight. After the solvent was removed in vacuo, the residue was redissolved in CH_2_Cl_2_ (20 mL) and washed with brine (2 × 20 mL), dried over anhydrous Na_2_SO_4_, and evaporated. The mixture was purified by column chromatography on silica gel [CH_2_Cl_2_ = 100%] to give target the products **21** and **24** as pale yellow solids.

*(S)-Prop-2-ynyl-6-dansylamide-2-(4-benzoylbenzamido)hexanoate* (**21**). HPLC analysis: 100.0%. M.p. 76–78 °C. ^1^H-NMR (400 MHz, CDCl_3_): 1.43–1.56 (m, 4H), 1.73–1.82 (m, 1H), 1.85–1.92 (m, 1H), 2.52 (t, *J* = 6.4 Hz, 1H), 2.88 (s, 6H), 2.90–2.94 (m, 2H), 4.69–4.83 (m, 3H), 5.05 (br t, *J* = 6.0 Hz, 1H), 6.97 (d, *J* = 7.6 Hz, 1H), 7.16 (d, *J* = 7.2 Hz, 1H), 7.46–7.52 (m, 4H), 7.60 (tt, *J* = 7.6, 1.2 Hz, 1H), 7.77–7.83 (m, 4H), 7.94 (d, *J* = 8.4 Hz, 2H), 8.20 (dd, *J* = 7.2, 0.8 Hz, 1H), 8.28 (d, *J* = 8.4 Hz, 1H), 8.54 (d, *J* = 8.4 Hz, 1H). ^13^C-NMR (100 MHz, CDCl_3_): 195.9, 171.6, 166.6, 140.4, 137.0, 136.8, 134.8, 132.9, 130.4, 130.1, 129.9, 129.6, 129.5, 128.5, 128.4, 127.3, 123.2, 115.3, 77.0, 75.7, 52.9, 52.5, 45.4, 42.6, 31.5, 28.8, 22.0. ESI (MS) *m*/*z*: 648.2 (*M* + Na)^+^. HRMS (ESI-TOF) calculated for C_35_H_35_N_3_NaO_6_S (*M* + Na)^+^: 648.2144; found: 648.2152.

*(S)-Methyl-6-dansylamide-2-(4-benzoylbenzamido)hexanoate* (**24**). HPLC analysis: 98.1%. M.p. 69–71 °C. ^1^H-NMR (400 MHz, CDCl_3_): 1.39–1.55 (m, 4H), 1.70–1.79 (m, 1H), 1.82–1.90 (m, 1H), 2.88–2.92 (m, 8H), 3.76 (s, 3H), 4.71–4.76 (m, 1H), 5.13 (br t, *J* = 5.6 Hz, 1H), 6.99 (d, *J* = 7.6 Hz, 1H), 7.16 (d, *J* = 7.2 Hz, 1H), 7.47–7.52 (m, 4H), 7.61 (t, *J* = 7.6 Hz, 1H), 7.78 (d, *J* = 7.2 Hz, 2H), 7.83 (d, *J* = 8.0 Hz, 2H), 7.95 (d, *J* = 8.4 Hz, 2H), 8.21 (dd, *J* = 7.2, 0.8 Hz, 1H), 8.29 (d, *J* = 8.8 Hz, 1H), 8.53 (d, *J* = 8.4 Hz, 1H). ^13^C-NMR (100 MHz, CDCl_3_): 196.0, 172.8, 166.5, 140.3, 137.0, 136.9, 134.8, 132.9, 130.4, 130.1, 129.8, 129.6, 129.5, 128.5, 128.4, 127.3, 123.2, 115.3, 52.6, 52.5, 45.4, 42.6, 31.7, 28.9, 22.1. ESI (MS) *m*/*z*: 602.3 (*M* + H)^+^. HRMS (ESI-TOF) calculated for C_33_H_35_N_3_NaO_6_S (*M* + Na)^+^: 624.2144; found: 624.2152.

#### 3.1.10. General Procedure for Synthesis of Compounds **22** and **33**

To a solution of **16** or **18** (15.0 g, 30.45 mmol) in anhydrous CH_2_Cl_2_ (150 mL), trifluoroacetic acid (TFA, 46.0 mL, 0.62 mol) was added. The reaction mixture was stirred at 0 °C for 2 h. Then, the reaction mixture was concentrated in vacuo. The residue was redissolved in EtOAc (150 mL) and was washed with saturated NaHCO_3_ (200 mL × 2), 1 N HCl aqueous solution (200 mL × 2), and brine (200 mL × 2), and dried over Na_2_SO_4_, filtered, and concentrated to give the crude target product as a white solid, which was used for next reaction without further purification. To a mixture of the above crude product **19** or **23** (0.26 mmol) and Et_3_N (0.36 mmol) in anhydrous CH_2_Cl_2_/CH_3_OH (1:1, *v*/*v*, 20 mL), was slowly added acyl chloride (0.36 mmol) in groups under an ice bath. The reaction mixture was stirred at r.t. for another 12 h. After the solvent was removed in vacuo, the residue was redissolved in CH_2_Cl_2_ (20 mL) and washed with brine (2 × 20 mL), dried over anhydrous Na_2_SO_4,_ and evaporated. The mixture was purified by column chromatography on silica gel [CH_2_Cl_2_ = 100%] to give target products **22** and **33** as pale yellow solids.

*(S)-Prop-2-ynyl-2-(4-benzoylbenzamido)-6-(2-chloroacetamido)hexanoate* (**22**). HPLC analysis: 99.5%. M.p. 123–124 °C. ^1^H-NMR (400 MHz, CDCl_3_): 1.47 (ddd, *J* = 29.2, 15.8, 6.8 Hz, 2H), 1.65 (tt, *J* = 13.6, 6.8 Hz, 2H), 1.87–1.98 (m, 1H), 2.05 (qd, *J* = 10.6, 5.4 Hz, 1H), 2.53 (t, *J* = 2.2 Hz, 1H), 3.26–3.43 (m, 2H), 3.94–4.06 (m, 2H), 4.75 (dd, *J* = 15.4, 2.4 Hz, 1H), 4.84 (ddd, *J* = 13.2, 5.4, 2.6 Hz, 2H), 6.67 (s, 1H), 6.98 (d, *J* = 7.4 Hz, 1H), 7.50 (t, *J* = 7.6 Hz, 2H), 7.62 (t, *J* = 7.4 Hz, 1H), 7.79 (d, *J* = 7.4 Hz, 2H), 7.85 (d, *J* = 8.2 Hz, 2H), 7.96(d, *J* = 8.2 Hz, 2H). ^13^C-NMR(100 MHz, CDCl_3_): 195.9, 171.6, 166.5, 166.3, 140.4, 137.0, 136.8, 132.9, 130.1, 128.5, 127.2, 77.0, 75.6, 53.0, 52.5, 42.6, 39.1, 31.5, 28.9, 22.3. ESI (MS) *m*/*z*: 491.1 (*M* + Na)^+^. HRMS (ESI-TOF) calculated for C_25_H_25_ClN_2_NaO_5_ (*M* + Na)^+^: 491.1350; found: 491.1356.

*(S)-Methyl-2-(4-benzoylbenzamido)-6-(2-chloroacetamido)hexanoate* (**33**). HPLC analysis: 99.6%. M.p. 129–130 °C. ^1^H-NMR (400 MHz, CDCl_3_): 1.39–1.67 (m, 4H), 1.84–2.06 (m, 2H),3.25–3.41 (m, 2H), 3.80 (s, 3H), 3.95–4.06 (m, 2H), 4.81 (td, *J* = 7.6, 5.0 Hz, 1H), 6.66 (s, 1H), 6.96 (d, *J* = 7.4 Hz, 1H), 7.50 (t, *J* = 7.6 Hz, 2H), 7.62 (t, *J* = 7.4 Hz, 1H), 7.80 (d, *J* = 7.2 Hz, 2H), 7.85 (d, *J* = 8.2 Hz, 2H), 7.96 (d, *J* = 8.2 Hz, 2H). ^13^C-NMR (100 MHz, CDCl_3_): 195.9, 172.8, 166.4, 166.2, 140.4, 137.0, 136.9, 132.9, 130.1, 128.5, 127.2, 52.6, 52.5, 42.6, 39.1, 31.8, 28.9, 22.4. ESI (MS) *m*/*z*: 443.1 (*M* − H)^−^. HRMS (ESI-TOF) calculated for C_23_H_24_ClN_2_O_5_ (*M −* H)^−^: 443.1374; found: 443.1380.

#### 3.1.11. (*S*)-6-Dansylamide-2-(4-benzoylbenzamido)hexanoic acid (**25**) 

To a solution of **24** (5.30 g, 8.81 mmol) in THF (50 mL), LiOH·H_2_O (6 N, 3.0 mL) was added under an ice bath. The reaction mixture was stirred at r.t. for 3 h and then concentrated in vacuo. The residue was redissolved in H_2_O (50 mL), and then the aqueous solution was adjusted to pH = 2 with 1 N HCl aqueous solution, and the suspension was extracted with EtOAc (50 mL × 3). The combined organic layer was dried over anhydrous Na_2_SO_4_, filtered, and concentrated. The residue was purified by flash silica gel column chromatography [CH_2_Cl_2_-MeOH (40:1)] to obtain the pale yellow solid product **25** (4.50 g, 87%). HPLC analysis: 99.5%. M.p. 83–85 °C. ^1^H-NMR (400 MHz, CDCl_3_): 1.40–1.51 (m, 4H), 1.84–2.06 (m, 2H), 2.90–2.97 (m, 8H), 4.75–4.81 (m, 2H), 6.85 (d, *J* = 6.8 Hz, 1H), 7.18 (d, *J* = 10.0 Hz, 1H), 7.52 (dd, *J* = 20.4, 10.0 Hz, 4H), 7.63 (t, *J* = 9.6 Hz, 1H), 7.82 (d, *J* = 9.6 Hz, 2H), 7.92 (dd, *J* = 32.8, 11.2 Hz, 4H), 8.26 (dd, *J* = 15.2, 10.4 Hz, 2H), 8.55 (d, *J* = 11.2 Hz, 1H). ^13^C-NMR (100 MHz, CDCl_3_): 196.0, 167.3, 151.6, 140.2, 136.9, 136.6, 134.9, 132.9, 130.3, 130.1, 129.9, 129.8, 129.6, 129.2, 128.4, 128.3, 127.5, 123.2, 119.0, 115.3, 45.4, 42.6, 31.1, 28.9, 22.3. ESI (MS) *m*/*z*: 588.3 (*M* + H)^+^. HRMS (ESI-TOF) calculated for C_32_H_33_N_3_NaO_6_S (*M* + Na)^+^: 610.19823; found: 610.20065.

#### 3.1.12. Prop-2-ynylN-Cbz-*N*’-Boc-L-lysine (**27**)

To a stirred solution of **26** (3.81 g, 10.0 mmol) in anhydrous DMF (20 mL), K_2_CO_3_ (2.10 g, 15.19 mmol) was added at r.t.. After 0.5 h, 3-Bromopropyne (1.72 mL, 0.02 mol mmol, 80% in toluene) was slowly added dropwise. The reaction mixture was stirred at r.t. overnight and then filtered. The filtrate was diluted with water (40 mL) and extracted with EtOAc (40 mL × 3). The combined organic layer was washed with 1 N HCl aqueous (40 mL × 2), saturated NaHCO_3_ (40 mL × 2) and brine (40 mL × 2), dried over anhydrous Na_2_SO_4_, filtered, and evaporated. Purified by column chromatography on silica gel [petroleum ether-EtOAc (8:1)] to give product **27** (3.0 g, 72%) as a white solid. HPLC analysis: 96.5%. M.p. 119–121 °C. ^1^H-NMR (400 MHz, CDCl_3_): 1.37–1.48 (m, 13H), 1.70–1.91 (m, 2H), 2.51 (s, 1H), 2.98–3.16 (m, 2H), 4.37–4.42 (m, 1H), 4.62 (br s, 1H), 4.73 (ddd, *J* = 37.2, 15.6, 1.6 Hz, 2H), 5.10 (s, 2H), 5.49 (br d, *J* = 6.8 Hz, 1H), 7.29–7.36 (m, 5H). ^13^C-NMR (100 MHz, CDCl_3_): 171.7, 156.0, 155.9, 136.1, 128.4, 128.1, 128.0, 79.1, 75.4, 67.0, 53.6, 52.6, 39.8, 31.8, 29.5, 28.3, 22.1. ESI (MS) *m*/*z*: 419.2 (*M* + H)^+^. HRMS (ESI-TOF) calculated for C_22_H_30_N_2_NaO_6_ (*M* + Na)^+^: 441.19961; found: 441.20100.

#### 3.1.13. Prop-2-ynyl*N*-Cbz-*N*’-(5-{(3a*S*,4*S*,6a*R*)-2-oxohexahydro-1*H*-thieno[3,4-d]imidazol-4-yl}pentanoyl)-L-lysine (**28**)

To a solution of **27** (6.48 g, 15.50 mmol) in CH_2_Cl_2_ (20 mL), trifluoroacetic acid (TFA, 10 mL, 0.14 mol) was added, and the reaction mixture was stirred at 0 °C for 2 h. Then, the reaction mixture was concentrated in vacuo. The residue was redissolved in EtOAc (20 mL) and washed with saturated NaHCO_3_ (20 mL × 2), 1 M HCl aqueous (20 mL × 2), and brine (20 mL × 2), dried over Na_2_SO_4_, and concentrated to give crude Prop-2-ynyl *N*-Cbz-L-lysine as a colorless oil, which was used for next reaction without further purification. To a stirred solution of D-biotin (3.79 g, 15.50 mmol) and Et_3_N (2.57 mL, 18.60 mmol) in anhydrous DMF (20 mL), isobutyl chloroformate (2.12 g, 15.50 mmol) was slowly added at 0 °C under nitrogen. After 2 h, the reaction mixture was added successively to Prop-2-ynyl *N*-Cbz-L-lysine (5.50 g, 15.50 mmol) in anhydrous DMF (10 mL) and Et_3_N (3.10 g, 0.03 mol). The reaction mixture was stirred at r.t. overnight, and then poured into ice water (100 mL). The aqueous layer was extracted with EtOAc (50 mL × 3). The combined organic layer was washed with 1 N KHSO_4_ (50 mL × 2), 1 N NaHCO_3_ solution (50 mL × 2) and saturated brine (50 mL × 2), dried over anhydrous Na_2_SO_4_, filtered, and concentrated. The residue was purified by flash silica gel column chromatography [CH_2_Cl_2_-MeOH (8:1)] to give **28** as a white solid (6.50 g, 77%). HPLC analysis: 95.4%. M.p. 120–121 °C. ^1^H-NMR (400 MHz, CD_3_OD): 1.28–1.79 (m, 12H), 2.09 (t, *J* = 7.2 Hz, 2H), 2.59 (d, *J* = 12.8 Hz, 1H), 2.82 (dd, *J* = 12.8, 5.2 Hz, 1H), 2.86 (t, *J* = 2.4 Hz, 1H), 3.05–3.11 (m, 3H), 4.08–4.12 (m, 1H), 4.17–4.20 (m, 1H), 4.36–4.39 (m, 1H), 4.64 (ddd, *J* = 26.8, 15.6, 2.4 Hz, 2H), 5.00 (s, 2H), 7.18–7.26 (m, 5H), 7.85 (br t, *J* = 4.4 Hz, 1H). ^13^C-NMR (100 MHz, CD_3_OD): 176.0, 173.3, 166.1, 158.7, 138.2, 129.5, 129.0, 128.8, 78.5, 76.6, 67.7, 63.4, 61.6, 57.0, 55.4, 53.4, 41.0, 40.0, 36.8, 32.1, 29.9, 29.8, 29.5, 26.9, 24.2. ESI (MS) *m*/*z*: 545.3 (*M* + H)^+^. HRMS (ESI-TOF) calculated for C_27_H_36_N_4_NaO_6_S (*M* + Na)^+^: 567.22478; found: 567.22561.

#### 3.1.14. (*S*)-Prop-2-ynyl-2-[(*S*)-6-dansylamide-2-(4-benzoylbenzamido)hexanamido] -6-(5-{(3a*S*,4*S*,6a*R*)-2-oxohexahydro-1*H*-thieno[3,4-d]imidazol-4-yl}pentanamido)hexanoate (**30**)

Compound **28** (5.60 g, 10.28 mmol) was dissolved in a mixed solution of 30% HBr/HOAC (30.0 mL). The reaction mixture was stirred at r.t. for 1 h, and then concentrated in vacuo to give a colorless oil, which was almost a pure product and was used for the next reaction without further purification. The **29** (4.19 g, 10.20 mmol) was dissolved in anhydrous DMF (50 mL), to which O-(7-Azabenzotriazol-1-yl)-*N*,*N*,*N*’,*N*’-tetraMethyluronium hexafluorophosphate (HATU, 3.88 g, 10.21 mmol), DIPEA (5.05 mL, 30.56 mmol) was added at 0 °C. After the mixture was stirred for 5 min at r.t., **25** (6.00 g, 10.21 mmol) was added. The mixture was stirred at r.t. overnight. The mixture was diluted with water (100 mL) and extracted by EtOAc (100 mL × 2). The combined organic layer was washed with 1 N KHSO_4_ (100 mL × 2), saturated NaHCO_3_ solution (100 mL × 2), and saturated brine (100 mL × 2), dried over anhydrous Na_2_SO_4_, filtered, and concentrated. The residue was purified by preparative-reverse phase HPLC to give **30** (4.30 g, 43%) as a white solid. HPLC analysis: 95.1%. M.p. 105–108 °C. ^1^H-NMR (400 MHz, CDCl_3_): 1.43–1.88 (m, 18H), 2.05 (s, 2H), 2.49 (s, 1H), 2.71 (d, *J* = 12.8 Hz, 1H), 2.84 (s, 6H), 2.94 (s, 2H), 3.09 (s, 1H), 3.21 (s, 2H), 3.46 (s, 1H), 4.32 (s, 1H), 4.49–4.53 (m, 2H), 4.63–4.66 (m, 2H), 4.73–4.76 (m, 1H), 5.78 (s, 1H), 6.66–6.71 (m, 2H), 6.85 (s, 1H), 7.10 (d, *J* = 6.4 Hz, 1H), 7.40–7.49 (m, 4H),7.58–7.60 (m, 1H), 7.80 (dd, *J* = 17.6, 7.2 Hz, 4H), 7.91 (d, *J* = 5.2 Hz, 1H), 8.00 (d, *J* = 7.2 Hz, 2H), 8.18 (d, *J* = 6.0 Hz, 1H), 8.34 (d, *J* = 6.4 Hz, 2H), 8.48 (d, *J* = 8.0 Hz, 1H). ^13^C-NMR (100 MHz, CDCl_3_): 196.0, 173.7, 173.0, 171.6, 167.2, 164.3, 151.8, 140.3, 137.0, 136.8, 135.4, 132.9, 130.1, 129.9, 129.6, 129.1, 128.5, 128.1, 127.4, 123.2, 119.1, 115.1, 77.2, 75.6, 62.1, 60.2, 55.8, 54.2, 52.8, 52.3, 50.7, 45.4, 42.0, 40.6, 38.7, 35.3, 31.2, 30.7, 28.6, 28.5, 27.8, 25.3, 22.4, 22.2. ESI (MS) *m*/*z*: 980.0 (*M* + H)^+^. HRMS (ESI-TOF) calculated for C_51_H_61_N_7_NaO_9_S_2_ (*M* + Na)^+^: 1002.38644; found: 1002.38858.

#### 3.1.15. General Procedure for Synthesis of compounds **2a**–**2d**, **3a**–**3d**, and **31a**–**31d**

To a solution of **6** (0.32 mmol) and **20**, **21,** or **22** (0.32 mmol) in CH_2_Cl_2_ (1.5 mL) and H_2_O (1.5 mL), CuSO_4_·5H_2_O (0.39 mmol) and sodium ascorbate (0.51 mmol) were added. The resulting solution was stirred at r.t. overnight. The solvent was evaporated in vacuo, and then the residue was redissolved in EtOAc (10 mL), washed with water (10 mL × 3), and dried over anhydrous Na_2_SO_4_. The organic layer was evaporated, and the residue was purified by preparative-reverse phase HPLC to give target products **2a**–**2d**, **3a**–**3d**, and **31a**–**31d** as white solids.

*Isopropyl 1-(2-{2-[4-({[(S)-2-(4-benzoylbenzamido)-6-(5-{(3aS,4S,6aR)-2-oxohexahydro-1H-thieno[3,4-d]imidazol-4-yl}pentanamido)hexanoyl]oxy}methyl)-1H-1,2,3-triazol-1-yl]ethoxy}-2-oxoethyl)-4-(2-chlorophenyl)-2-methyl-5-oxo-1,4,5,7-tetrahydrofuro[3,4-b]pyridine-3-carboxylate* (**2a**). HPLC analysis: 98.5%. M.p. 128–130 °C. ^1^H-NMR (400 MHz, *d*_6_-DMSO): 0.76 (d, *J* = 6.4 Hz, 3H), 1.11 (d, *J* = 6.4 Hz, 3H), 1.23–1.48 (m, 10H), 1.79–1.84 (m, 2H), 2.03 (t, *J* = 6.4 Hz, 2H), 2.23 (s, 3H), 2.55 (d, *J* = 12.8 Hz, 1H), 2.80 (dd, *J* = 12.0, 4.4 Hz, 1H), 3.00–3.08 (m, 3H), 4.08–4.11 (m, 1H), 4.26–4.30 (m, 1H), 4.38–4.50 (m, 3H), 4.59–4.63 (m, 2H), 4.72–4.92 (m, 5H), 5.18 (s, 3H), 6.34 (br s, 1H), 6.40(br s, 1H), 7.17 (t, *J* = 6.4 Hz, 1H), 7.26 (t, *J* = 7.2 Hz, 1H), 7.31 (d, *J* = 7.6 Hz, 1H), 7.35 (d, *J* = 7.6 Hz, 1H), 7.58 (t, *J* = 7.6 Hz, 2H), 7.71 (t, *J* = 7.2 Hz, 1H), 7.76 (d, *J* = 7.2 Hz, 3H), 7.81 (d, *J* = 8.4 Hz, 2H), 8.03 (d, *J* = 8.0 Hz, 2H), 8.22 (s, 1H), 8.94 (d, *J* = 7.2 Hz, 1H). ^13^C-NMR (100 MHz, *d*_6_-DMSO): 195.8, 172.4, 172.3, 171.3, 168.9, 166.7, 166.5, 163.2, 158.5, 146.1, 143.3, 142.3, 139.9, 137.4, 137.1, 133.5, 132.5, 131.3, 130.2, 129.9, 129.2, 129.1,128.5, 128.2, 127.8, 125.8, 107.9, 101.7, 67.4, 65.9,64.1, 61.5, 59.7, 58.2, 55.9, 53.4, 48.9, 47.6, 38.5, 35.7, 34.6, 30.4, 29.2, 28.7, 28.5, 25.8, 23.6, 21.8, 21.1, 15.2. ESI (MS) *m*/*z*: 1093.4 (*M* + H)^+^. HRMS (ESI-TOF) calculated for C_55_H_61_ClN_8_NaO_12_S (*M* + Na)^+^: 1115.3716; found 1115.3710.

*Isopropyl 1-[2-(2-{2-[4-({[(S)-2-(4-benzoylbenzamido)-6-(5-{(3aS,4S,6aR)-2-oxohexahydro-1H-thieno[3,4-d]imidazol-4-yl}pentanamido)hexanoyl]oxy}methyl)-1H-1,2,3-triazol-1-yl]ethoxy}ethoxy)-2-oxoethyl]-4-(2-chlorophenyl)-2-methyl-5-oxo-1,4,5,7-tetrahydrofuro[3,4-b]pyridine-3-carboxylate* (**2b**). HPLC analysis: 98.5%. M.p. 103–105 °C. ^1^H-NMR (400 MHz, *d*_6_-DMSO): 0.76 (d, *J* = 6.4 Hz, 3H), 1.10 (d, *J* = 6.4 Hz, 3H), 1.23–1.63 (m, 10H), 1.77–1.83 (m, 2H), 2.03 (t, *J* = 7.2 Hz, 2H), 2.28 (s, 3H), 2.55 (d, *J* = 10.4 Hz, 1H), 2.79 (dd, *J* = 15.2, 8.0 Hz, 1H), 3.00–3.08 (m, 3H), 3.66 (t, *J* = 5.2 Hz, 2H), 3.84 (t, *J* = 5.2 Hz, 2H), 4.08–4.11 (m, 1H), 4.29 (t, *J* = 4.0 Hz, 3H), 4.40–4.61 (m, 5H), 4.70–4.77 (m, 1H), 4.89 (dd, *J* = 36.4, 16.4 Hz, 2H), 5.20 (s, 1H), 5.21 (s, 2H), 6.36 (br s, 1H), 6.42 (br s, 1H), 7.17 (td, *J* = 7.6, 1.6 Hz, 1H), 7.27 (td, *J* = 7.2, 0.8 Hz, 1H), 7.32 (dd, *J* = 8.0, 1.2 Hz, 1H), 7.40 (d, *J* = 7.2 Hz, 1H), 7.58 (t, *J* = 8.0 Hz, 2H), 7.71 (tt, *J* = 7.2, 1.2 Hz, 1H), 7.76 (d, *J* = 7.2 Hz, 3H), 7.82 (d, *J* = 8.4 Hz, 2H), 8.03 (d, *J* = 8.4 Hz, 2H), 8.13 (s, 1H), 8.95 (d, *J* = 7.2 Hz, 1H). ^13^C-NMR (100 MHz, *d*_6_-DMSO): 195.3, 171.9, 171.8, 170.8, 168.7, 166.2, 166.1, 162.7, 158.2, 145.6, 142.8, 141.6, 139.4, 137.0, 136.6, 133.0, 132.0, 130.9, 129.7, 129.4, 128.7, 128.6, 128.0, 127.6, 127.3, 125.0, 107.5, 101.2, 68.5, 67.9, 66.9, 65.4, 64.4, 61.0, 59.2, 57.8, 55.4, 52.9, 49.3, 47.3, 38.0, 35.2, 34.2, 30.0, 28.7, 28.2, 28.0, 25.3, 23.1, 21.3, 20.6, 14.7. ESI (MS) *m*/*z*: 1137.4 (*M* + H)^+^. HRMS (ESI-TOF) calculated for C_57_H_65_ClN_8_NaO_13_S (*M* + Na)^+^: 1159.3978; found: 1159.3973.

*Isopropyl 1-{2-[2-(2-{2-[4-({[(S)-2-(4-benzoylbenzamido)-6-(5-{(3aS, 4S,6aR)-2-oxohexahydro-1H-thieno[3,4-d]imidazol-4-yl}pentanamido)hexanoyl]oxy}methyl)-1H-1,2,3-triazol-1-yl]ethoxy}ethoxy)ethoxy]-2-oxoethyl}-4-(2-chlorophenyl)-2-methyl-5-oxo-1,4,5,7-tetrahydrofuro[3,4-b]pyridine-3-carboxylate* (**2c**). HPLC analysis: 95.2%. M.p. 77–79 °C. ^1^H-NMR (400 MHz, *d*_6_-DMSO): 0.76 (d, *J* = 6.4 Hz, 3H), 1.11 (d, *J* = 6.4 Hz, 3H), 1.23–1.63 (m, 10H), 1.78–1.83 (m, 2H), 2.03 (t, *J* = 7.2 Hz, 2H), 2.30 (s, 3H), 2.55 (d, *J* = 12.4 Hz, 1H), 2.79 (dd, *J* = 12.4, 5.2 Hz, 1H), 2.98–3.08 (m, 3H), 3.49 (s, 4H), 3.61 (t, *J* = 4.8 Hz, 2H), 3.79 (t, *J* = 7.2 Hz, 2H), 4.08–4.11 (m, 1H), 4.27–4.30 (m, 3H), 4.41–4.61 (m, 5H), 4.71–4.77 (m, 1H), 4.89 (dd, *J* = 33.6, 16.4 Hz, 2H), 5.20 (s, 3H), 6.34 (br s, 1H), 6.40 (br s, 1H), 7.17 (td, *J* = 7.6, 1.6 Hz, 1H), 7.27 (t, *J* = 7.6 Hz, 1H), 7.32 (d, *J* = 7.6 Hz, 1H), 7.40 (d, *J* = 7.6 Hz, 1H), 7.58 (t, *J* = 7.6 Hz, 2H), 7.73 (t, *J* = 8.0 Hz, 1H), 7.76 (d, *J* = 7.2 Hz, 3H), 7.82 (d, *J* = 8.4 Hz, 2H), 8.03 (d, *J* = 8.0 Hz, 2H), 8.12 (s, 1H), 8.94 (d, *J* = 7.2 Hz, 1H). ^13^C-NMR (100 MHz, *d*_6_-DMSO): 195.4, 171.9, 171.9, 170.8, 168.7, 166.2, 166.0, 162.7, 158.2, 145.6, 142.8, 141.6, 139.4, 137.0, 136.6, 133.0, 132.0, 130.9, 129.7, 129.4, 128.7, 128.6, 128.0, 127.7, 127.3, 125.0, 107.5, 101.1, 69.5, 68.6, 68.1, 66.9, 65.4, 64.5, 61.0, 59.2, 57.8, 55.4, 52.9, 49.4, 47.4, 38.0, 35.2, 34.2, 29.9, 28.7, 28.2, 28.0, 25.3, 23.1, 21.3, 20.7, 14.7. ESI (MS) *m*/*z*: 1181.5 (M+H)^+^. HRMS (ESI-TOF) calculated for C_59_H_69_ClN_8_NaO_14_S (*M* + Na)^+^: 1203.4240; found: 1203.4235.

*Isopropyl 1-{14-[4-({[(S)-2-(4-benzoylbenzamido)-6-(5-{(3aS,4S,6aR)-2-oxohexahydro-1H-thieno[3,4-d]imidazol-4-yl}pentanamido)hexanoyl]oxy}methyl)-1H-1,2,3-triazol-1-yl]-2-oxo-3,6,9,12-tetraoxatetradecyl}-4-(2-chlorophenyl)-2-methyl-5-oxo-1,4,5,7-tetrahydrofuro[3,4-b]pyridine-3-carboxylate* (**2d**). HPLC analysis: 97.4%. M.p. 61–63 °C. ^1^H-NMR (400 MHz, *d*_6_-DMSO): 0.77 (d, *J* = 6.0 Hz, 3H), 1.11 (d, *J* = 6.0 Hz, 3H), 1.38–1.56 (m, 10H), 1.78–1.82 (m, 2H), 2.03 (t, *J* = 7.2 Hz, 2H), 2.30 (s, 3H), 2.55 (d, *J* = 9.2 Hz, 1H), 2.80 (dd, *J* = 12.4, 5.2 Hz, 1H), 2.99–3.08 (m, 3H), 3.44–3.52 (m, 8H), 3.64 (t, *J* = 4.0 Hz, 2H), 3.80 (t, *J* = 5.2 Hz, 2H), 4.08–4.11 (m, 1H), 4.27–4.32 (m, 3H), 4.40–4.61 (m, 5H), 4.71–4.77 (m, 1H), 4.85 (dd, *J* = 32.0, 15.2 Hz, 2H), 5.20 (s, 2H), 5.21 (s, 1H), 6.40 (br s, 2H), 7.17 (t, *J* = 8.4 Hz, 1H), 7.27 (t, *J* = 8.4 Hz, 1H), 7.32 (d, *J* = 8.0 Hz, 1H), 7.40 (d, *J* = 8.0 Hz, 1H), 7.58 (t, *J* = 7.6 Hz, 2H), 7.71 (t, *J* = 7.2 Hz, 1H), 7.76 (d, *J* = 7.2 Hz, 3H), 7.82 (d, *J* = 8.0 Hz, 2H), 8.03 (d, *J* = 8.0 Hz, 2H), 8.12 (s, 1H), 8.94 (d, *J* = 7.2 Hz, 1H). ^13^C-NMR (100 MHz, *d*_6_-DMSO): 195.4, 171.9, 171.8, 170.8, 168.7, 166.2, 166.0, 162.7, 158.2, 145.7, 142.8, 141.5, 139.4, 137.0, 136.6, 133.0, 132.0, 130.9, 129.7, 129.4, 128.7, 128.6, 128.0, 127.7, 127.3, 125.1, 107.4, 101.1, 69.7, 69.6, 69.5, 69.4, 68.6, 68.1, 66.9, 65.4, 64.5, 61.0, 59.2, 57.8, 55.4, 52.9, 49.4, 47.3, 38.0, 35.2, 34.2, 30.0, 28.7, 28.2, 28.0, 25.3, 23.1, 21.3, 20.6, 14.7. ESI (MS) *m*/*z*: 1225.5 (*M* + H)^+^. HRMS (ESI-TOF) calculated for C_61_H_73_ClN_8_NaO_15_S (M+Na)^+^: 1247.4502; found: 1247.4497.

*Isopropyl 1-(2-{2-[4-({[(S)-6-dansylamide-2-(4-benzoylbenzamido)hexanoyl]oxy}methyl)-1H-1,2,3-triazol-1-yl]ethoxy}-2-oxoethyl)-4-(2-chlorophenyl)-2-methyl-5-oxo-1,4,5,7-tetrahydrofuro[3,4-b]pyridine-3-carboxylate* (**3a**). HPLC analysis: 91.8%. M.p. 119–121 °C. ^1^H-NMR (400 MHz, *d*_6_-DMSO): 0.75 (d, *J* = 6.4 Hz, 3H), 1.10 (d, *J* = 6.4 Hz, 3H), 1.22–1.34 (m, 4H), 1.63–1.69 (m, 2H), 2.22 (s, 3H), 2.73–2.79 (m, 2H), 2.82 (s, 6H), 4.29–4.54 (m, 3H), 4.59–4.61 (m, 2H), 4.70–4.92 (m, 5H), 5.15 (d, *J* = 3.6 Hz, 2H), 5.18 (s, 1H), 7.16 (tt, *J* = 8.0, 1.6 Hz, 1H), 7.25 (t, *J* = 6.8 Hz, 2H), 7.33 (dd, *J* = 8.0, 14.8 Hz, 2H), 7.55–7.62 (m, 4H), 7.71 (t, *J* = 7.2 Hz, 1H), 7.76 (d, *J* = 8.0 Hz, 2H), 7.80 (d, *J* = 8.4 Hz, 2H), 7.89 (t, *J* = 7.6 Hz, 1H), 7.99 (d, *J* = 8.0 Hz, 2H), 8.08 (d, *J* = 8.0 Hz, 1H), 8.20 (s, 1H), 8.29 (d, *J* = 8.8 Hz, 1H), 8.44 (d, *J* = 8.0 Hz, 1H), 8.87 (d, *J* = 7.2 Hz, 1H). ^13^C-NMR (100 MHz, *d*_6_-DMSO): 195.3, 171.7, 170.7, 168.4, 166.1, 166.0, 158.0, 150.7, 145.5, 142.7, 141.7, 139.3, 136.9, 136.6, 136.1, 133.0, 132.0, 130.8, 129.6, 129.4, 129.1, 129.0, 128.8, 128.7, 128.6, 128.1, 128.0, 127.7, 127.6, 127.2, 125.2, 123.6, 119.4, 115.3, 107.4, 101.1, 66.8, 65.3, 63.5, 57.6, 52.7, 48.4, 47.0, 45.0, 42.1, 34.1, 29.7, 28.7, 22.7, 21.3, 20.6, 14.6. ESI (MS) *m*/*z*: 1100.5 (*M* + H)^+^. HRMS (ESI-TOF) calculated for C_57_H_5__9_ClN_7_O_12_S (*M* + H)^+^: 1100.3631; found: 1100.3625.

*Isopropyl 1-[2-(2-{2-[4-({[(S)-6-dansylamide-2-(4-benzoylbenzamido)hexanoyl]oxy}methyl)-1H-1,2,3-triazol-1-yl]ethoxy}ethoxy)-2-oxoethyl]-4-(2-chlorophenyl)-2-methyl-5-oxo-1,4,5,7-tetrahydrofuro[3,4-b]pyridine-3-carboxylate* (**3b**). HPLC analysis: 96.6%. M.p. 124–126 °C. ^1^H-NMR (400 MHz, *d*_6_-DMSO): 0.76 (d, *J* = 6.0 Hz, 3H), 1.10 (d, *J* = 6.4 Hz, 3H), 1.23–1.32 (m, 4H), 1.62–1.68 (m, 2H), 2.27 (s, 3H), 2.73–2.78 (m, 2H), 2.82 (s, 6H), 3.65 (t, *J* = 5.2 Hz, 2H), 3.83 (t, *J* = 5.2 Hz, 2H), 4.26–4.32 (m, 3H), 4.46–4.59 (m, 4H), 4.70–4.76 (m, 1H), 4.88 (dd, *J* = 36.4, 16.8 Hz, 2H), 5.18 (s, 2H), 5.20 (s, 1H), 7.16 (td, *J* = 7.6, 1.2 Hz, 1H), 7.23–7.27 (m, 2H), 7.31 (d, *J* = 8.0 Hz, 1H), 7.40 (d, *J* = 8.0 Hz, 1H), 7.54–7.62 (m, 4H), 7.71 (t, *J* = 7.2 Hz, 1H), 7.76 (d, *J* = 7.2 Hz, 2H), 7.80 (d, *J* = 8.4 Hz, 2H), 7.88 (t, *J* = 6.0 Hz, 1H), 7.99 (d, *J* = 8.0 Hz, 2H), 8.08 (d, *J* = 11.2 Hz, 1H), 8.10 (s, 1H), 8.29 (d, *J* = 9.2 Hz, 1H), 8.44 (d, *J* = 8.4 Hz, 1H), 8.86 (d, *J* = 6.8 Hz, 1H). ^13^C-NMR (100 MHz, *d*_6_-DMSO): 195.4, 171.8, 170.8, 168.7, 166.2, 166.0, 158.2, 150.9, 145.6, 142.8, 141.6, 139.4, 136.9, 136.6, 136.2, 133.0, 132.0, 130.9, 129.7, 129.4, 129.2, 129.1, 128.9, 128.7, 128.6, 128.2, 128.0, 127.7, 127.6, 127.3, 125.0, 123.6, 119.4, 115.2, 107.5, 101.1, 68.5, 67.8, 66.9, 65.4, 64.4, 57.7, 52.8, 49.3, 47.3, 45.1, 42.2, 34.2, 29.8, 28.8, 22.7, 21.3, 20.6, 14.7. ESI (MS) *m*/*z*: 1144.5 (*M* + H)^+^. HRMS (ESI-TOF) calculated for C_59_H_6__3_ClN_7_O_13_S (*M* + H)^+^: 1144.3893; found: 1144.3888.

*Isopropyl 1-{2-[2-(2-{2-[4-({[(S)-6-dansylamide-2-(4-benzoylbenzamido)hexanoyl]oxy}methyl)-1H-1,2,3-triazol-1-yl]ethoxy}ethoxy)ethoxy]-2-oxoethyl}-4-(2-chlorophenyl)-2-methyl-5-oxo-1,4,5,7-tetrahydrofuro[3,4-b]pyridine-3-carboxylate* (**3c**). HPLC analysis: 97.8%. M.p. 96–98 °C. ^1^H-NMR (400 MHz, *d*_6_-DMSO): 0.76 (d, *J* = 7.2 Hz, 3H), 1.10 (d, *J* = 6.4 Hz, 3H), 1.23–1.37 (m, 4H), 1.62–1.67 (m, 2H), 2.29 (s, 3H), 2.73–2.78 (m, 2H), 2.82 (s, 6H), 3.48 (s, 4H), 3.59 (t, *J* = 5.6 Hz, 2H), 3.78 (t, *J* = 5.2 Hz, 2H), 4.27–4.33 (m, 3H), 4.48–4.61 (m, 4H), 4.70–4.76 (m, 1H), 4.89 (dd, *J* = 34.0, 16.4 Hz, 2H), 5.17 (s, 2H), 5.20 (s, 1H), 7.16 (td, *J* = 7.6, 1.2 Hz, 1H), 7.23–7.28 (m, 2H), 7.31 (d, *J* = 7.6 Hz, 1H), 7.40 (d, *J* = 8.0 Hz, 1H), 7.54–7.62 (m, 4H), 7.71 (t, *J* = 7.6 Hz, 1H), 7.76 (d, *J* = 8.0 Hz, 2H), 7.80 (d, *J* = 8.4 Hz, 2H), 7.88 (t, *J* = 4.8 Hz, 1H), 7.99 (d, *J* = 8.4 Hz, 2H), 8.08 (d, *J* = 7.2 Hz, 1H), 8.10 (s, 1H), 8.29 (d, *J* = 8.8 Hz, 1H), 8.44 (d, *J* = 9.2 Hz, 1H), 8.86 (d, *J* = 7.6 Hz, 1H). ^13^C-NMR (100 MHz, *d*_6_-DMSO): 195.4, 171.8, 170.8, 168.7, 166.2, 166.0, 158.2, 150.6, 145.6, 142.8, 141.5, 139.4, 136.9, 136.6, 136.2, 133.0, 132.0, 130.9, 129.7, 129.4, 129.1, 129.0, 128.9, 128.7, 128.6, 128.2, 128.0, 127.7, 127.6, 127.3, 125.0, 123.7, 119.5, 115.3, 107.5, 101.1, 69.5, 68.6, 68.1, 66.9, 65.4, 64.5, 57.7, 52.8, 49.4, 47.3, 45.1, 42.2, 34.2, 29.8, 28.8, 22.7, 21.3, 20.6, 14.7. ESI (MS) *m*/*z*: 1188.5 (*M* + H)^+^. HRMS (ESI-TOF) calculated for C_61_H_6__7_ClN_7_O_14_S (*M* + H)^+^: 1188.4155; found: 1188.4150.

*Isopropyl 1-{14-[4-({[(S)-6-dansylamide-2-(4-benzoylbenzamido)hexanoyl]oxy}methyl)-1H-1,2,3-triazol-1-yl]-2-oxo-3,6,9,12-tetraoxatetradecyl}-4-(2-chlorophenyl)-2-methyl-5-oxo-1,4,5,7-tetrahydrofuro[3,4-b]pyridine-3-carboxylate* (**3d**). HPLC analysis: 96.9%. M.p. 84–86 °C. ^1^H-NMR (400 MHz, *d*_6_-DMSO): 0.76 (d, *J* = 6.0 Hz, 3H), 1.10 (d, *J* = 6.0 Hz, 3H), 1.23–1.37 (m, 4H), 1.61–1.67 (m, 2H), 2.29 (s, 3H), 2.72–2.78 (m, 2H), 2.82 (s, 6H), 3.42–3.51 (m, 8H), 3.63 (t, *J* = 6.0 Hz, 2H), 3.79 (t, *J* = 5.2 Hz, 2H), 4.28–4.33 (m, 3H), 4.49–4.61 (m, 4H), 4.70–4.77 (m, 1H), 4.89 (dd, *J* = 32.8, 16.4 Hz, 2H), 5.17 (s, 2H), 5.21 (s, 1H), 7.16 (td, *J* = 9.2, 1.6 Hz, 1H), 7.25 (t, *J* = 7.6 Hz, 2H), 7.30 (t, *J* = 8.0 Hz, 1H), 7.39 (d, *J* = 7.6 Hz, 1H), 7.54–7.62 (m, 4H), 7.71 (t, *J* = 7.6 Hz, 1H), 7.76 (d, *J* = 6.8 Hz, 2H), 7.80 (d, *J* = 8.4 Hz, 2H), 7.88 (t, *J* = 5.2 Hz, 1H), 7.99 (d, *J* = 8.0 Hz, 2H), 8.07 (d, *J* = 6.8 Hz, 1H), 8.10 (s, 1H), 8.29 (d, *J* = 8.4 Hz, 1H), 8.44 (d, *J* = 8.8 Hz, 1H), 8.86 (d, *J* = 6.8 Hz, 1H). ^13^C-NMR (100 MHz, *d*_6_-DMSO): 195.4, 171.8, 170.8, 168.7, 166.2, 166.0, 158.2, 150.8, 145.6, 142.8, 141.5, 139.4, 136.9, 136.6, 136.2, 133.0, 132.0, 130.9, 129.7, 129.4, 129.1, 129.0, 128.9, 128.7, 128.6, 128.2, 128.0, 127.7, 127.6, 127.3, 125.0, 123.6, 119.4, 115.3, 107.4, 101.1, 69.7, 69.6, 69.5, 69.4, 68.6, 68.1, 66.9, 65.4, 64.5, 57.7, 52.8, 49.4, 47.3, 45.1, 42.2, 34.2, 29.8, 28.8, 22.7, 21.3, 20.7, 14.7. ESI (MS) *m*/*z*: 1232.5 (*M* + H)^+^. HRMS (ESI-TOF) calculated for C_63_H_70_ClN_7_NaO_15_S (*M* + Na)^+^: 1254.4237; found: 1254.4231.

*Isopropyl 1-(2-{2-[4-({[(S)-6-(2-chloroacetamido)-2-(4-benzoylbenzamido)hexanoyl]oxy}methyl)-1H-1,2,3-triazol-1-yl]ethoxy}-2-oxoethyl)-4-(2-chlorophenyl)-2-methyl-5-oxo-1,4,5,7-tetrahydrofuro[3,4-b]pyridine-3-carboxylate* (**31a**). HPLC analysis: 90.9%. M.p. 89–91 °C. ^1^H-NMR (400 MHz, CDCl_3_): 0.81 (d, *J* = 6.4 Hz, 3H), 1.16 (dd, *J* = 6.0, 1.6 Hz, 3H), 1.38–1.60 (m, 4H), 1.82–1.99 (m, 2H), 2.32 (s, 3H), 3.24–3.33 (m, 2H), 3.93–4.02 (m, 2H), 4.11–4.29 (m, 2H), 4.61–4.69 (m, 7H), 4.80–4.87 (m, 1H), 5.29–5.34 (m, 2H), 5.38 (s, 1H), 6.80–6.83 (m, 1H), 7.06 (tt, *J* = 7.6, 1.6 Hz, 1H), 7.15 (t, *J* = 6.4 Hz, 1H), 7.24–7.34 (m, 3H), 7.50 (t, *J* = 7.6 Hz, 2H), 7.62 (t, *J* = 7.6 Hz, 1H), 7.77–7.83 (m, 5H), 7.95 (dd, *J* = 8.4, 2.8 Hz, 2H). ^13^C-NMR (100 MHz, CDCl_3_): 196.0, 172.1, 171.4, 167.8, 166.8, 166.7, 166.6, 156.7, 144.4, 141.9, 140.4, 137.0, 136.8, 133.3, 133.0, 131.1, 130.1, 130.0, 129.4, 128.5, 128.0, 127.3, 127.0, 124.4, 109.4, 103.3, 68.0, 65.2, 63.6, 58.4, 53.0, 48.8, 47.1, 42.7, 38.9, 35.1, 30.8, 28.8, 22.5, 21.6, 20.9, 15.2. ESI (MS) *m*/*z*: 945.1 (*M* + H)^+^. HRMS (ESI-TOF) calculated for C_47_H_49_Cl_2_N_6_O_11_ (*M* + H)^+^: 943.28364; found: 943.2932.

*Isopropyl 1-[2-(2-{2-[4-({[(S)-6-(2-chloroacetamido)-2-(4-benzoylbenzamido)hexanoyl]oxy}methyl)-1H-1,2,3-triazol-1-yl]ethoxy}ethoxy)-2-oxoethyl]-4-(2-chlorophenyl)-2-methyl-5-oxo-1,4,5,7-tetrahydrofuro[3,4-b]pyridine-3-carboxylate* (**31b**). HPLC analysis: 93.5%. M.p. 64–66 °C. ^1^H-NMR (400 MHz, CDCl_3_): 0.81 (d, *J* = 6.0 Hz, 3H), 1.18 (d, *J* = 6.4 Hz, 3H), 1.41–1.67 (m, 4H), 1.88–1.97 (m, 2H), 2.38 (s, 3H), 3.25–3.32 (m, 2H), 3.68 (t, *J* = 4.0 Hz, 2H), 3.88 (t, *J* = 5.2 Hz, 2H), 3.94–4.03 (m,2H), 4.20–4.37 (m, 4H), 4.51 (t, *J* = 5.2 Hz, 2H), 4.72–4.76 (m,3H), 4.83–4.88 (m, 1H), 5.32 (s, 2H), 5.42 (s, 1H), 6.72–6.78 (m, 1H), 7.06–7.18 (m, 3H), 7.25–7.29 (m, 1H), 7.37 (dt, *J* = 7.6, 1.2 Hz, 1H), 7.50 (t, *J* = 7.6 Hz, 2H), 7.62 (t, *J* = 7.6 Hz, 1H), 7.74 (s, 1H), 7.82 (dd, *J* = 17.2, 8.8 Hz, 4H), 7.95 (t, *J* = 8.4 Hz, 2H). ^13^C-NMR (100 MHz, CDCl_3_): 195.0,171.1, 170.5, 167.2, 165.8, 165.7, 165.6, 156.2, 143.7,141.2, 139.3, 135.9, 135.8, 132.2, 132.0, 130.1, 129.1, 129.0, 128.2, 127.5, 126.9, 126.3, 126.1, 108.3, 102.1, 68.1, 67.5, 66.9,64.4, 63.7, 57.5, 52.0, 49.1, 46.4, 41.7, 38.0, 33.9, 30.0, 27.7, 21.5, 20.6, 19.9, 14.2. ESI (MS) *m*/*z*: 987.1 (*M* + H)^+^. HRMS (ESI-TOF) calculated for C_49_H_52_Cl_2_N_6_NaO_12_ (*M* + Na)^+^: 1009.29125; found: 1009.29266.

*Isopropyl 1-{2-[2-(2-{2-[4-({[(S)-6-(2-chloroacetamido)-2-(4-benzoylbenzamido)hexanoyl]oxy}methyl)-1H-1,2,3-triazol-1-yl]ethoxy}ethoxy)ethoxy]-2-oxoethyl}-4-(2-chlorophenyl)-2-methyl-5-oxo-1,4,5,7-tetrahydrofuro[3,4-b]pyridine-3-carboxylate* (**31c**). HPLC analysis: 91.1%. M.p. 51–52 °C. ^1^H-NMR (400 MHz, CDCl_3_): 0.82 (d, *J* = 6.4 Hz, 3H), 1.18 (d, *J* = 6.0 Hz, 3H), 1.41–1.59 (m, 4H), 1.83–1.99 (m, 2H), 2.39 (s, 3H), 3.21–3.34 (m, 2H), 3.55 (s, 4H), 3.65(t, *J* = 4.8 Hz, 2H), 3.84 (t, *J* = 4.8 Hz, 2H), 3.95–4.04 (m, 2H), 4.19–4.32 (m, 2H), 4.37–4.39 (m, 2H), 4.51 (t, *J* = 4.8 Hz, 2H), 4.73 (s, 2H), 4.74–4.78 (m, 1H), 4.82–4.88 (m, 1H), 5.32 (s, 2H), 5.42 (s, 1H), 6.78 (t, *J* = 7.2 Hz, 1H), 7.06–7.16 (m, 3H), 7.25–7.28 (m, 1H), 7.38 (dd, *J* = 7.6, 1.6 Hz, 1H), 7.50 (t, *J* = 8.0 Hz, 2H), 7.62 (t, *J* = 7.2 Hz, 1H), 7.79 (s, 1H), 7.83 (t, *J* = 8.0 Hz, 4H), 7.95 (t, *J* = 8.8 Hz, 2H). ^13^C-NMR (100 MHz, CDCl_3_): 195.9, 172.1, 171.3, 168.0, 166.8, 166.6, 166.4, 156.9, 144.7, 142.1, 140.4, 137.0, 136.9, 133.2, 132.9, 131.1, 130.1, 130.0, 129.3, 128.5, 127.9, 127.3, 127.0, 125.0, 109.3, 103.3, 70.5, 70.4, 69.3, 68.8, 67.9, 65.4, 64.9, 58.6, 52.8, 50.3, 47.5, 42.7, 39.1, 34.9, 31.3, 28.7, 22.5, 21.6, 20.9, 15.2. ESI (MS) *m*/*z*: 1031.1 (*M* + H)^+^. HRMS (ESI-TOF) calculated for C_51_H_56_Cl_2_N_6_NaO_13_ (*M* + Na)^+^: 1053.31746; found: 1053.31881.

*Isopropyl 1-{14-[4-({[(S)-6-(2-chloroacetamido)-2-(4-benzoylbenzamido)hexanoyl]oxy}methyl)-1H-1,2,3-triazol-1-yl]-2-oxo-3,6,9,12-tetraoxatetradecyl}-4-(2-chlorophenyl)-2-methyl-5-oxo-1,4,5,7-tetrahydrofuro[3,4-b]pyridine-3-carboxylate* (**31d**). HPLC analysis: 90.6%. M.p. 51–52 °C. ^1^H-NMR (400 MHz, CDCl_3_): 0.82(d, *J* = 6.0 Hz, 3H), 1.18(d, *J* = 6.4 Hz, 3H), 1.42–1.58(m, 4H), 1.82–2.01 (m, 2H), 2.39(s, 3H), 3.24–3.33(m, 2H), 3.54–3.61 (m, 8H), 3.71 (t, *J* = 4.4 Hz, 2H), 3.86(t, *J* = 4.8 Hz, 2H), 3.95–4.04 (m, 2H), 4.19 (d, *J* = 18.4 Hz, 1H), 4.31 (dd, *J* = 14.4, 7.6 Hz, 1H), 4.39–4.41 (m, 2H), 4.53 (t, *J* = 4.8 Hz, 2H), 4.71 (s, 2H), 4.74–4.79(m, 1H), 4.82–4.88(m, 1H), 5.27–5.35(m, 2H), 5.42(s, 1H), 6.78 (t, *J* = 5.6 Hz, 1H), 7.07–7.10 (m, 2H), 7.15–7.19 (m, 1H), 7.26–7.28 (m, 1H), 7.39(d, *J* = 8.0 Hz, 1H), 7.50 (t, *J* = 8.0 Hz, 2H), 7.62(t, *J* = 7.2 Hz, 1H), 7.80 (d, *J* = 7.2 Hz, 2H), 7.82–7.85 (m, 3H), 7.94–7.97 (m, 2H). ^13^C-NMR (100 MHz, CDCl_3_): 195.0,171.1, 170.4, 167.1, 165.8, 165.6, 165.5, 156.0, 143.8,141.2, 141.1, 139.3, 135.98, 135.96, 132.2, 131.9, 130.1, 129.1, 129.0, 128.3, 127.5, 126.9, 126.3, 126.1, 124.1, 108.2, 102.2, 69.4, 68.3, 67.7, 66.8, 64.3, 63.9, 57.5, 51.8, 49.3, 46.4, 41.7, 38.1, 34.0, 30.2, 27.7, 21.5, 20.6, 19.9, 14.2. ESI (MS) *m*/*z*: 1075.1 (*M* + H)^+^. HRMS (ESI-TOF) calculated for C_53_H_60_Cl_2_N_6_NaO_14_ (*M* + Na)^+^: 1097.34368; found: 1097.34555.

#### 3.1.16. General Procedure for Synthesis of compounds **4a**–**4d**

To a solution of **6a**–**6d** (0.32 mmol) and **30** (0.31 g, 0.32 mmol) in CH_2_Cl_2_ (1.5 mL) and H_2_O (1.5 mL), CuSO_4_·5H_2_O (97.37 mg, 0.39 mmol) and sodium ascorbate (0.10 g, 0.51 mmol) were added. The resulting solution was stirred at r.t. overnight. The solvent was evaporated in vacuo, and then the residue was redissolved in EtOAc (10 mL), washed by water (10 mL × 2), and dried over anhydrous Na_2_SO_4_. The combined organic layer was evaporated, and the residue was purified by preparative-reverse phase HPLC to give the target products of **4a**–**4d** as pale yellow solids.

*Isopropyl 1-(2-{2-[4-({[(2-[(S)-6-dansylamide-2-(4-benzoylbenzamido)hexanamido] -6-(5-{(3aS,4S,6aR)-2-oxohexahydro-1H-thieno[3,4-d]imidazol-4-yl}pentanamido) hexanoyl]oxy}methyl)-1H-1,2,3-triazol-1-yl]ethoxy}-2-oxoethyl)-4-(2-chlorophenyl)-2-methyl-5-oxo-1,4,5,7-tetrahydrofuro[3,4-b]pyridine-3-carboxylate* (**4a**). HPLC analysis: 96.6%. M.p. 93–95 °C. ^1^H-NMR (400 MHz, *d*_6_-DMSO): 0.76 (d, *J* = 6.4 Hz, 3H), 1.11 (d, *J* = 6.0 Hz, 3H), 1.28–1.61 (m, 18H), 2.03 (t, *J* = 7.6 Hz, 2H), 2.24 (s, 3H), 2.55 (d, *J* = 12.4 Hz, 1H), 2.74–2.80 (m, 3H), 2.82 (s, 6H), 2.95–2.99 (m, 2H), 3.04–3.09 (m, 1H), 4.07–4.12 (m, 1H), 4.18–4.23 (m, 1H), 4.28 (t, *J* = 6.4 Hz, 1H), 4.40–4.62 (m, 5H), 4.71–4.77 (m, 3H), 4.85 (dd, *J* = 40.0, 16.0 Hz, 2H), 5.12 (s, 2H), 5.19 (s, 1H), 6.40 (br s, 2H), 7.17 (t, *J* = 9.2 Hz, 1H), 7.24–7.36 (m, 4H), 7.55–7.63 (m, 4H), 7.69–7.80 (m, 6H), 7.90 (t, *J* = 6.0 Hz, 1H,), 8.00 (d, *J* = 8.0 Hz, 2H), 8.09 (d, *J* = 7.2 Hz, 1H), 8.19 (s, 1H), 8.31 (t, *J* = 7.6 Hz, 2H), 8.44 (d, *J* = 8.8 Hz, 1H), 8.55 (d, *J* = 7.6 Hz, 1H). ^13^C-NMR (100 MHz, *d*_6_-DMSO): 195.4, 172.0, 171.9, 171.7, 170.7, 168.4, 166.2, 165.7, 162.7, 158.1, 150.6, 145.5, 142.7, 141.8, 139.2, 137.4, 136.7, 136.2, 132.9, 132.0, 130.8, 129.7, 129.3, 129.1, 128.9, 128.7, 128.6, 128.1, 128.0, 127.7, 127.3, 125.1, 123.7, 119.6, 115.4, 107.4, 101.2, 66.9, 65.3, 63.6, 61.0, 59.2, 57.6, 55.4, 53.3, 51.9, 48.5, 47.1, 45.1, 42.4, 38.1, 35.2, 34.2, 31.0, 30.3, 29.1, 28.7, 28.2, 28.0, 25.3, 22.8, 22.7, 21.3, 20.6, 14.7. ESI (MS) *m*/*z*: 1454.5 (*M* + H)^+^. HRMS (ESI-TOF) calculated for C_73_H_84_ClN_11_NaO_15_S_2_ (*M* + Na)^+^: 1476.5176; found: 1476.5171.

*Isopropyl 1-[2-(2-{2-[4-({[(2-[(S)-6-dansylamide-2-(4-benzoylbenzamido)hexanamido] -6-(5-{(3aS,4S,6aR)-2-oxohexahydro-1H-thieno[3,4-d]imidazol-4-yl}pentanamido) hexanoyl]oxy}methyl)-1H-1,2,3-triazol-1-yl]ethoxy}ethoxy)-2-oxoethyl]-4-(2-chlorophenyl)-2-methyl-5-oxo-1,4,5,7-tetrahydrofuro[3,4-b]pyridine-3-carboxylate* (**4b**). HPLC analysis: 98.2%. M.p. 133–135 °C. ^1^H-NMR (400 MHz, *d*_6_-DMSO): 0.76 (d, *J* = 6.0 Hz, 3H), 1.10 (d, *J* = 6.4 Hz, 3H), 1.28–1.61 (m, 18H), 2.03 (t, *J* = 7.6 Hz, 2H), 2.28 (s, 3H), 2.55 (d, *J* = 12.4 Hz, 1H), 2.75–2.80 (m, 3H), 2.82 (s, 6H), 2.95–3.00 (m, 2H), 3.04–3.09(m, 1H), 3.66 (t, *J* = 4.4 Hz, 2H), 3.84 (t, *J* = 5.2 Hz, 2H), 4.08–4.11 (m, 1H), 4.18–4.23 (m, 1H), 4.26–4.30 (m, 3H), 4.38–4.60 (m, 5H), 4.71–4.77 (m, 1H), 4.89 (dd, *J* = 34.4, 16.0 Hz, 2H), 5.14 (d, *J* = 3.2 Hz, 2H), 5.21 (s, 1H), 6.40 (br s,2H), 7.17 (td, *J* = 8.0, 0.8 Hz, 1H), 7.24–7.28 (m, 2H), 7.32 (d, *J* = 8.0 Hz, 1H), 7.40 (d, *J* = 7.6 Hz, 1H), 7.55–7.63 (m, 4H), 7.69–7.80 (m, 6H), 7.90 (t, *J* = 5.6 Hz, 1H), 8.01 (d, *J* = 8.0 Hz, 2H), 8.09 (d, *J* = 6.0 Hz, 2H), 8.31 (t, *J* = 6.4 Hz, 2H), 8.44 (d, *J* = 8.4 Hz, 1H), 8.55 (d, *J* = 7.6 Hz, 1H). ^13^C-NMR (100 MHz, *d*_6_-DMSO): 195.4, 172.0, 171.9, 171.8, 170.8, 168.7, 166.2, 165.7, 162.7, 158.2, 150.6, 145.6, 142.8, 141.5, 139.2, 137.4, 136.7, 136.2, 133.0, 132.0, 130.9, 129.7, 129.3, 129.1, 128.8, 128.7, 128.6, 128.1, 128.0, 127.7, 127.6, 127.3, 124.9, 123.7, 119.6, 115.3, 107.5, 101.2, 68.5, 67.9, 66.9, 65.4, 64.4, 61.0, 59.2, 57.6, 55.4, 53.2, 51.9, 49.3, 47.3, 45.1, 42.4, 38.1, 35.2, 34.2, 31.0, 30.3, 29.1, 28.7, 28.2, 27.9, 25.3, 22.8, 22.7, 21.3, 20.6, 14.7. ESI (MS) *m*/*z*: 1499.0 (*M* + H)^+^. HRMS (ESI-TOF) calculated for C_75_H_88_ClN_11_NaO_16_S_2_ (*M* + Na)^+^: 1520.5438; found: 1520.5433.

*Isopropyl 1-{2-[2-(2-{2-[4-({[(2-[(S)-6-dansylamide-2-(4-benzoylbenzamido)hexanamido] -6-(5-{(3aS,4S,6aR)-2-oxohexahydro-1H-thieno[3,4-d]imidazol-4-yl}pentanamido) hexanoyl]oxy}methyl)-1H-1,2,3-triazol-1-yl]ethoxy}ethoxy)ethoxy]-2-oxoethyl}-4-(2-chlorophenyl)-2-methyl-5-oxo-1,4,5,7-tetrahydrofuro[3,4-b]pyridine-3-carboxylate* (**4c**). HPLC analysis: 98.9%. M.p. 119–121 °C. ^1^H-NMR (400 MHz, *d*_6_-DMSO): 0.76 (d, *J* = 6.0 Hz, 3H), 1.10 (d, *J* = 6.0 Hz, 3H), 1.28–1.61 (m, 18H), 2.03 (t, *J* = 7.6 Hz, 2H), 2.30 (s, 3H), 2.55 (d, *J* = 12.4 Hz, 1H), 2.77–2.80 (m, 3H), 2.82 (s, 6H), 2.95–3.00 (m, 2H), 3.04–3.09 (m, 1H), 3.49 (s, 4H), 3.61 (t, *J* = 4.4 Hz, 2H), 3.78 (t, *J* = 4.8 Hz, 2H), 4.08–4.11 (m, 1H), 4.18–4.24 (m, 1H), 4.26–4.31 (m, 3H), 4.38–4.61 (m, 5H), 4.71–4.77 (m, 1H), 4.89 (dd, *J* = 32.4, 16.4 Hz, 2H), 5.14 (d, *J* = 3.6 Hz, 2H), 5.21 (s, 1H), 6.40 (br s,2H), 7.17 (t, *J* = 7.6 Hz, 1H), 7.24–7.28 (m, 2H), 7.32 (d, *J* = 8.0 Hz, 1H), 7.40 (d, *J* = 7.2 Hz, 1H), 7.55–7.63 (m, 4H), 7.69–7.80 (m, 6H), 7.89 (t, *J* = 6.0 Hz, 1H), 8.01 (d, *J* = 8.0 Hz, 2H), 8.09 (d, *J* = 4.8 Hz, 2H), 8.31 (t, *J* = 7.6 Hz, 2H), 8.44 (d, *J* = 8.8 Hz, 1H), 8.55 (d, *J* = 8.0 Hz, 1H). ^13^C-NMR (100 MHz, *d*_6_-DMSO): 195.4, 172.0, 171.9, 171.8, 170.8, 168.7, 166.2, 165.7, 162.7, 158.2, 150.5, 145.6, 142.8, 141.5, 139.2, 137.4, 136.7, 136.2, 133.0, 132.0, 130.9, 129.7, 129.3, 129.1, 128.8, 128.7, 128.6, 128.1, 128.0, 127.7, 127.6, 127.3, 124.9, 123.7, 119.6, 115.4, 107.5, 101.1, 69.5, 68.6, 68.1, 66.9, 65.4, 64.5, 61.0, 59.2, 57.7, 55.4, 53.2, 51.9, 49.4, 47.3, 45.1, 42.4, 38.1, 35.2, 34.2, 31.0, 30.3, 29.1, 28.7, 28.2, 28.0, 25.3, 22.8, 22.7, 21.3, 20.7, 14.7. ESI (MS) *m*/*z*: 1544.0 (*M* + H)^+^. HRMS (ESI-TOF) calculated for C_77_H_92_ClN_11_NaO_1__7_S_2_ (*M* + Na)^+^: 1564.5700; found: 1564.5695.

*Isopropyl 1-{14-[4-({[(2-[(S)-6-dansylamide-2-(4-benzoylbenzamido)hexanamido] -6-(5-{(3aS,4S,6aR)-2-oxohexahydro-1H-thieno[3,4-d]imidazol-4-yl}pentanamido) hexanoyl]oxy}methyl)-1H-1,2,3-triazol-1-yl]-2-oxo-3,6,9,12-tetraoxatetradecyl}-4-(2-chlorophenyl)-2-methyl-5-oxo-1,4,5,7-tetrahydrofuro[3,4-b]pyridine-3-carboxylate* (**4d**). HPLC analysis: 99.0%. M.p. 158–160 °C. ^1^H-NMR (400 MHz, *d*_6_-DMSO): 0.77 (d, *J* = 6.4 Hz, 3H), 1.11 (d, *J* = 6.4 Hz, 3H), 1.23–1.61 (m, 18H), 2.03 (t, *J* = 6.8 Hz, 2H), 2.30 (s, 3H), 2.55 (d, *J* = 12.8 Hz, 1H), 2.77–2.80 (m, 3H), 2.82 (s, 6H), 2.95–3.00 (m, 2H), 3.04–3.09 (m, 1H), 3.44–3.50 (m, 8H), 3.64 (t, *J* = 4.4 Hz, 2H), 3.80 (t, *J* = 4.8 Hz, 2H), 4.08–4.11 (m, 1H), 4.19–4.24 (m, 1H), 4.26–4.32 (m, 3H), 4.40–4.61 (m, 5H), 4.71–4.77 (m, 1H), 4.89 (dd, *J* = 32.4,16.4 Hz, 2H), 5.14 (d, *J* = 4.8 Hz, 2H), 5.21 (s, 1H), 6.39 (br s,2H), 7.16 (t, *J* = 7.2 Hz, 1H), 7.25 (t, *J* = 7.6 Hz, 2H), 7.31 (t, *J* = 8.0 Hz, 1H), 7.40 (d, *J* = 7.6 Hz, 1H), 7.54–7.62 (m, 4H), 7.71 (t, *J* = 6.0 Hz, 2H), 7.77 (dd, *J* = 15.2, 8.0 Hz, 4H), 7.88 (t, *J* = 6.0 Hz, 1H), 8.01 (d, *J* = 8.4 Hz, 2H), 8.09 (d, *J* = 5.2 Hz, 2H), 8.30 (d, *J* = 8.8 Hz, 2H), 8.44 (d, *J* = 8.4 Hz, 1H), 8.54 (d, *J* = 7.6 Hz, 1H).^13^C-NMR (100 MHz, *d*_6_-DMSO): 195.4, 172.0, 171.9, 171.8, 170.8, 168.7, 166.2, 165.7, 162.7, 158.2, 150.8, 145.6, 142.8, 141.5, 139.2, 137.4, 136.7, 136.2, 133.0, 132.0, 130.9, 129.7, 129.3, 129.1, 129.0,128.9, 128.7, 128.6, 128.1, 128.0, 127.7, 127.6, 127.3, 125.0,123.6, 119.4, 115.2, 107.5, 101.1, 69.7, 69.6, 69.59, 69.50, 68.6, 68.1, 66.9, 65.4, 64.5, 61.0, 59.2, 57.6, 55.4, 53.2, 51.9, 49.4, 47.3, 45.1, 42.4, 38.1, 35.2, 34.2, 31.0, 30.3, 29.1, 28.7, 28.2, 28.0, 25.3, 22.8, 22.7, 21.3, 20.7, 14.7. ESI (MS) *m*/*z*: 1586.0 (*M* + H)^+^. HRMS (ESI-TOF) calculated for C_79_H_96_ClN_11_NaO_1__8_S_2_ (*M* + Na)^+^: 1608.5962; found: 1608.5957.

#### 3.1.17. General Procedure for Synthesis of compounds **5a**-**5d**

A mixture of **31a**–**31d** (0.37 mmol) and NaN_3_ (0.56 mmol) in DMF (8.0 mL) was stirred at r.t. overnight. The mixture was diluted with ice water (100 mL) and extracted by EtOAc (100 mL × 2). The combined organic phase was washed with brine (100 mL × 2), dried over anhydrous Na_2_SO_4_, and concentrated. The residue was purified by preparative-reverse phase HPLC to give **5a**–**5d** as pale yellow solids.

*Isopropyl 1-(2-{2-[4-({[(S)-6-(2-azidoacetamido)-2-(4-benzoylbenzamido)hexanoyl]oxy}methyl)-1H-1,2,3-triazol-1-yl]ethoxy}-2-oxoethyl)-4-(2-chlorophenyl)-2-methyl-5-oxo-1,4,5,7-tetrahydrofuro[3,4-b]pyridine-3-carboxylate* (**5a**). HPLC analysis: 91.4%. M.p. 81–83 °C. ^1^H-NMR (400 MHz, CDCl_3_): 0.81 (d, *J* = 6.4 Hz, 3H), 1.17 (dd, *J* = 6.4, 2.4 Hz, 3H), 1.36–1.59 (m, 4H), 1.83–1.98 (m, 2H), 2.33 (s, 3H), 3.23–3.32 (m, 2H), 3.85–3.95 (m, 2H), 4.12–4.31 (m, 2H), 4.61–4.69 (m, 7H), 4.81–4.87 (m, 1H), 5.30–5.35 (m, 2H), 5.39 (s, 1H), 6.56–6.60 (m, 1H), 7.07 (tt, *J* = 8.0, 1.2 Hz, 1H), 7.14–7.22 (m, 2H), 7.25–7.27 (m, 1H), 7.32 (d, *J* = 8.0 Hz, 1H), 7.50 (t, *J* = 8.0 Hz, 2H), 7.62 (tt, *J* = 7.2, 1.2 Hz, 1H), 7.74 (d, *J* = 2.8 Hz, 1H), 7.78–7.85 (m, 4H), 7.96 (dd, *J* = 8.4, 3.2 Hz, 2H). ^13^C-NMR (100 MHz, CDCl_3_): 195.1,171.2, 170.7, 166.9, 166.5, 166.0, 165.8, 156.3, 143.6, 141.9, 141.2, 139.3, 135.9, 132.1, 132.0, 130.1, 129.1, 128.9, 128.2, 127.5, 127.0, 126.4, 126.1, 123.6, 108.3, 102.0, 67.0, 64.4, 62.7, 57.3, 52.1, 51.4, 47.9, 46.2, 37.7, 33.9, 29.8, 27.7, 21.7, 20.6, 19.9, 14.2. ESI (MS) *m*/*z*: 950.1 (*M* + H)^+^. HRMS (ESI-TOF) calculated for C_47_H_48_ClN_9_NaO_11_ (*M* + Na)^+^: 972.3060; found: 972.3054.

*Isopropyl 1-[2-(2-{2-[4-({[(S)-6-(2- azidoacetamido)-2-(4-benzoylbenzamido)hexanoyl]oxy}methyl)-1H-1,2,3-triazol-1-yl]ethoxy}ethoxy)-2-oxoethyl]-4-(2-chlorophenyl)-2-methyl-5-oxo-1,4,5,7-tetrahydrofuro[3,4-b]pyridine-3-carboxylate* (**5b**). HPLC analysis: 94.1%. M.p. 67–68 °C. ^1^H-NMR (400 MHz, CDCl_3_): 0.81 (d, *J* = 6.4 Hz, 3H), 1.16 (d, *J* = 6.4 Hz, 3H), 1.34–1.56 (m, 4H), 1.90–1.99 (m, 2H), 2.37 (s, 3H), 3.22–3.29 (m, 2H), 3.68 (t, *J* = 2.8 Hz, 2H), 3.85–3.91 (m, 4H), 4.20–4.38 (m, 4H), 4.50 (t, *J* = 4.8 Hz, 2H), 4.68–4.76 (m, 3H), 4.81–4.88 (m, 1H), 5.31 (s, 2H), 5.41 (s, 1H), 6.62–6.66 (m, 1H), 7.04–7.10 (m, 1H), 7.13–7.22 (m, 2H), 7.25–7.28 (m, 1H), 7.38 (d, *J* = 7.6 Hz, 1H), 7.50 (t, *J* = 7.6 Hz, 2H), 7.62 (t, *J* = 7.6 Hz, 1H), 7.74 (s, 1H), 7.81 (dd, *J* = 15.6, 7.6 Hz, 4H), 7.95 (t, *J* = 8.4 Hz, 2H). ^13^C-NMR (100 MHz, CDCl_3_): 195.0,171.2, 170.6, 167.2, 166.4, 165.8, 165.7, 156.3, 143.7,141.3, 141.2, 139.3, 135.9, 135.8,132.1, 132.0, 130.1, 129.1, 128.9, 128.2, 127.5, 127.0, 126.3, 126.1, 123.9, 108.3, 102.0, 68.1, 67.5, 66.9, 64.5, 63.7, 57.4, 52.0, 51.4, 49.1, 46.4, 37.6, 33.9, 30.0, 27.7, 21.6, 20.6, 19.9, 14.2. ESI (MS) *m*/*z*: 994.1 (*M* + H)^+^. HRMS (ESI-TOF) calculated for C_49_H_52_ClN_9_NaO_12_ (*M* + Na)^+^: 1016.3322; found: 1016.3316.

*Isopropyl 1-{2-[2-(2-{2-[4-({[(S)-6-(2- azidoacetamido)-2-(4-benzoylbenzamido)hexanoyl]oxy}methyl)-1H-1,2,3-triazol-1-yl]ethoxy}ethoxy)ethoxy]-2-oxoethyl}-4-(2-chlorophenyl)-2-methyl-5-oxo-1,4,5,7-tetrahydrofuro[3,4-b]pyridine-3-carboxylate* (**5c**). HPLC analysis: 93.2%. M.p. 73–74 °C. ^1^H-NMR (400 MHz, CDCl_3_): 0.82 (d, *J* = 6.0 Hz, 3H), 1.18 (d, *J* = 6.0 Hz, 3H), 1.38–1.57 (m, 4H), 1.89–2.03 (m, 2H), 2.39(s, 3H), 3.20–3.29(m, 2H), 3.55 (s, 4H), 3.66 (t, *J* = 4.4 Hz, 2H), 3.84 (t, *J* = 5.2 Hz, 2H), 3.90 (d, *J* = 4.0 Hz, 2H), 4.20–4.39 (m,4H), 4.51 (t, *J* = 4.4 Hz, 2H), 4.69–4.77(m, 3H), 4.82–4.88(m, 1H), 5.31(s, 2H), 5.41(s, 1H), 6.65–6.71 (m, 1H), 7.04–7.10 (m, 1H), 7.13–7.18 (m, 1H), 7.22–7.27 (m, 2H), 7.38 (d, *J* = 7.6 Hz, 1H), 7.50 (t, *J* = 7.6 Hz, 2H), 7.62 (t, *J* = 7.6 Hz, 1H), 7.79 (d, *J* = 8.4 Hz, 2H), 7.83 (t, *J* = 3.6 Hz, 3H), 7.95 (t, *J* = 8.4 Hz, 2H). ^13^C-NMR (100 MHz, CDCl_3_): 195.0, 171.1, 170.6, 167.1, 166.3, 165.8, 165.7, 156.2, 143.8, 141.2, 141.1, 139.3, 135.9, 132.1, 132.0, 130.1, 129.1, 129.0, 128.3, 127.5, 126.9, 126.3, 126.1, 124.0, 108.2, 102.1, 69.4, 69.3, 68.2, 67.7, 66.9, 64.4, 63.9, 57.5, 51.9, 51.4, 49.2, 46.4, 37.7, 33.9, 30.2, 27.7, 21.5, 20.6, 19.9, 14.2. ESI (MS) *m*/*z*: 1038.1 (*M* + H)^+^. HRMS (ESI-TOF) calculated for C_51_H_56_ClN_9_NaO_13_ (*M* + Na)^+^: 1060.3584; found: 1060.3578.

*Isopropyl 1-{14-[4-({[(S)-6-(2- azidoacetamido)-2-(4-benzoylbenzamido)hexanoyl]oxy}methyl)-1H-1,2,3-triazol-1-yl]-2-oxo-3,6,9,12-tetraoxatetradecyl}-4-(2-chlorophenyl)-2-methyl-5-oxo-1,4,5,7-tetrahydrofuro[3,4-b]pyridine-3-carboxylate*(**5d**). HPLC analysis: 90.7%. M.p. 66–68 °C. ^1^H-NMR (400 MHz, CDCl_3_): 0.82 (d, *J* = 6.4 Hz, 3H), 1.18 (d, *J* = 6.0 Hz, 3H), 1.38–1.56 (m, 4H), 1.83–1.93 (m, 2H), 2.39 (s, 3H), 3.23–3.29 (m, 2H), 3.54–3.61 (m, 8H), 3.71 (t, *J* = 4.4 Hz, 2H), 3.85(t, *J* = 4.8 Hz, 2H), 3.90 (d, *J* = 4.8 Hz, 2H), 4.27 (dd, *J* = 51.6, 18.8 Hz, 2H), 4.38–4.41 (m, 2H), 4.53 (t, *J* = 5.2 Hz, 2H), 4.72 (s, 2H), 4.74–4.77 (m, 1H), 4.82–4.88 (m, 1H), 5.26–5.35(m, 2H), 5.41 (s, 1H), 6.64–6.69 (m, 1H), 7.05–7.10 (m, 1H), 7.15–7.19 (m, 2H), 7.25–7.27 (m, 1H), 7.39 (dd, *J* = 7.6, 1.6 Hz, 1H), 7.50 (t, *J* = 7.6 Hz, 2H), 7.62 (t, *J* = 7.2 Hz, 1H), 7.78–7.85 (m, 5H), 7.94–7.97 (m, 2H). ^13^C-NMR (100 MHz, CDCl_3_): 195.0, 171.1, 170.5, 167.2, 166.3, 165.8, 165.7, 156.1, 143.8, 141.3, 141.1, 139.3, 136.0, 132.1, 132.0, 130.1, 129.1, 129.0, 128.2, 127.5, 126.9, 126.3, 126.1, 124.1, 108.2, 102.1, 69.45, 69.43, 69.41, 68.2, 67.7, 66.8, 64.4, 63.9, 57.4, 51.9, 51.4, 49.3, 46.4, 37.7, 33.9, 30.2, 27.7, 21.5, 20.6, 19.9, 14.2. ESI (MS) *m*/*z*: 1082.1 (M+H)^+^. HRMS (ESI-TOF) calculated for C_53_H_60_ClN_9_NaO_14_ (*M* + Na)^+^: 1104.3846; found: 1104.3840.

#### 3.1.18. (*S*)-Methyl-6-(2-azidoacetamido)-2-(4-benzoylbenzamido)hexanoate (**34**)

A mixture of **33** (0.11 g, 0.25 mmol) and NaN_3_ (0.25 g, 0.38 mmol) in DMF (10.0 mL) was stirred at 75 °C for 2 h. The mixture was diluted with ice water (20 mL) and extracted by EtOAc (20 mL × 2). The combined organic phase was washed with brine (20 mL × 2), dried over anhydrous Na_2_SO_4_, and concentrated. The residue was purified by flash silica gel column chromatography [petroleum ether-EtOAc (4:1)] to obtain **34** (83.0 mg, 61%) as a white solid product. HPLC analysis: 96.0%. M.p. 121–122 °C. ^1^H-NMR (400 MHz, CDCl_3_): 1.36–1.54 (m, 2H), 1.62 (tt, *J* = 14.1, 7.0 Hz, 2H), 1.88 (ddd, *J* = 22.8, 11.4, 7.4 Hz, 1H), 1.96–2.07 (m, 1H), 3.24–3.39 (m, 2H), 3.80 (s, 3H), 3.88–4.00 (m, 2H), 4.81 (dd, *J* = 12.2, 7.2 Hz, 1H), 6.44 (s, 1H), 6.97 (s, 1H), 7.50 (t, *J* = 7.6 Hz, 2H), 7.62 (t, *J* = 7.4 Hz, 1H), 7.80 (d, *J* = 7.6 Hz, 2H), 7.85 (d, *J* = 7.6 Hz, 2H), 7.96 (d, *J* = 8.2 Hz, 2H). ^13^C-NMR (100 MHz, CDCl_3_): 195.9, 172.8, 166.9, 166.4, 140.4, 137.0, 136.9, 132.9, 130.1, 128.5, 127.2, 52.7, 52.5, 38.7, 31.8, 28.9, 22.4. ESI (MS) *m*/*z*: 450.2 (*M* − H)^−^. HRMS (ESI-TOF) calculated for C_23_H_24_N_5_O_5_ (*M*−H)^−^: 450.1777; found: 450.1783.

#### 3.1.19. Isopropyl 1-(14-{4-[({(*S*)-2-(4-benzoylbenzamido)-6-[2-(4-{[(5-{(3a*S*,4*S*,6a*R*)-2-oxohexahydro-1*H*-thieno[3,4-d]imidazol-4-yl}pentanoyl)oxy]methyl}-1*H*-1,2,3-triazol-1-yl)acetamido]hexanoyl}oxy)methyl]-1*H*-1,2,3-triazol-1-yl}-2-oxo-3,6,9,12-tetraoxatetradecyl)-4-(2-chlorophenyl)-2-methyl-5-oxo-1,4,5,7-tetrahydrofuro[3,4-b]pyridine-3-carboxylate (**36**)

To a solution of **5d** (0.39 g, 0.36 mmol) and **35** (0.12 g, 0.43 mmol) in CH_2_Cl_2_ (4 mL) and H_2_O (4 mL), CuSO_4_·5H_2_O (0.10 mg, 0.40 mmol) and sodium ascorbate (0.10 g, 0.51 mmol) were added. The resulting solution was stirred at r.t. overnight. The solvent was evaporated in vacuo, and then the residue was redissolved in EtOAc (10 mL), washed by water (10 mL × 2), and dried over anhydrous Na_2_SO_4_. The organic layer was evaporated, and the residue was purified by preparative-reverse phase HPLC to give **36** (66.0 mg, 13%) as a pale yellow solid. HPLC analysis: 93.6%. M.p. 71–73 °C. ^1^H-NMR (400 MHz, *d*_6_-DMSO): 0.76 (d, *J* = 6.0 Hz, 3H), 1.11 (d, *J* = 6.0 Hz, 3H), 1.29–1.56 (m, 10H), 1.79–1.84 (m, 2H), 2.30–2.33 (m, 5H), 2.57 (d, *J* = 12.4 Hz, 1H), 2.81 (dd, *J* = 12.4, 5.2 Hz, 1H), 3.06–3.10 (m, 3H), 3.44–3.52 (m, 8H), 3.64 (t, *J* = 4.4 Hz, 2H), 3.80 (t, *J* = 5.2 Hz, 2H), 4.10–4.13 (m, 1H), 4.28–4.32 (m, 3H), 4.43–4.61 (m, 5H), 4.71–4.77 (m, 1H), 4.89 (dd, *J* = 32.0, 16.8 Hz, 2H), 5.06 (s, 2H), 5.12 (s, 2H), 5.21 (s, 3H), 6.34 (br s, 1H), 6.40 (br s, 1H), 7.17 (td, *J* = 7.6, 1.6 Hz, 1H), 7.27 (td, *J* = 7.6, 1.2 Hz, 1H), 7.31 (dd, *J* = 8.0, 1.2 Hz, 1H), 7.40 (dd, *J* = 7.6, 1.6 Hz, 1H), 7.58 (t, *J* = 7.6 Hz, 2H), 7.71 (t, *J* = 7.6 Hz, 1H), 7.76 (d, *J* = 7.2 Hz, 2H), 7.82 (d, *J* = 8.0 Hz, 2H), 8.03 (d, *J* = 8.4 Hz, 2H), 8.07 (s, 1H), 8.12 (s, 1H), 8.32 (t, *J* = 5.6 Hz, 1H), 8.95 (d, *J* = 7.2 Hz, 1H). ^13^C-NMR (100 MHz, CDCl_3_): 195.1, 172.4, 171.6, 170.7, 167.3, 165.9, 165.8, 164.6, 156.3, 143.9, 141.9, 141.3, 141.1, 139.2, 136.0, 135.9, 132.1, 131.9, 130.1, 129.1, 129.0, 128.2, 127.5, 126.9, 126.5, 126.1, 124.6, 124.1, 108.1, 102.0, 69.4, 69.3, 68.2, 67.7, 66.8, 64.5, 63.9, 60.7, 59.2, 57.4, 56.4, 54.5, 52.2, 51.6, 49.3, 46.4, 39.5, 38.0, 33.9, 32.7, 29.9, 27.3, 27.1, 27.0, 23.6, 21.7, 20.7, 19.9, 14.2. ESI (MS) *m*/*z*: 1364.1 (*M* + H)^+^. HRMS (ESI-TOF) calculated for C_66_H_78_ClN_11_NaO_17_S (*M* + Na)^+^: 1386.4884; found: 1386.4879.

### 3.2. Glycogenolysis Inhibition in Liver HL-7702 Cells and HepG2 Cells

The inhibition of hepatic glycogenolysis was monitored by the measurement of liver glycogen using a microplate reader (BIO-RAD, Bio-Rad Laboratories, Hercules, CA, USA), which was done quantitatively by the anthrone reagent (Sigma, Saint Louis, MO, USA) colorimetric method based on the published method [11]. Primary human liver HL-7702 cells or HepG2 cells (Sigma) were treated with the test compound or DMSO solvent (final concentration, 0.10%), followed by 60 min incubation with 0.3 nM glucagon (GGN). Assays were terminated by centrifugation, and cells were digested with 30% KOH followed by glycogen determination. The IC_50_ values were estimated by fitting the inhibition data to a dose-dependent curve using a logistic derivative equation.

### 3.3. Photoaffinity Labeling Experiment

(1) According to our previous report [10], a complete Dulbecco’s Modified Eagle’s Medium (DMEM) (100 U/mL penicillin, 100 mg/mL streptomycin, 12% fetal bovine serum, pH 7.4) was used to culture the HepG2 cells in standard incubator conditions (37 °C, 5% CO_2_) until about 80% confluence. HepG2 cells were harvested by trypsinization and washed twice with phosphate buffered saline (PBS). HepG2 cells were lysed in a lysis buffer (7 M urea, 2 M thiourea, 4% (*w*/*v*) CHAPS, 40 mM Tris, 65 mM DTT, 2% (*v*/*v*) IPG Buffer pH 3–10, 1% (*v*/*v*) Protease Inhibitor cocktail), and then centrifuged using a Soniprep instrument with 100 W, 5 × 10 s pulses and short pauses in between; these cells were kept on ice for 1 h. The resulting lysate was centrifuged at 100,000× *g* at 4 °C for 1 h to remove cell debris. Finally, the precipitate was resuspended in 5 volumes of 50 mM Tris·HCl buffer (pH 7.4) and stored at −80 °C. The protein concentration of the suspension was measured according to the Bradfor method. (2) The soluble proteomes prepared from HepG2 cells were diluted to 2.0 mg/mL with 50 mM Tris·HCl buffer (pH 7.4). The labeling reation was initiated by incubating proteomes with the probe (dissolved in DMSO) at 4 °C for 8 h, and then exposing them to UV 365 nm (220 v, 6 W, 365 nm) at a distance of 3 cm. The reaction mixture was centrifuged at 48,000× *g* for 10 min, and then the supernatant was removed, and the precipitate was resuspended in a lysis buffer (urea 480 mg/L, chaps 40 mg/L, Tris-base 4.8 mg/L, DTT 10 mg/L, Ampholate 50 mL/L, and bromophenol blue 0.002%) at 4 °C for 1 h and centrifuged at 18,000× *g* for 2 h. The supernatant was dialyzed against 50 mM Tris·HCl buffer (pH 7.4). To detect the proteins photo-cross-linked by test probe, the homogenate photolabeled with the probe was subjected to SDS-PAGE electrophoresis and then transferred onto a polyvinylidene fluoride membrane. The membrane was blocked with 1% BSA in PBS with 0.05% Tween 20 at room temperature for 1h, washed using PBS with 0.05% Tween 20 for 5 min two times, and then incubated with a streptavidin-HRP polymer conjugate (Sigma, Cat. S 2438) diluted 1:10,000 in PBS with 0.05% Tween 20 at room temperature for 1 h. After six washes for 5 min each using PBS with 0.05% Tween 20, the membrane was treated with Enhanced Chemiluminescence (ECL, Amersham, Boston, MA, USA) detection reagents. Biotinylated proteins were visualized by exposure to X-ray and imaging development.

### 3.4. Protein Digestion and Mass Spectrometry

(1) The 170 kDa band was excised from the blot and processed for internal sequence analysis as described [13]. Briefly, in situ digestion was done using 0.01 μg/μL trypsin (in 100 μL of 25 mM NH_4_HCO_3_) for 30 min at 4 °C, until the trypsin was completely absorbed by the gel particles. Then, the resulting mixture was added to 100 μL of 25 mM NH_4_HCO_3_, the supernatant was collected by centrifugation and transfered in another Eppendorf tube. The extraction buffer (5% TFA, 95% ddH_2_O) was added into the pellets for 1 h, and then treated with an extraction buffer (2.5% TFA, 50% ACN, 47.5% ddH_2_O) for 1 h and stored at −20 °C. (2) Mass Spectrometry analysis was carried out using the Tanon-6100 Chemiluminescent Imaging system (Tanon Science and technology Co., Ltd., Shanghai, China). The band densities were calculated with Quantity One software 4.6.2 (Bio-Rad Laboratories, Inc., Hercules, CA, USA). The excised gel was digested with trypsin and subjected to LC-MS/MS. The data were recorded on an LTQ-Orbitrap Fusion mass spectrometer (ThermoFisher Scientific Inc., Waltham, MA, USA) coupled to an Easy-nLC 1000 LC System (ThermoFisher Scientific Inc., Waltham, MA, USA). Label-free MS analysis was performed by Thermo Q-Exactive mass spectrometry, and the MS raw data were processed using MaxQuant software with the Uniprot-Orytolagus cuniculus database.

## 4. Conclusions

In summary, this work described the design and synthesis of novel photoaffinity probes of dihydropyridine derivatives, BAY R3401, and evaluated their potency in an inhibition assay of glycogenolysis. Probe **2d** exhibited the best inhibitory activity, with an IC_50_ value of 4.45 μM and 28.49 μM in primary human liver HL-7702 cells and HepG2 cells, respectively. Photoaffinity labeling experiments were also performed, and protein bands larger than 170 kDa were tagged by probe **2d** in crude proteomes. These data suggest that the synthesized probe **2d** might be used to label, identify, and purify the potential target enzymes of BAY R3401. Mass spectrometric analysis was used to detect protein bands larger than 170 kDa, and we hypothesise that the PH-interacting proteins, histone H4, hexokinase-2, and solute carrier family 2 might be the target proteins of BAY R3401 based on the results. We are now in the process of verifying these proteins.

## Figures and Tables

**Table 1:** Glycogenolysis inhibition assay for compounds **2(a**–**d)**–**6(a**–**d)** in liver cells.

Compound	IC_50_^a^ (μM, HL-7702 cells)	IC_50_^a^ (μM, HepG2 cells)	Compound	IC_50_^a^ (μM, HL-7702 cells)	IC_50_^a^ (μM, HepG2 cells)
**2a**	6.23 ± 2.86	53.12 ± 10.11	**4d**	17.15 ± 4.97	NI
**2b**	4.55 ± 1.57	37.69 ± 1.76	**5a**	2.96 ± 0.57	45.94 ± 12.44
**2c**	5.22 ± 1.17	38.00 ± 1.06	**5b**	4.83 ± 1.48	59.89 ± 19.75
**2d**	4.45 ± 0.50	28.49 ± 3.38	**5c**	15.22 ± 3.29	18.73 ± 5.31
**3a**	4.86 ± 1.22	106.16 ± 4.17	**5d**	6.46 ± 3.60	15.29 ± 4.31
**3b**	5.62 ± 1.67	44.75 ± 7.20	**6a**	19.36 ± 2.00	40.02 ± 5.55
**3c**	2.56 ± 0.59	42.32 ± 5.78	**6b**	22.36 ± 1.97	54.38 ± 13.73
**3d**	2.71 ± 0.54	33.71 ± 1.23	**6c**	22.35 ± 24.36	NI
**4a**	27.22 ± 4.94	49.09 ± 1.23	**6d**	24.00 ± 12.44	40.05 ± 3.99
**4b**	27.32 ± 9.27	96.71 ± 36.04	BAY R3401	27.06 ± 9.63	52.83 ± 8.93
**4c**	15.22 ± 3.29	NI^b^	CP-91149 ^c^	2.53 ± 0.78	3.08 ± 1.16

^a^ Each value represents the mean ± S.D. of three determinations. ^b^ NI means no inhibition. ^c^ CP-91149 was used as a positive control.

**Table 2:** Comparative analysis of the protein groups identified by LC-MS/MS.

Description	Accession ^a^	Score ^b^	MW ^c^ [kDa]
Carbamoyl-phosphate synthase [ammonia]	P31327	703.64	164.83
Myoferlin	Q9NZM1	491.05	234.56
Coatomer subunit alpha	P53621	338.49	138.26
Keratin, type II cytoskeletal 1	P04264	332.99	66.0
Vigilin	Q00341	325.29	141.37
Sodium/potassium-transporting ATPase subunit alpha-1	P05023	169.27	112.82
Inositol 1,4,5-trisphosphate receptor type 3	Q14573	146.90	303.91
Epidermal growth factor receptor	P00533	110.92	134.19
PH-interacting protein	Q8WWQ0	46.67	206.56
Nuclear pore complex protein Nup153	P49790	29.84	153.84
Histone H4	P62805	27.37	11.36
Hexokinase-2	E9PB90	26.18	98.91
DNA (cytosine-5)-methyltransferase 1	P26358	26.13	183.05
U2 snRNP-associated SURP motif-containing protein	O15042	25.91	118.22
Histone-lysine *N*-methyltransferase EHMT2	A0A0G2JIS2	24.46	128.95
Keratin, type II cytoskeletal 72	Q14CN4	24.37	55.84
1-phosphatidylinositol 4,5-bisphosphate phosphodiesterase beta-3	Q01970	22.73	138.71
Solute carrier family 2, facilitated glucose transporter member 1	P11166	20.46	54.05
Inositol 1,4,5-trisphosphate receptor type 2	Q14571	20.32	307.87
Low-density lipoprotein receptor	P01130	18.50	95.31
Phosphatidylinositol 4-phosphate 3-kinase C2 domain-containing subunit alpha	O00443	16.86	190.56
Inositol 1,4,5-trisphosphate receptor type 1	Q14643	15.87	313.73
Receptor tyrosine-protein kinase erbB-2 (Fragment)	J3KTI5	13.12	27.76
Apolipoprotein B-100	P04114	11.42	515.28
Rho-associated protein kinase 2	O75116	10.68	160.80
Histone-lysine *N*-methyltransferase EHMT1	A0A1B0GV09	10.33	138.17

^a^ Protein accession number received from the Uniprot-Orytolagus cuniculus database. ^b^ Scores are based on the results of MaxQuant software (www.maxquant.org). The higher the score, the more consistent the mass spectrometry data are with data in the database. ^c^ The molecular mass data were recorded according to the LTQ-Orbitrap Fusion mass spectrometer.

## Supplementary Materials

Supplementary materials associated with this article are available online. Contents: Data of Coomassie brilliant blue (CBB) and NMR spectra of the prepared compounds.
