# Supplementary material for: Design, Synthesis, and Biological Application of Novel Photoaffinity Probes of Dihydropyridine Derivatives, BAY R3401
Source: Molecules. 2019 Jun 28;24(13):2394. doi: 10.3390/molecules24132394 (PMC6652023; doi:10.3390/molecules24132394)
Supplement: Supplementary file 1 [file molecules-24-02394-s001.pdf]

Supporting Information

Design, Synthesis, Biological Application of Novel Photoaffinity Probes of Dihydropyridine Derivative, BAY R3401

Liying Zhang [1,*], Zhiwei Yan [1], Youde Wang [1], Chengjun Song [2], Guangxin Miao [1].

Contents

Coomassie brilliant blue (CBB) poststaining and destaining protocol 3
Figure S1: Copy of ^{1}H NMR and ^{13}C NMR of 10 4
Figure S2: Copy of ^{1}H NMR and ^{13}C NMR of 11 5
Figure S3: Copy of ^{1}H NMR and ^{13}C NMR of 12 6
Figure S4: Copy of ^{1}H NMR and ^{13}C NMR of 13a 7
Figure S5: Copy of ^{1}H NMR and ^{13}C NMR of 13b 8
Figure S6: Copy of ^{1}H NMR and ^{13}C NMR of 13c 9
Figure S7: Copy of ^{1}H NMR and ^{13}C NMR of 13d 10
Figure S8: Copy of ^{1}H NMR and ^{13}C NMR of 6a 11
Figure S9: Copy of ^{1}H NMR and ^{13}C NMR of 6b 12
Figure S10: Copy of ^{1}H NMR and ^{13}C NMR of 6c 13
Figure S11: Copy of ^{1}H NMR and ^{13}C NMR of 6d 14
Figure S10: Copy of ^{1}H NMR and ^{13}C NMR of 16 15
Figure S11: Copy of ^{1}H NMR and ^{13}C NMR of 18 16
Figure S11: Copy of ^{1}H NMR and ^{13}C NMR of 20 17
Figure S10: Copy of ^{1}H NMR and ^{13}C NMR of 21 18
Figure S11: Copy of ^{1}H NMR and ^{13}C NMR of 22 19
Figure S10: Copy of ^{1}H NMR and ^{13}C NMR of 24 20
Figure S11: Copy of ^{1}H NMR and ^{13}C NMR of 25 21
Figure S11: Copy of ^{1}H NMR and ^{13}C NMR of 27 22
Figure S10: Copy of ^{1}H NMR and ^{13}C NMR of 28 23
Figure S11: Copy of ^{1}H NMR and ^{13}C NMR of 30 24
Figure S11: Copy of ^{1}H NMR and ^{13}C NMR of 2a 25
Figure S11: Copy of ^{1}H NMR and ^{13}C NMR of 2b 26
Figure S11: Copy of ^{1}H NMR and ^{13}C NMR of 2c 27
Figure S11: Copy of ^{1}H NMR and ^{13}C NMR of 2d 28
Figure S11: Copy of ^{1}H NMR and ^{13}C NMR of 3a 29
Figure S11: Copy of ^{1}H NMR and ^{13}C NMR of 3b 30
Figure S11: Copy of ^{1}H NMR and ^{13}C NMR of 3c 31
Figure S11: Copy of ^{1}H NMR and ^{13}C NMR of 3d 32
Figure S11: Copy of ^{1}H NMR and ^{13}C NMR of 4a 33
Figure S11: Copy of ^{1}H NMR and ^{13}C NMR of 4b 34
Figure S11: Copy of ^{1}H NMR and ^{13}C NMR of 4c 35
Figure S11: Copy of ^{1}H NMR and ^{13}C NMR of 4d 36
Figure S11: Copy of ^{1}H NMR and ^{13}C NMR of 31a 37
Figure S11: Copy of ^{1}H NMR and ^{13}C NMR of 31b 38
Figure S11: Copy of ^{1}H NMR and ^{13}C NMR of 31c 39
Figure S11: Copy of ^{1}H NMR and ^{13}C NMR of 31d 40
Figure S11: Copy of ^{1}H NMR and ^{13}C NMR of 32 41
Figure S11: Copy of ^{1}H NMR and ^{13}C NMR of 5a 42
Figure S11: Copy of ^{1}H NMR and ^{13}C NMR of 5b 43

Figure S11: Copy of ^{1}H NMR and ^{13}C NMR of 5c 44
Figure S11: Copy of ^{1}H NMR and ^{13}C NMR of 5d 45
Figure S11: Copy of ^{1}H NMR and ^{13}C NMR of 33 46
Figure S11: Copy of ^{1}H NMR and ^{13}C NMR of 34 47
Figure S11: Copy of ^{1}H NMR and ^{13}C NMR of 36 48
Image of the SDS-PAGE gel stained with Coomassie R-250 49
References 50

Coomassie brilliant blue (CBB) poststaining and destaining protocol.

The soluble proteomes prepared from HepG2 cells was diluted to 2.0 mg/mL with 50 mM Tris HCl buffer (pH 7.4). The labeling reation was initiated by incubating proteomes with the probe (dissolved in DMSO) at 4 °C for 8 h, and then exposed to UV 365 nm (220v, 6W, 365 nm) at a distance of 3 cm. The reaction mixture was centrifuged at 48000 g for 10 min, then the supernatant was removed and the precipitate was resuspended in lysis buffer (urea 480 mg/L, chaps 40 mg/L, Tris-base 4.8 mg/L, DTT 10 mg/L, Ampholate 50 ml/L, and bromophenol blue 0.002%) at 4 °C for 1 h and centrifuged at 18000 g for 2 h. The supernatant was dialyzed against 50 mM Tris HCl buffer (pH 7.4) and then subjected to SDS-PAGE electrophoresis. The samples were analyzed on Coomassie blue staining, based on the published method.[1] 2.5 g of Coomassie blue R250 was dissolved in 1000 mL of 50% (v/v) methanol, 10% (v/v) acetic acid and 40% (v/v) water with stirring as needed. The solution was filtered to remove any insoluble material. The final concentration of Coomassie blue R250 was 0.25% (w/v). After electrophoresis, the apparatus was disassembled and the gel was immersed into CBB solution. The gel was stained at room temperature overnight with gentle agitation. The Coomassie stain was removed by aspiration after staining. The gel was then immersed into the destaining solution composed of 50% (v/v) methanol, 10% (v/v) acetic acid and 40% (v/v) water which allowed the gel to destain with gentle agitation. The destaining step was repeated several times with removal of destaining solution at each change by aspiration. Destaining was continued until the protein bands were seen clearly without any background staining of the gel.

^{1}H NMR spectra of compound **10**

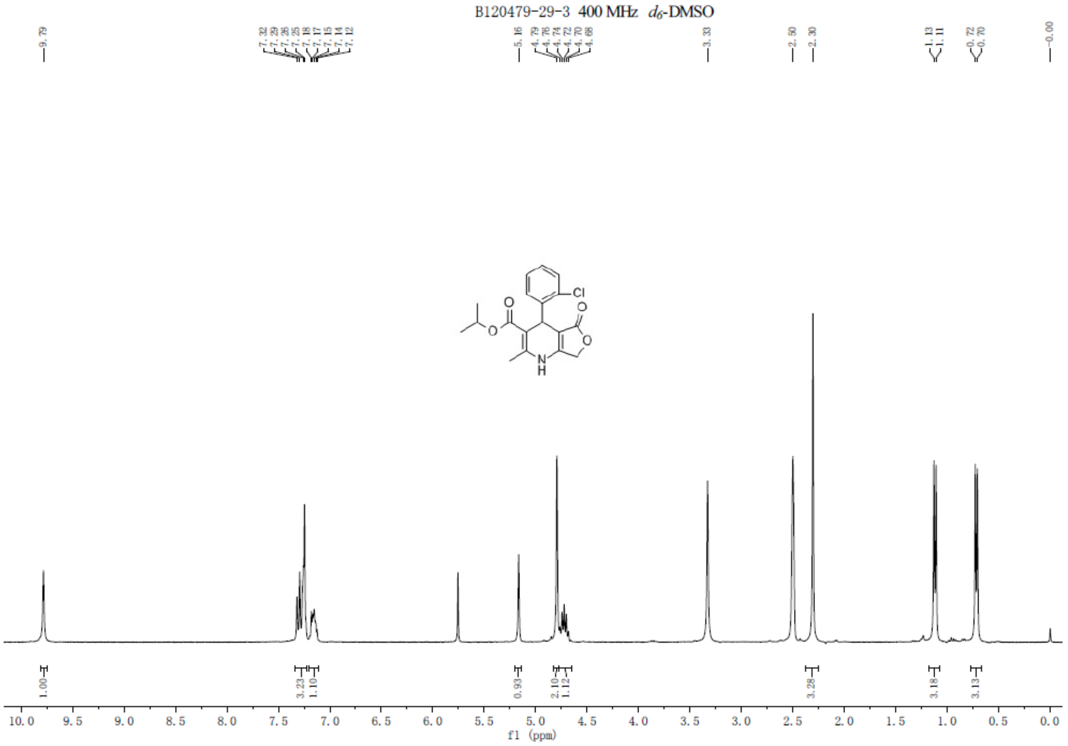

^{13}C NMR spectra of compound **10**

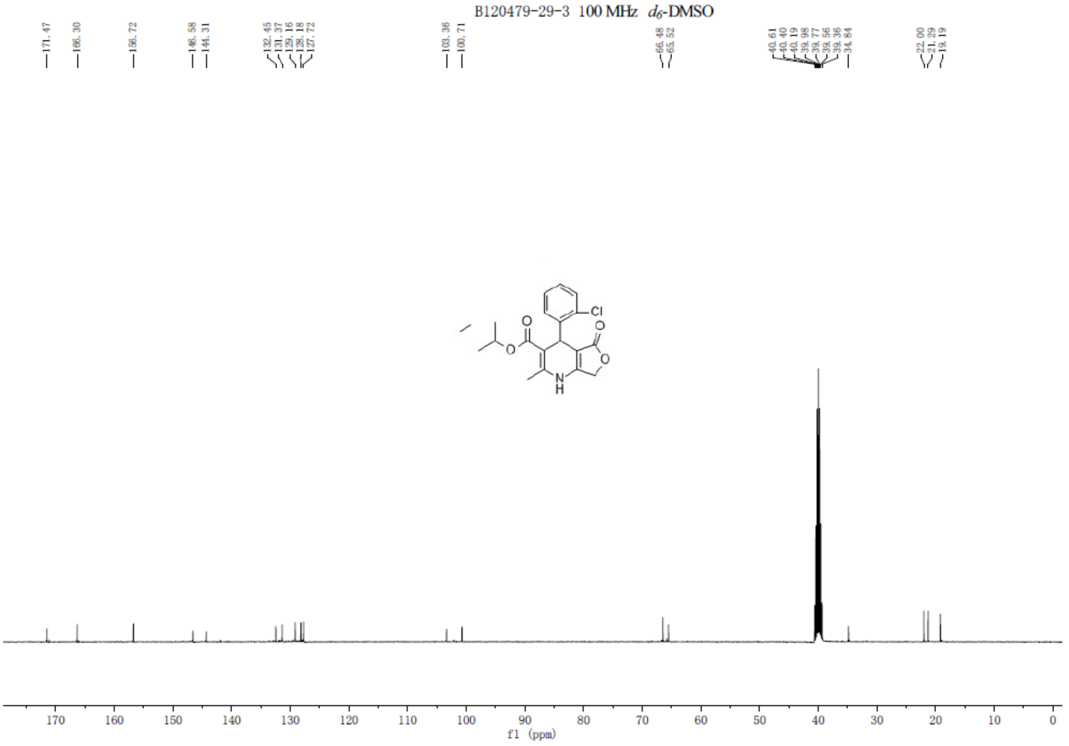

^{1}H NMR spectra of compound **11**

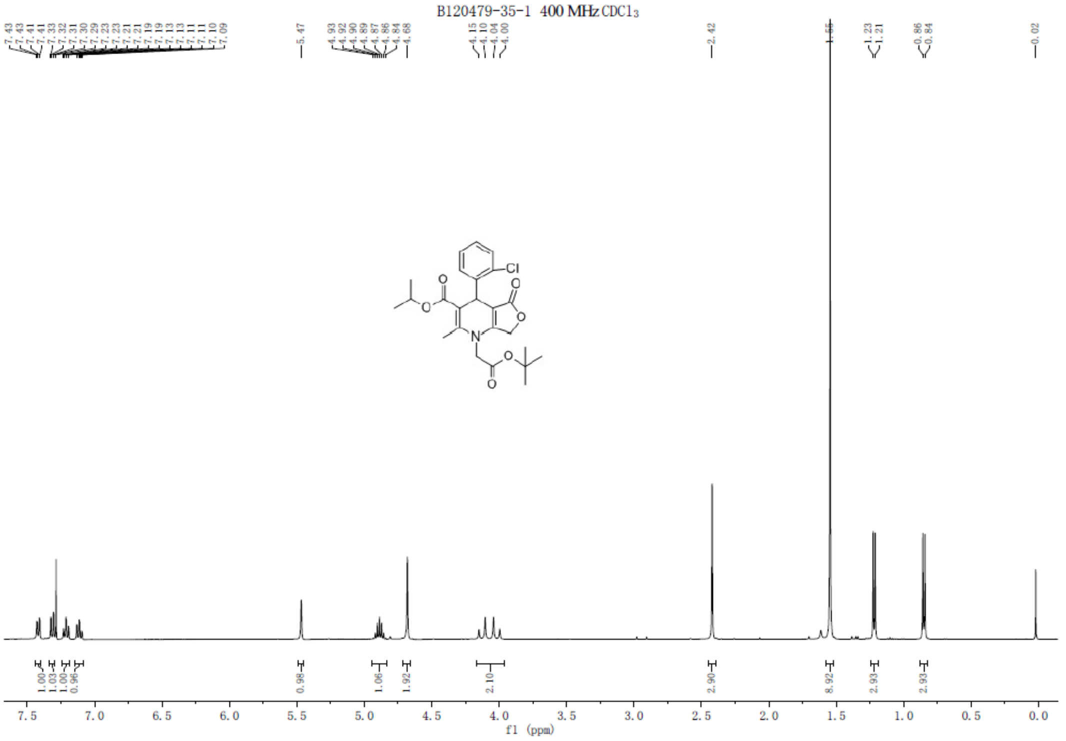

^{13}C NMR spectra of compound **11**

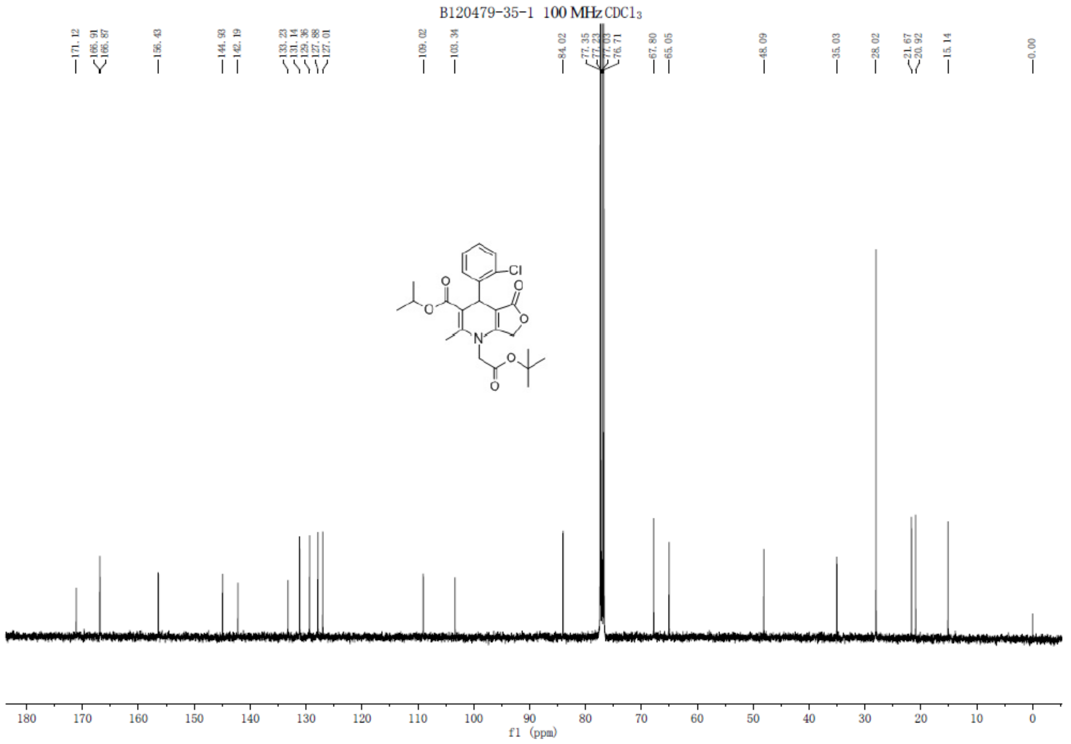

^{1}H NMR spectra of compound **12**

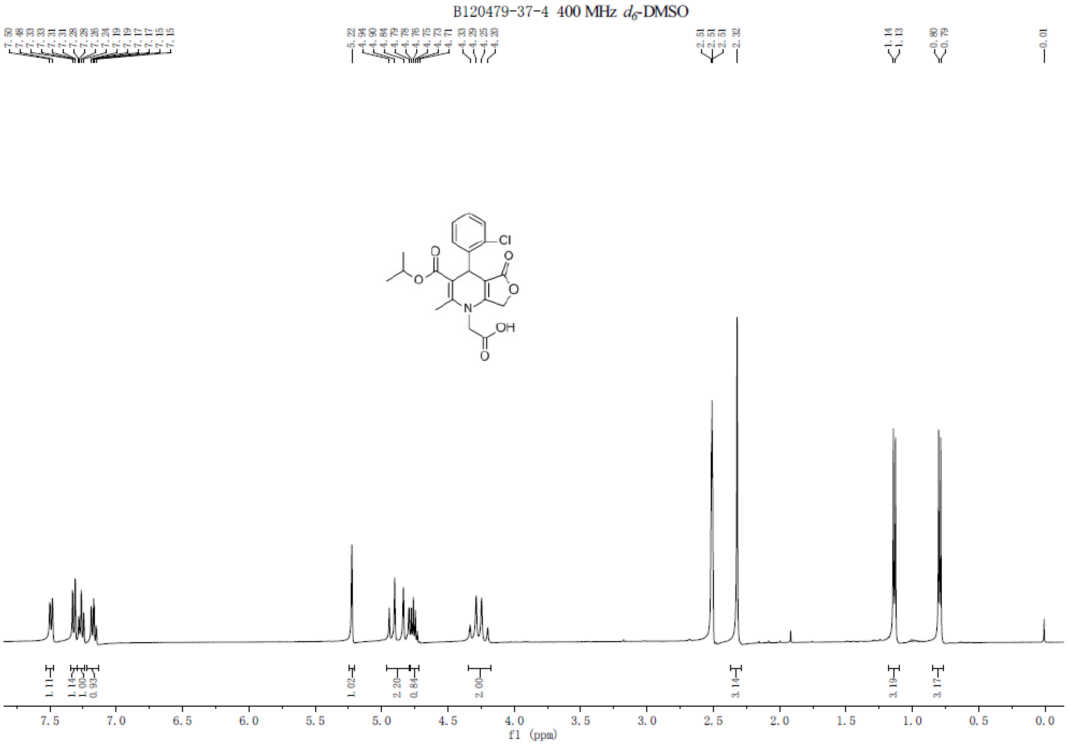

^{13}C NMR spectra of compound **12**

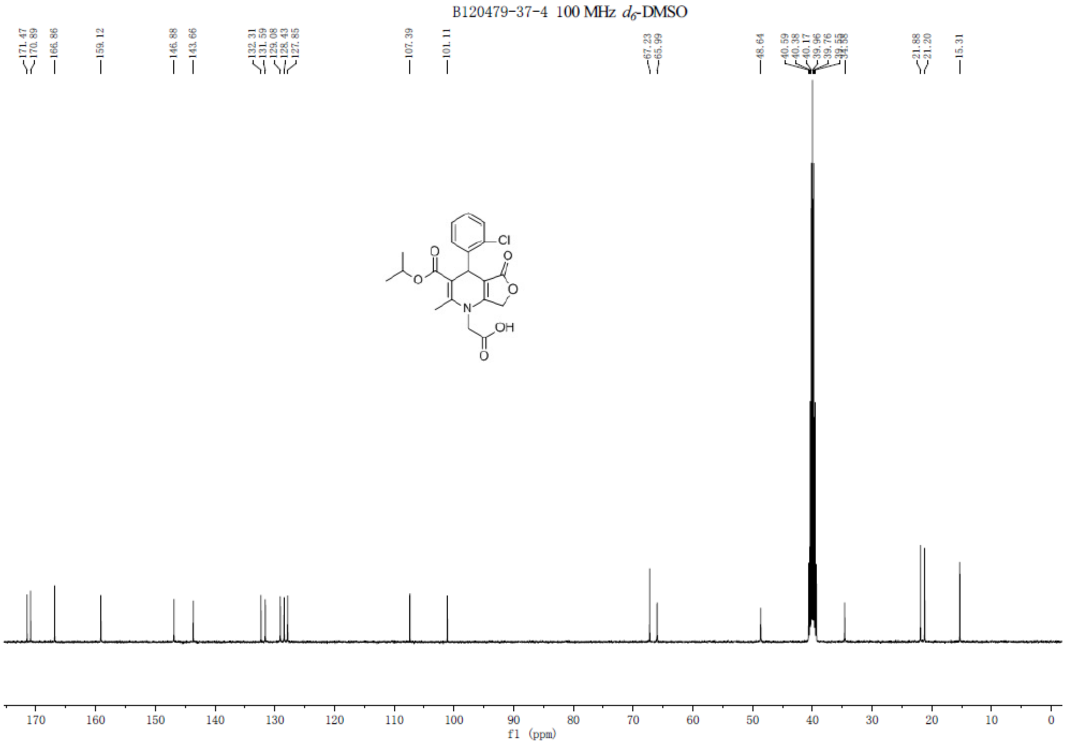

^{1}H NMR spectra of compound **13a**

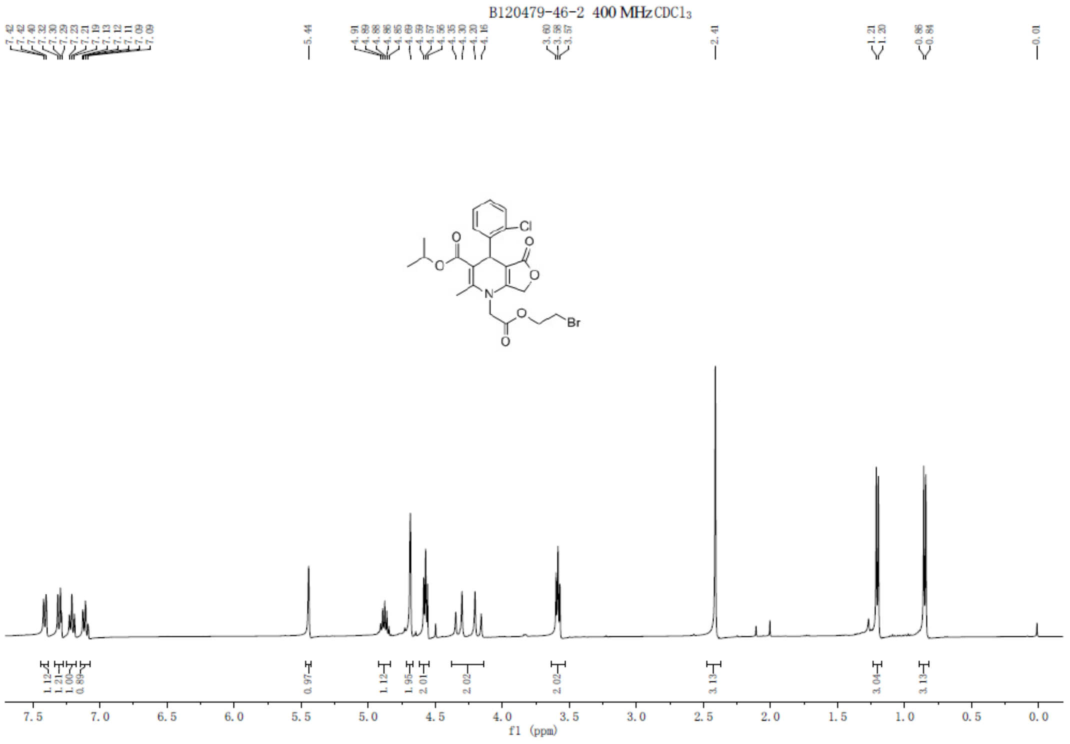

^{13}C NMR spectra of compound **13a**

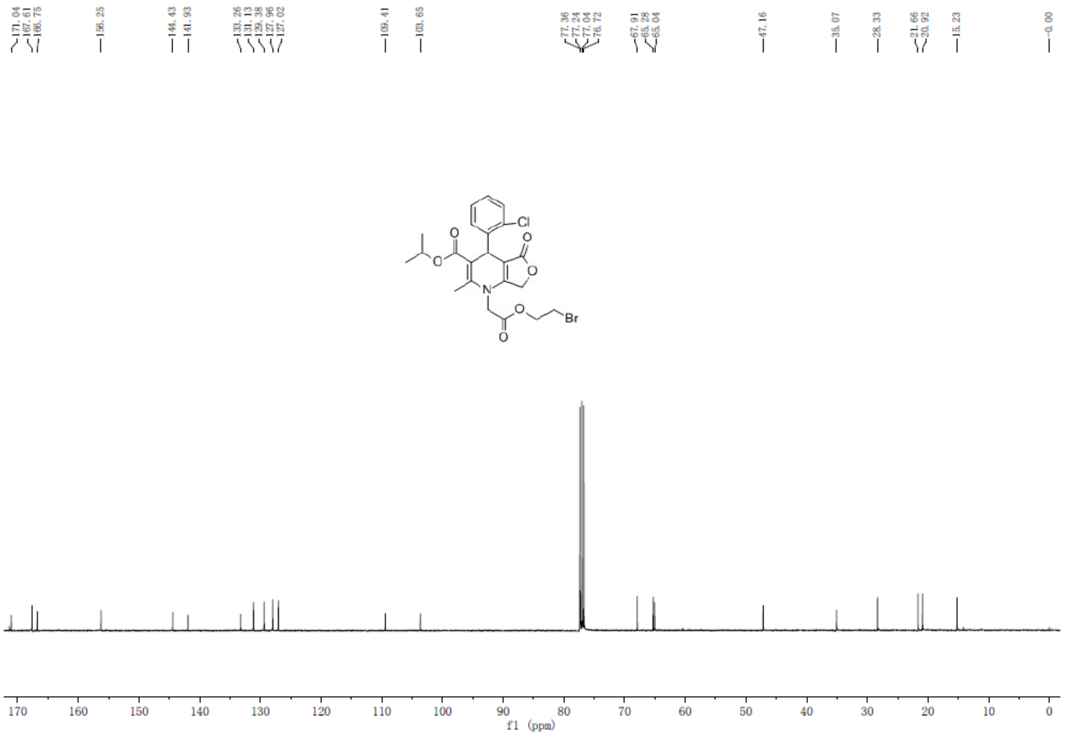

^{1}H NMR spectra of compound **13b**

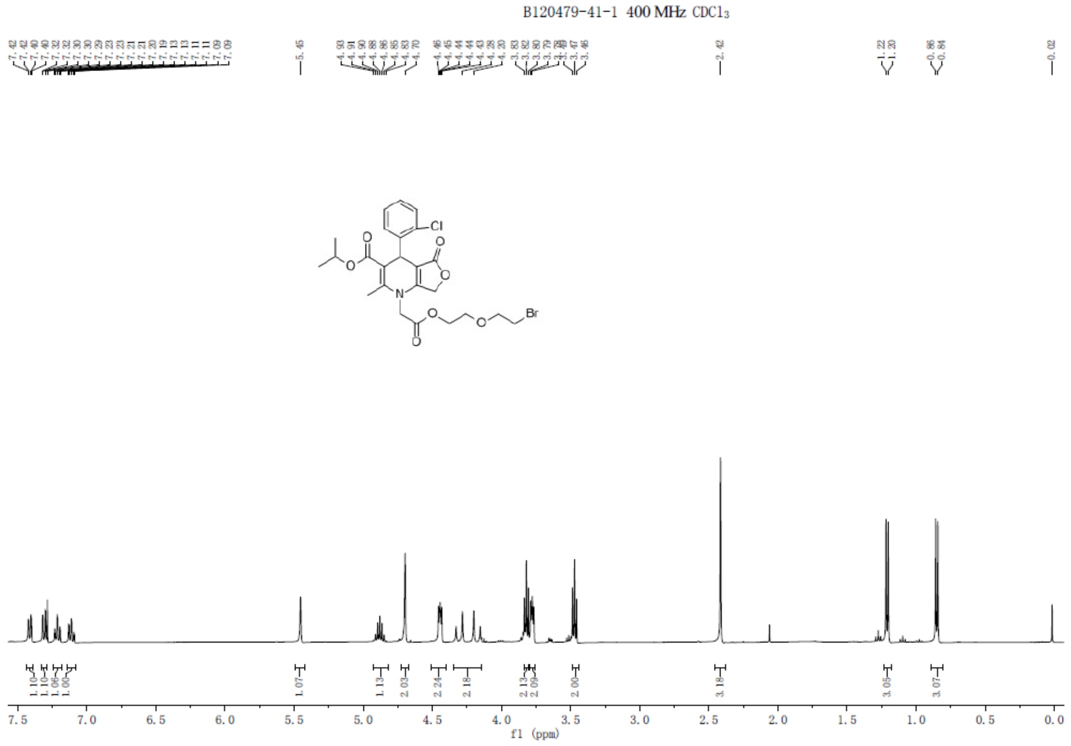

^{13}C NMR spectra of compound **13b**

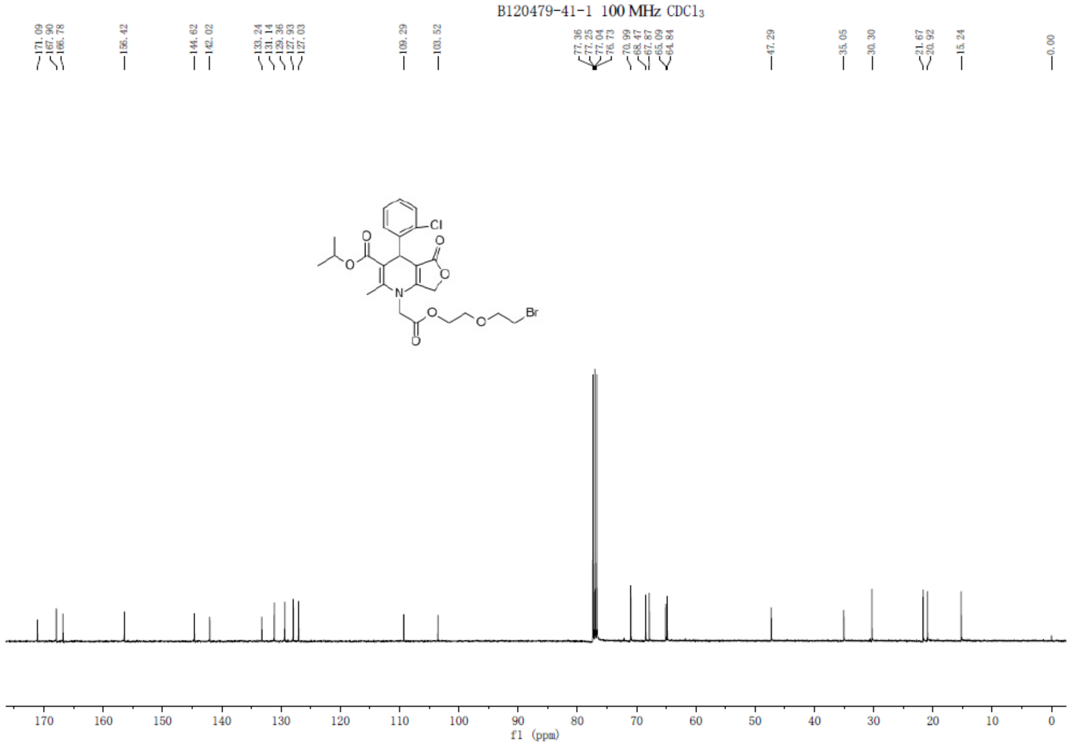

^{1}H NMR spectra of compound **13c**

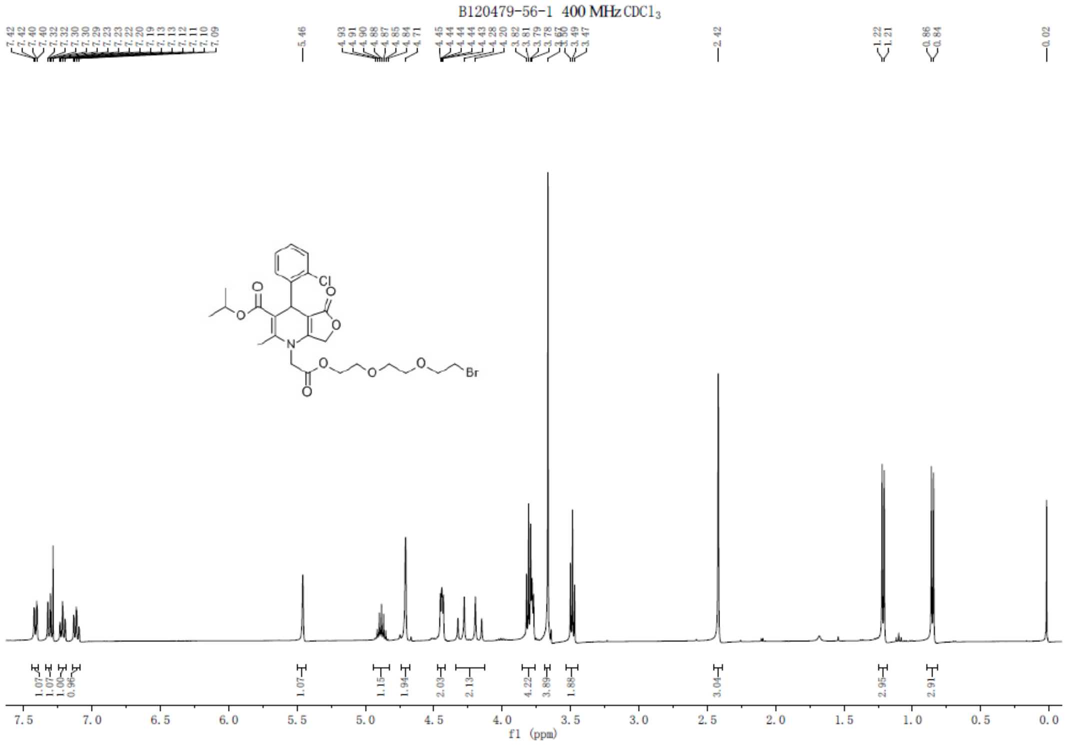

^{13}C NMR spectra of compound **13c**

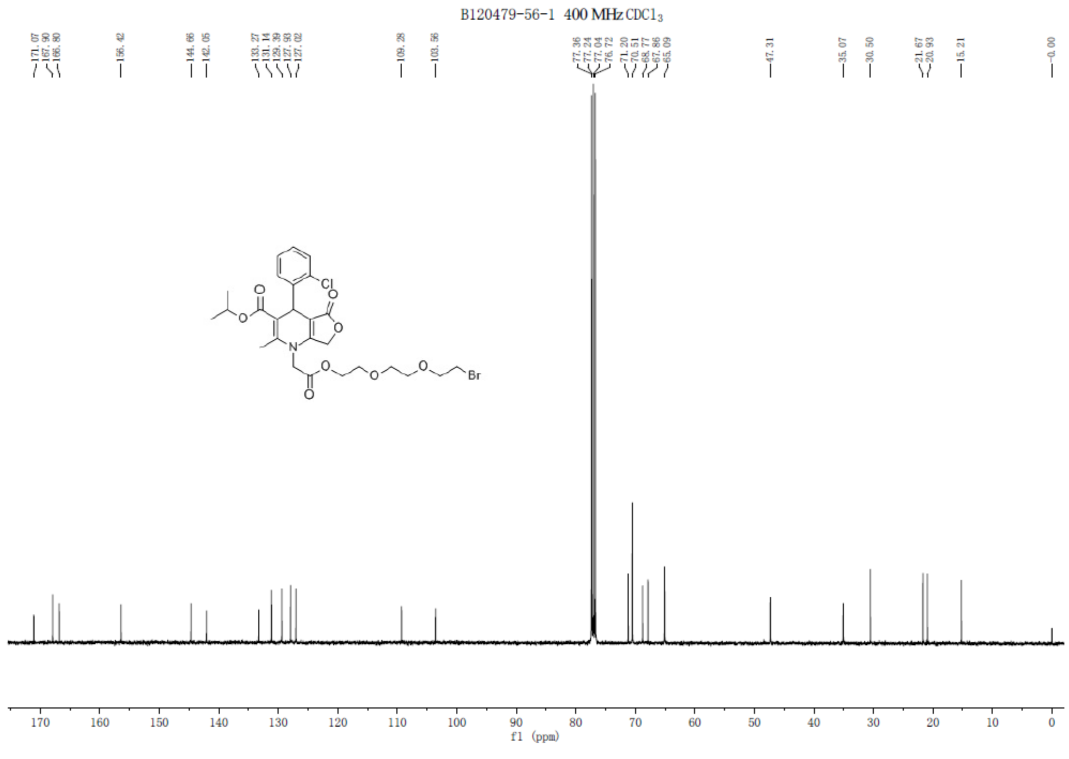

^{1}H NMR spectra of compound **13d**

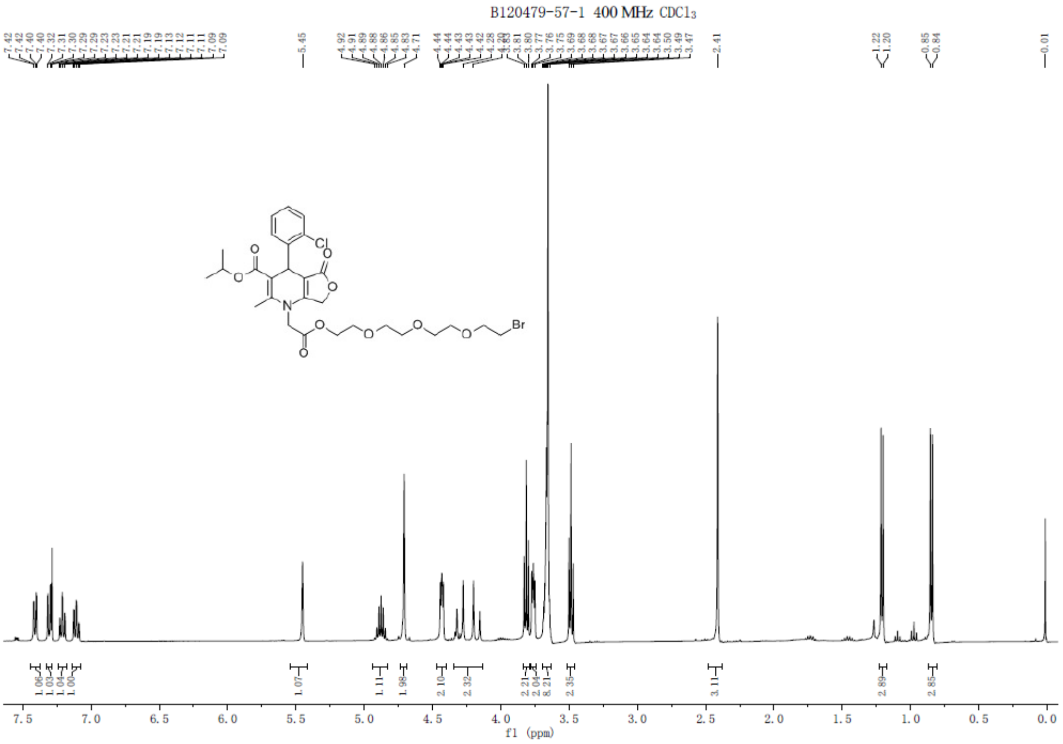

^{13}C NMR spectra of compound **13d**

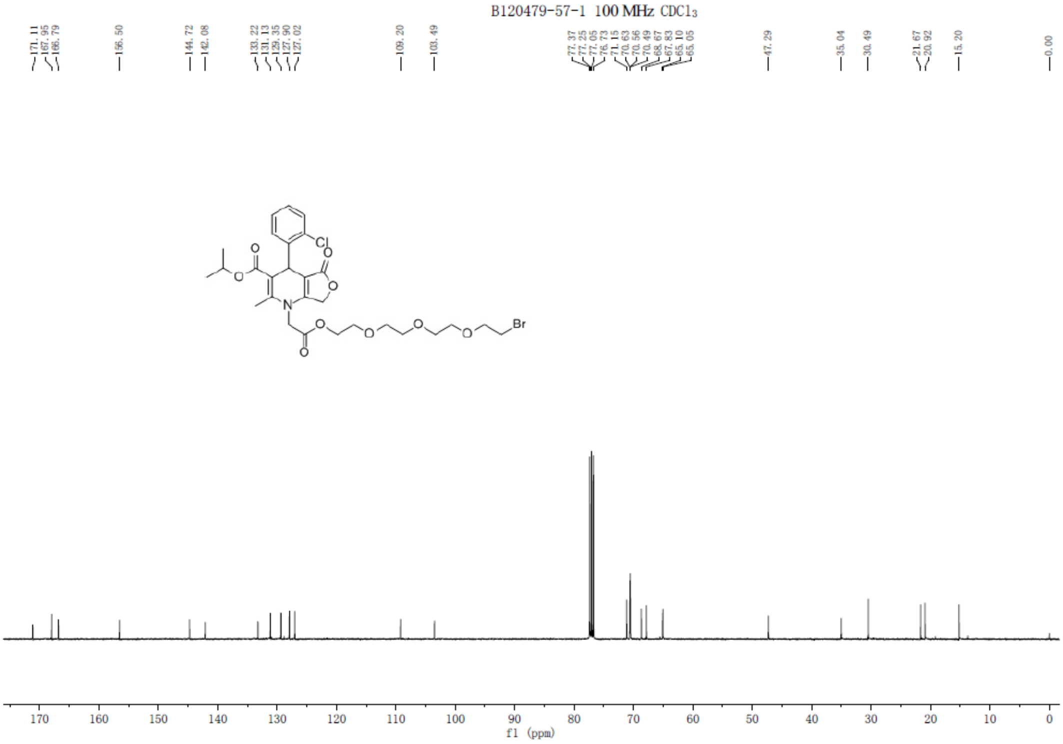

^{1}H NMR spectra of compound **6a**

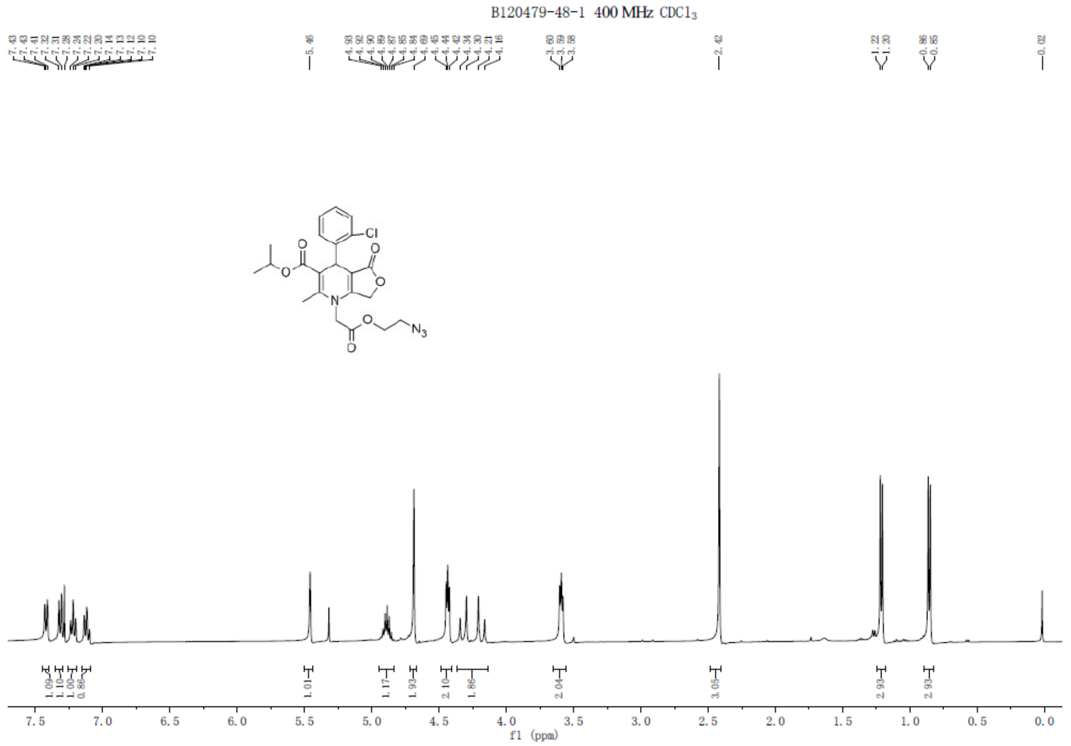

^{13}C NMR spectra of compound **6a**

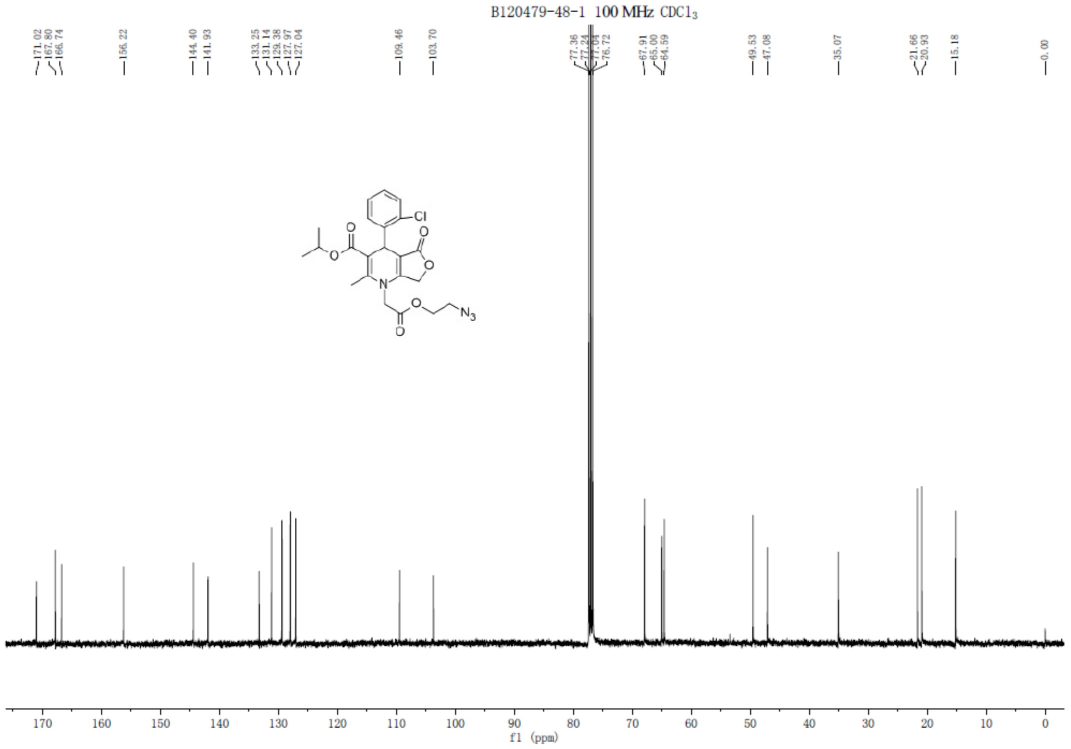

^{1}H NMR spectra of compound **6b**

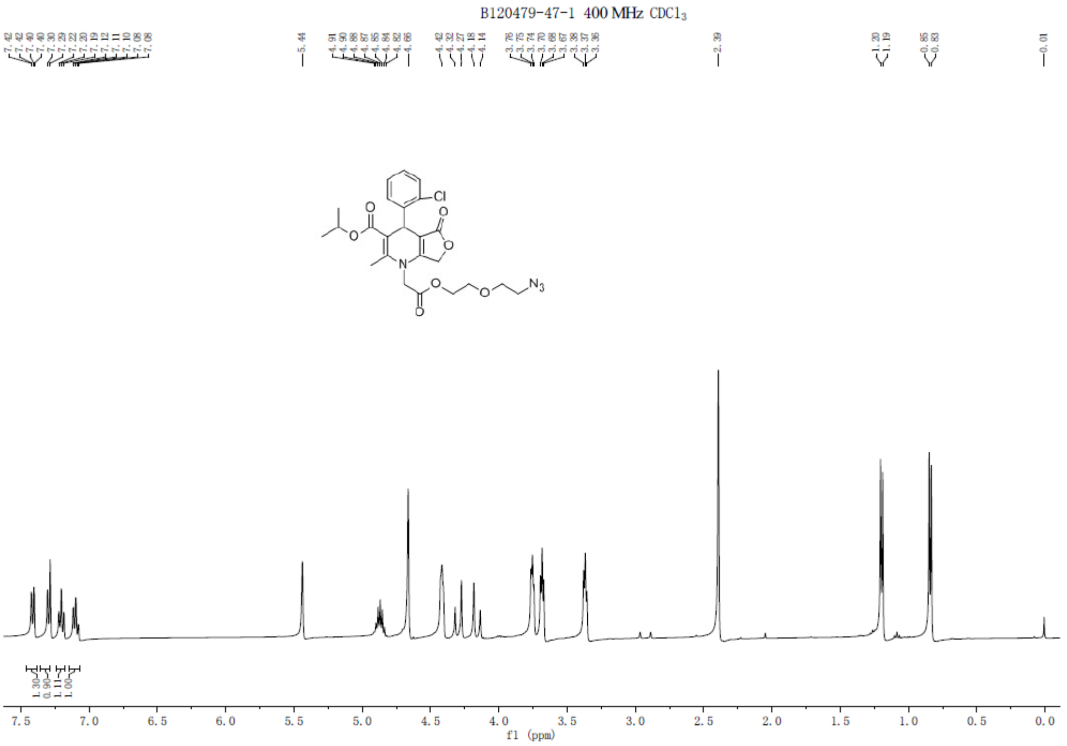

^{13}C NMR spectra of compound **6b**

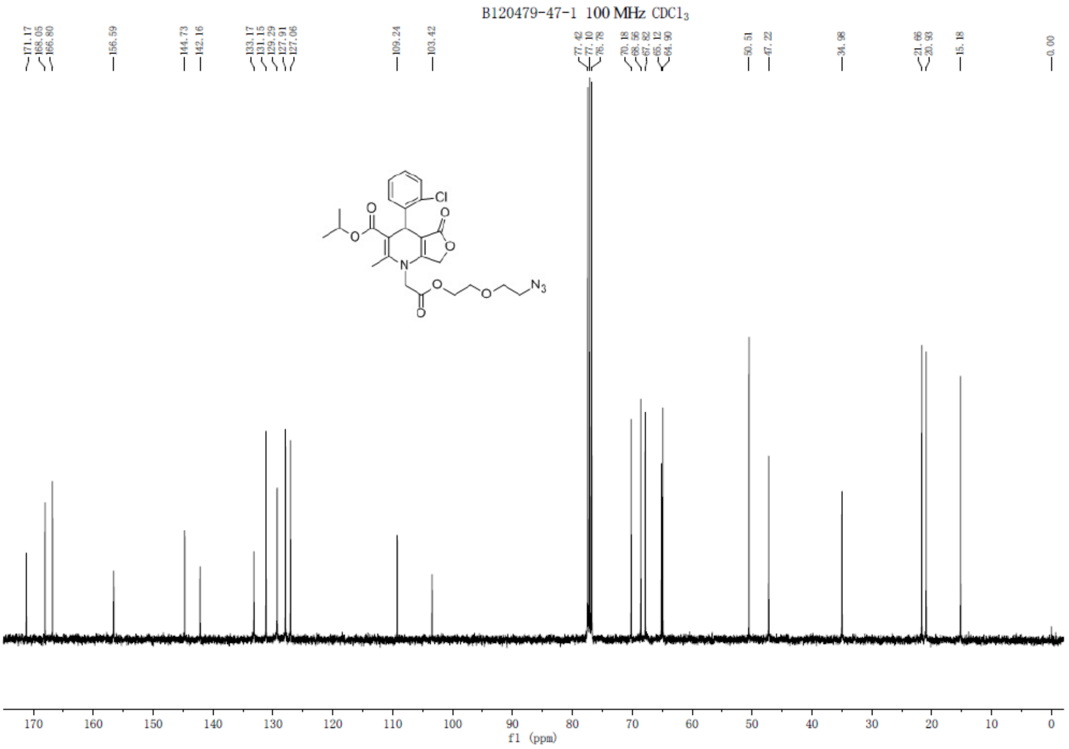

^{1}H NMR spectra of compound **6c**

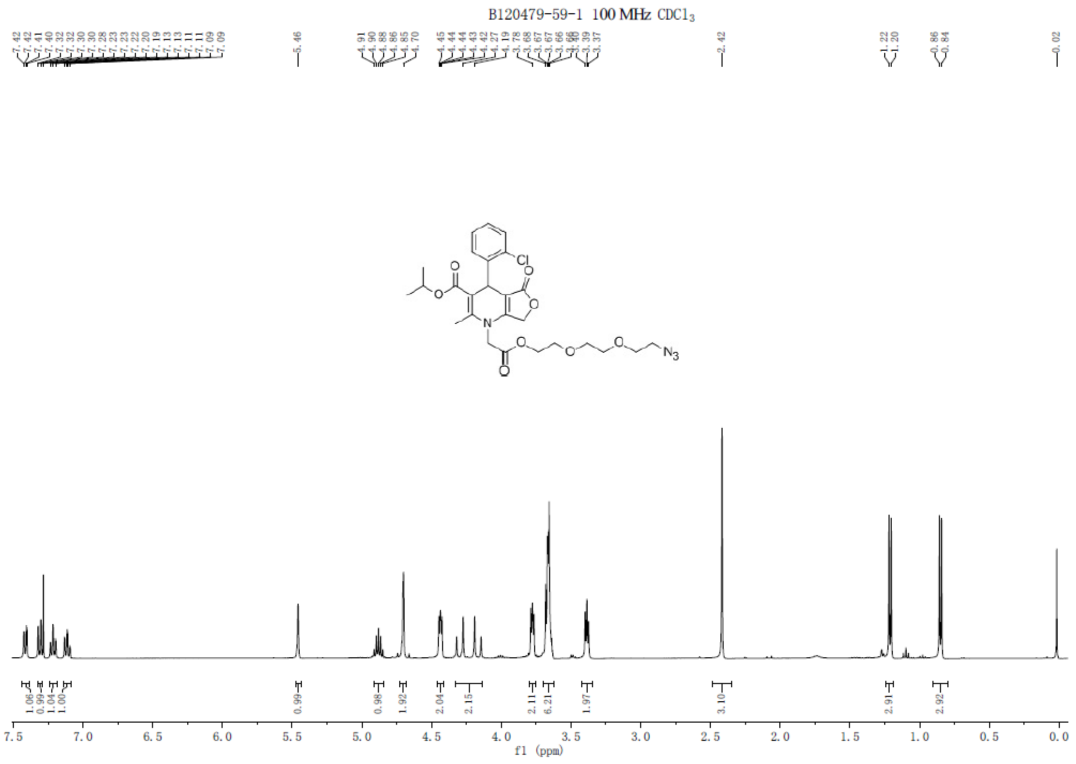

^{13}C NMR spectra of compound **6c**

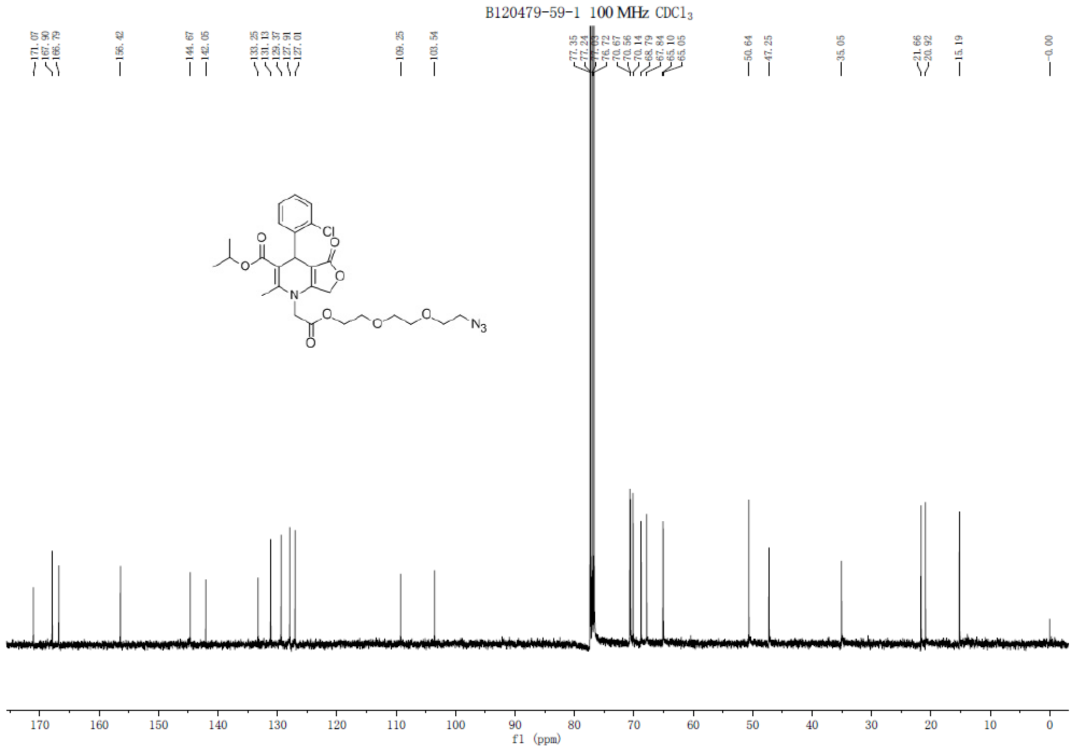

^{1}H NMR spectra of compound **6d**

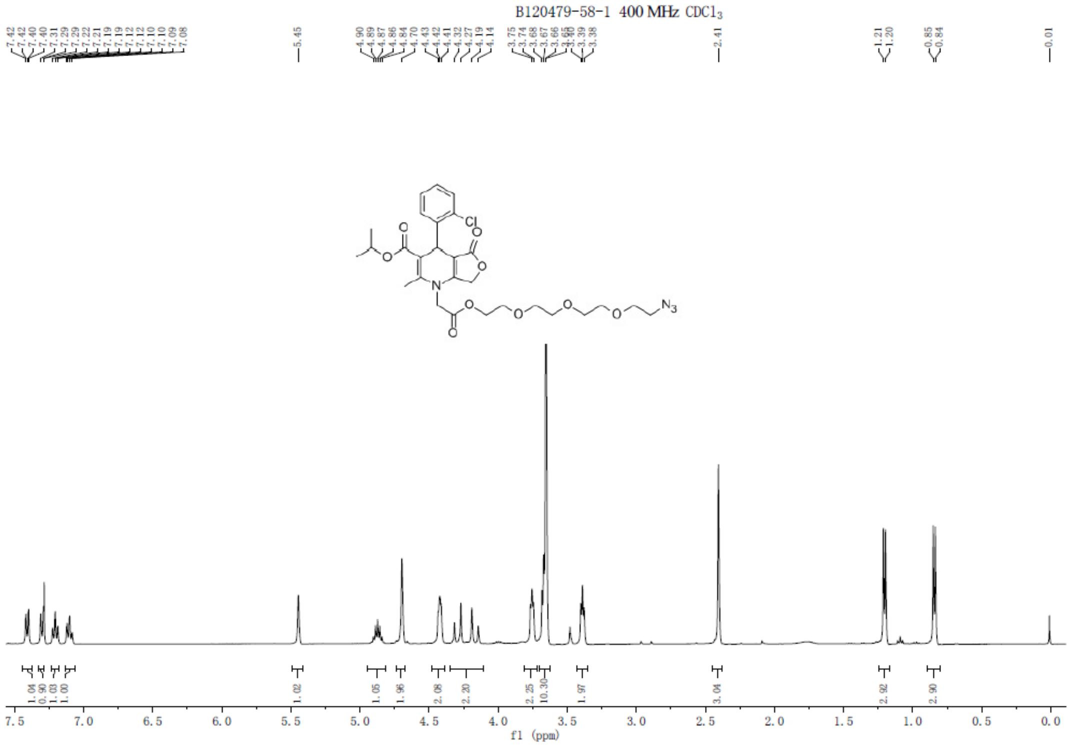

^{13}C NMR spectra of compound **6d**

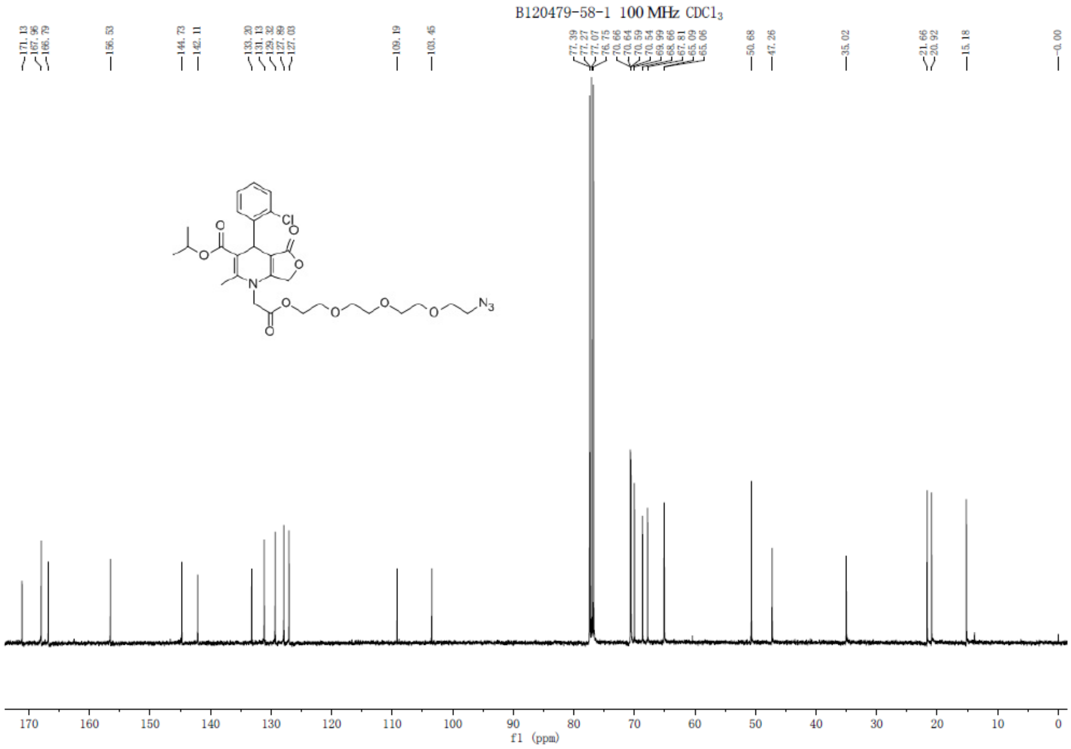

^{1}H NMR spectra of compound **16**

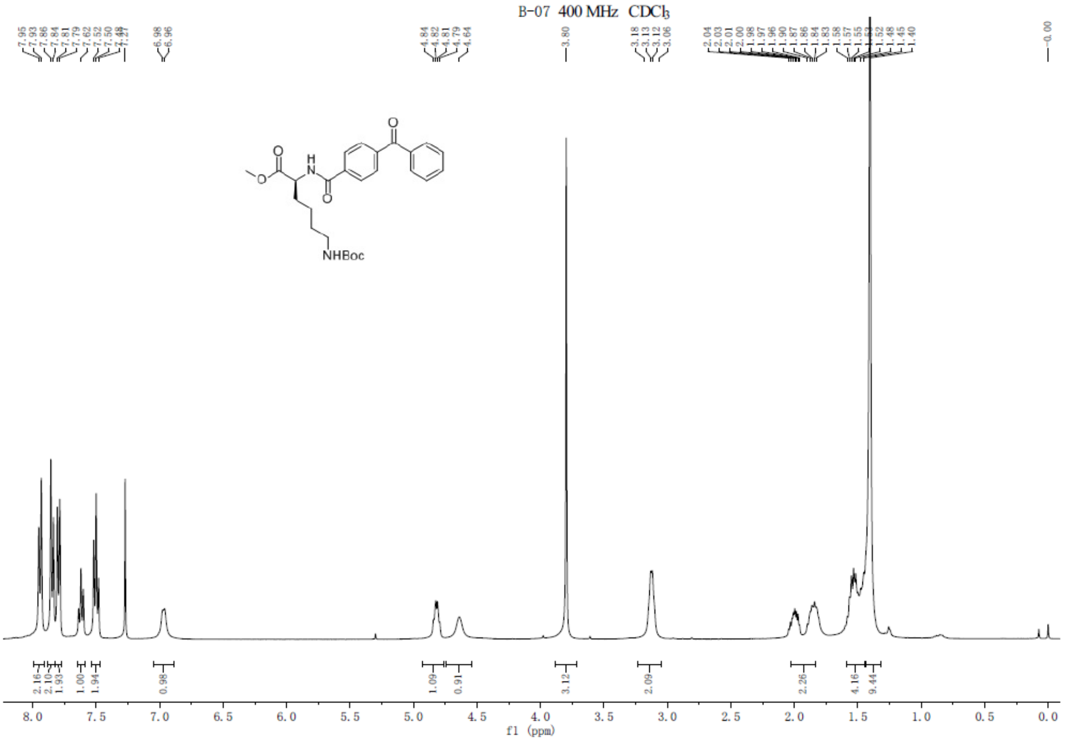

^{13}C NMR spectra of compound **16**

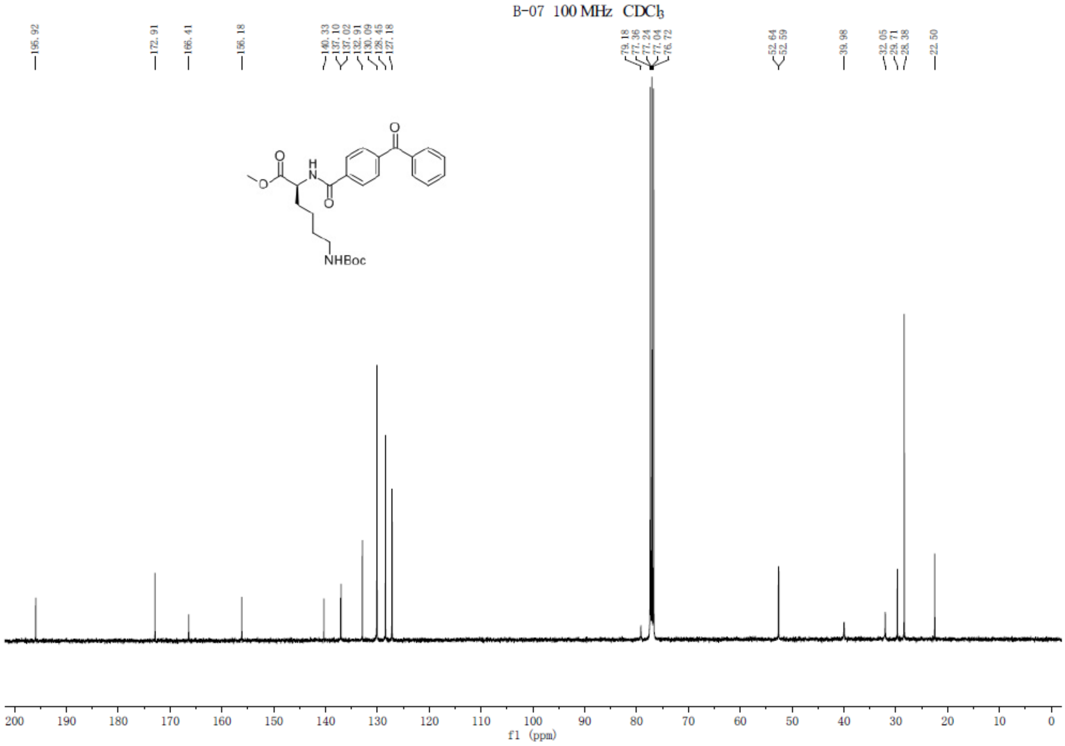

^{1}H NMR spectra of compound **18**

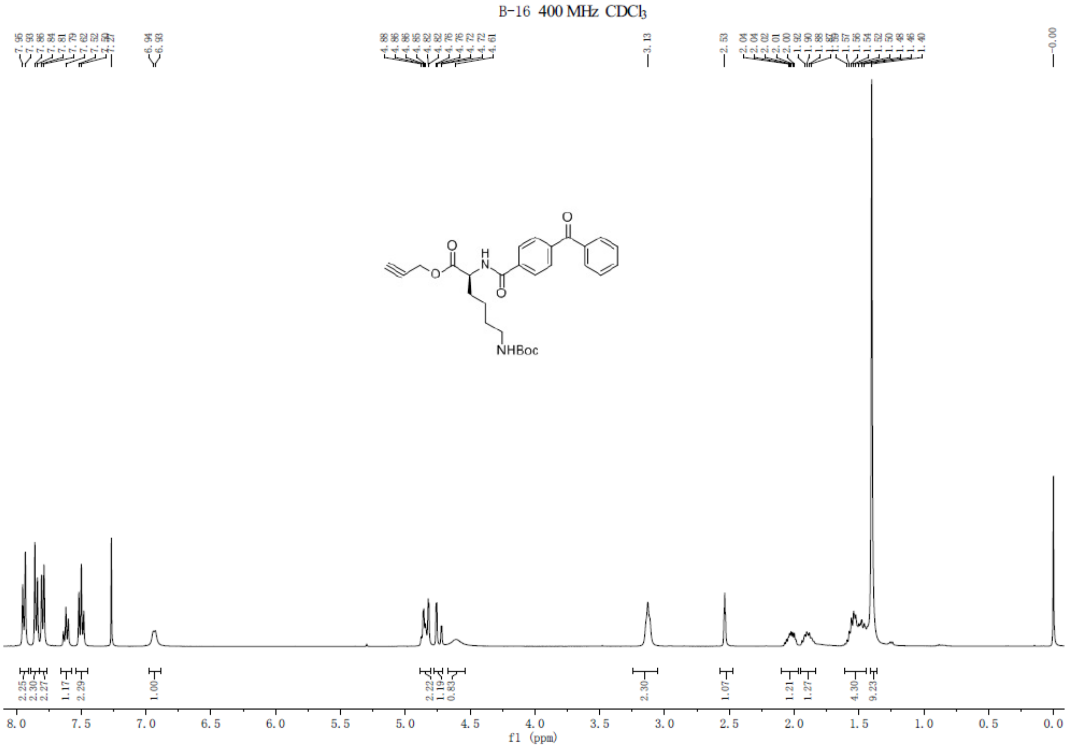

^{13}C NMR spectra of compound **18**

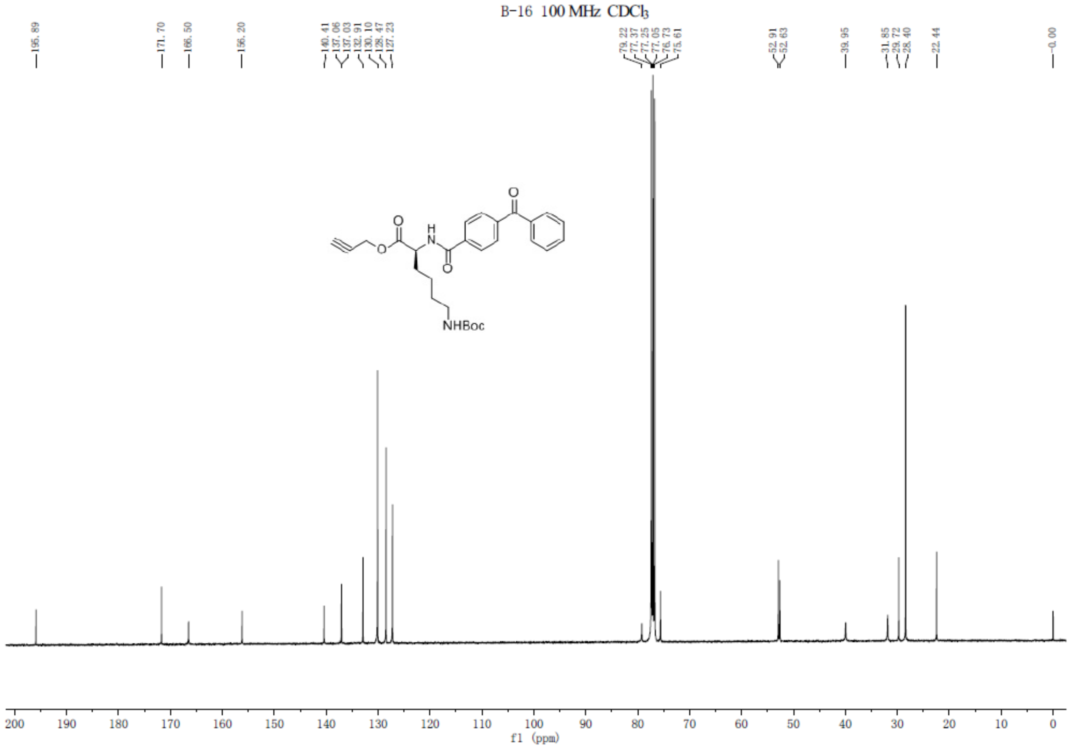

^{1}H NMR spectra of compound **20**

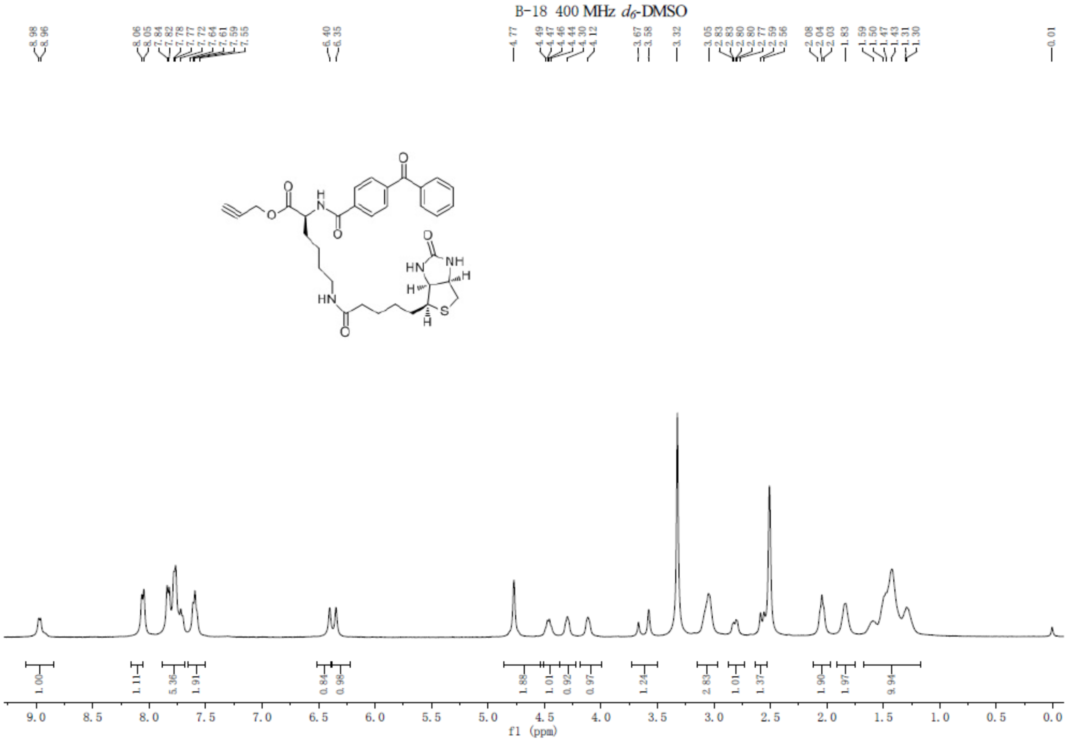

^{13}C NMR spectra of compound **20**

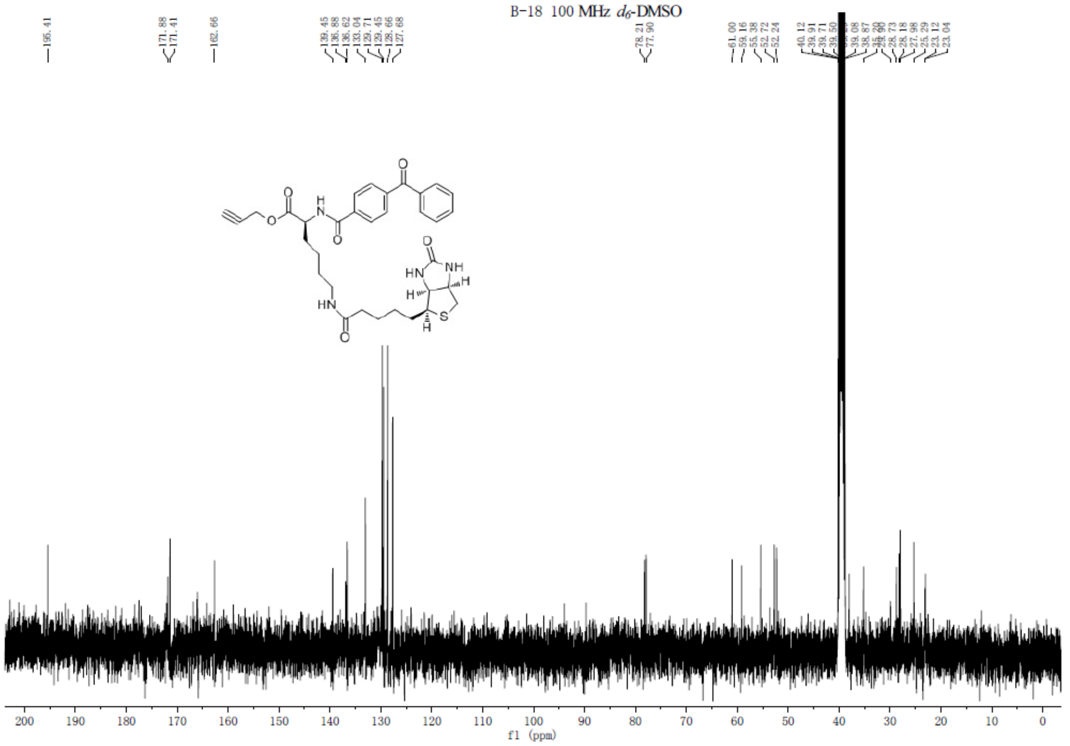

^{1}H NMR spectra of compound **21**

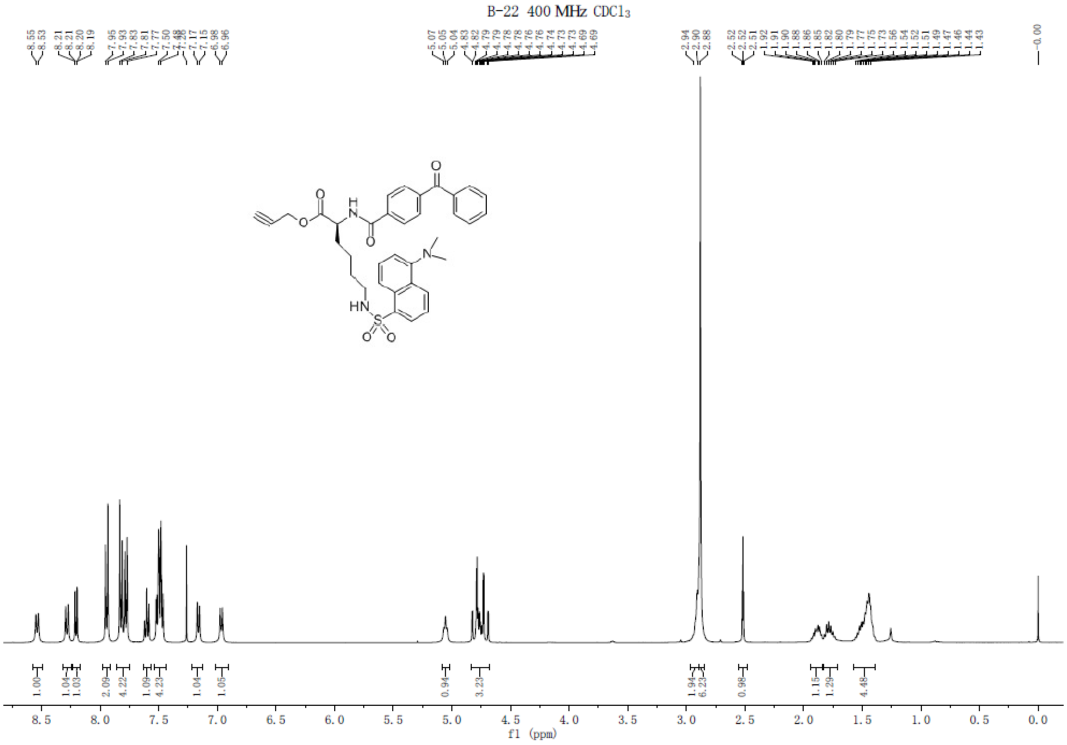

^{13}C NMR spectra of compound **21**

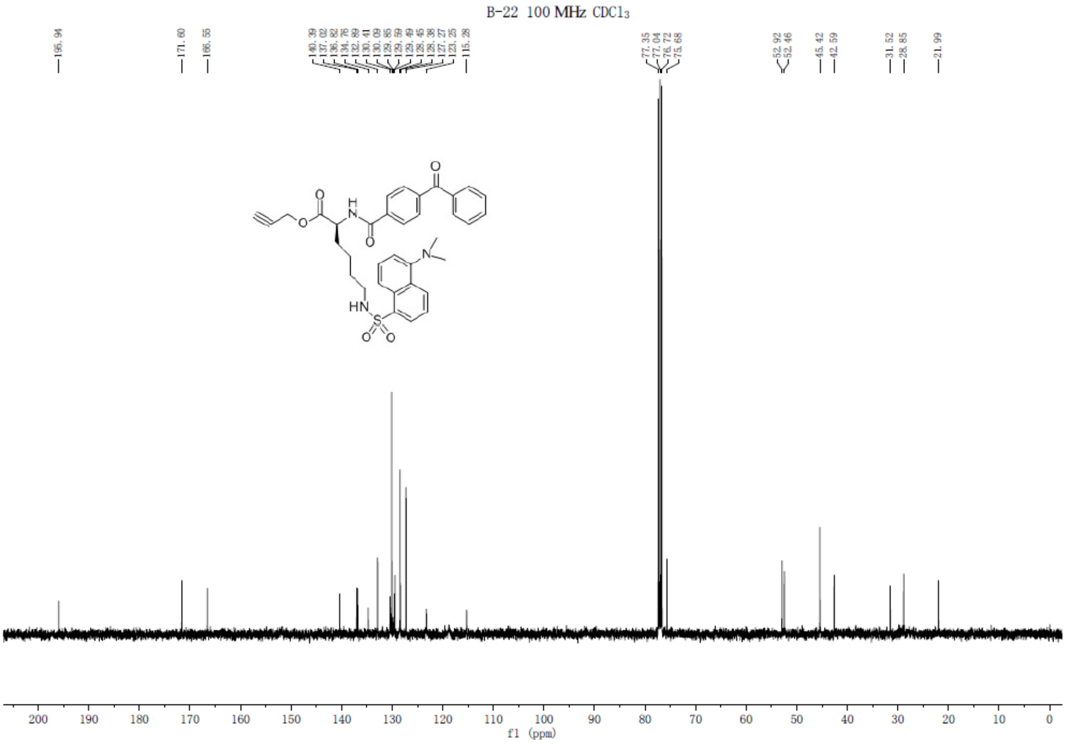

^{1}H NMR spectra of compound**22**

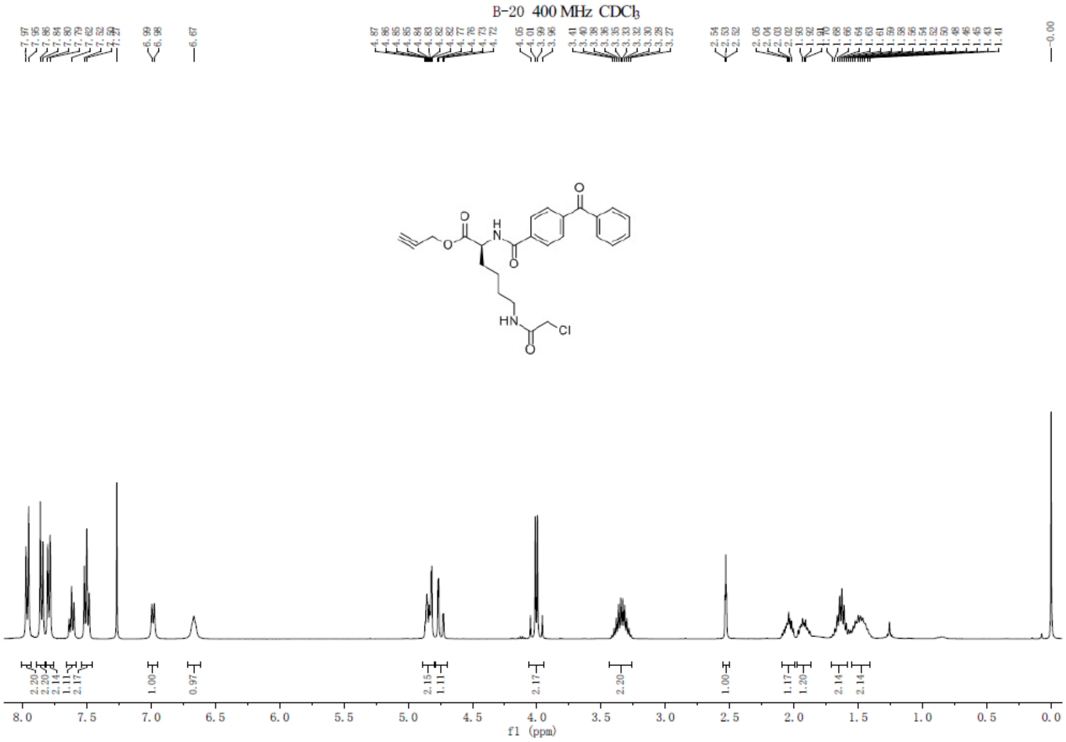

^{13}C NMR spectra of compound**22**

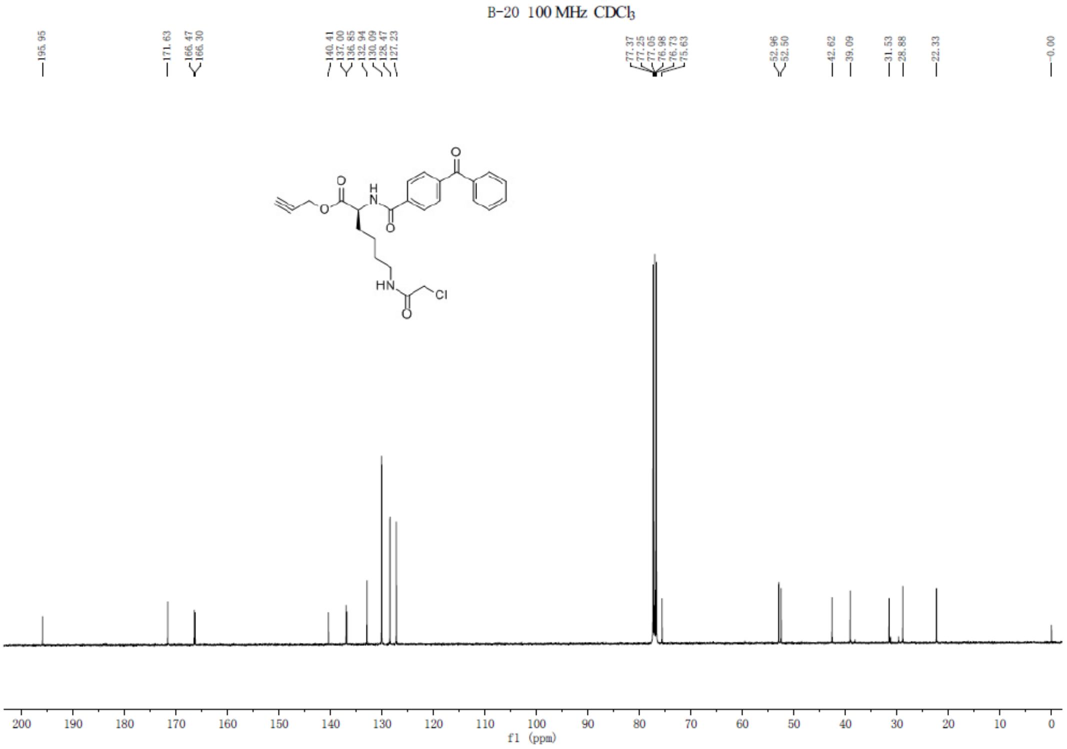

^1H NMR spectra of compound **24**

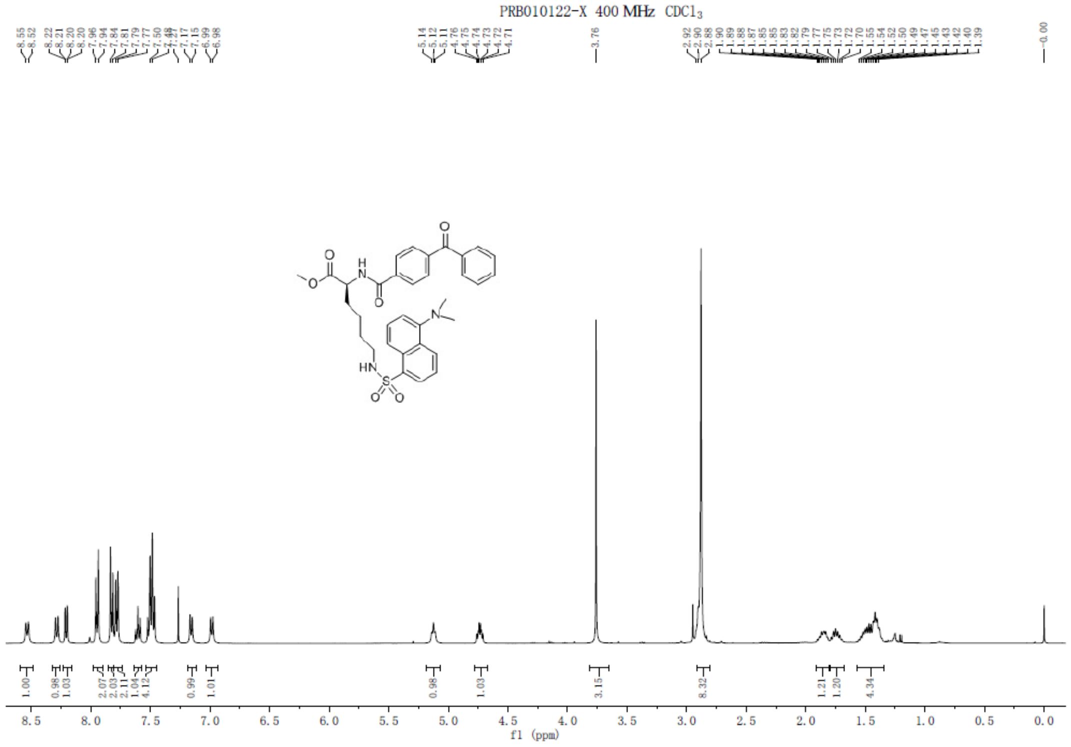

^{13}C NMR spectra of compound **24**

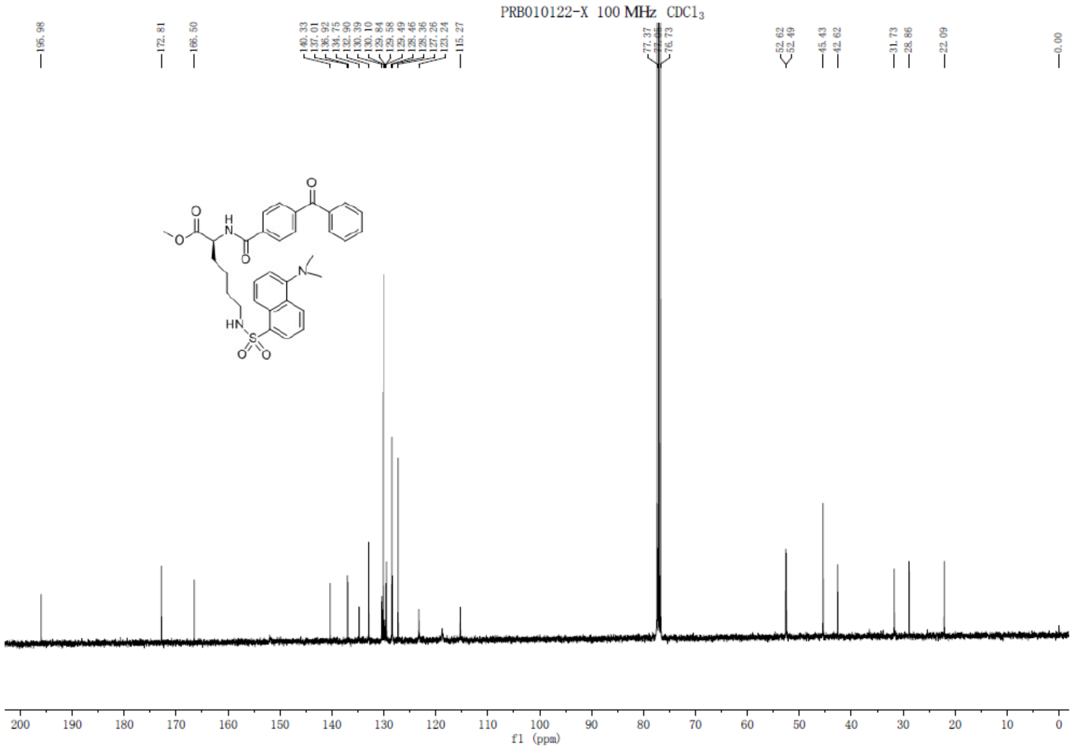

^{1}H NMR spectra of compound **25**

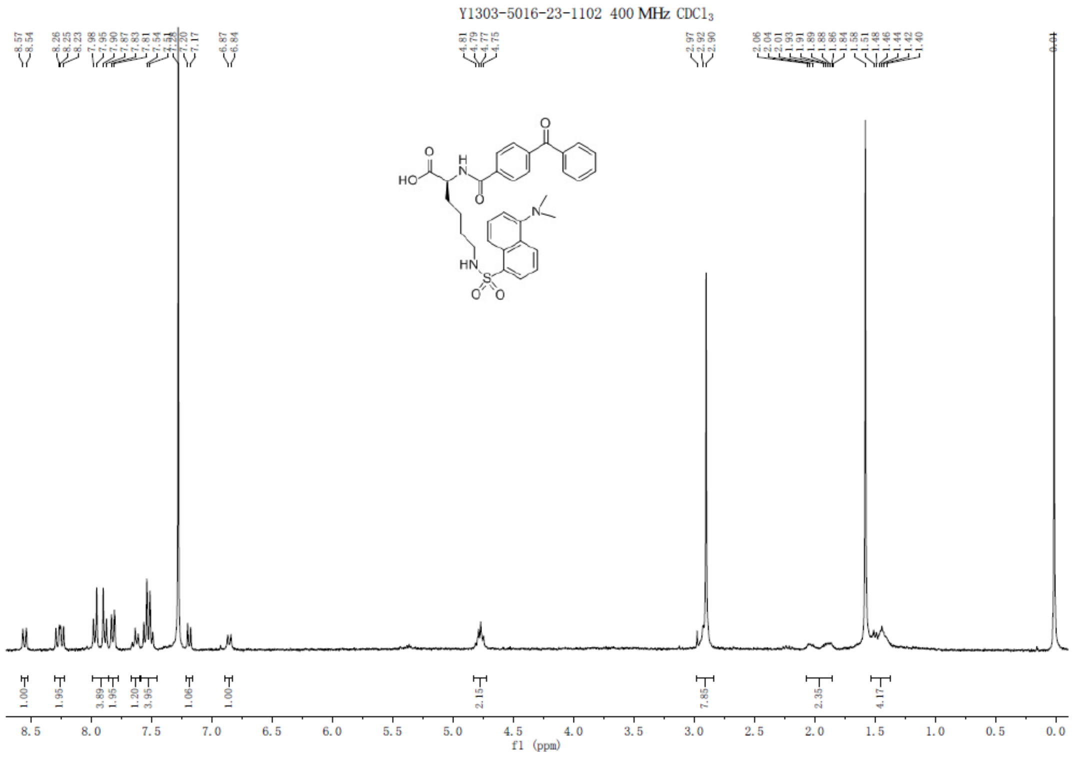

^{13}C NMR spectra of compound **25**

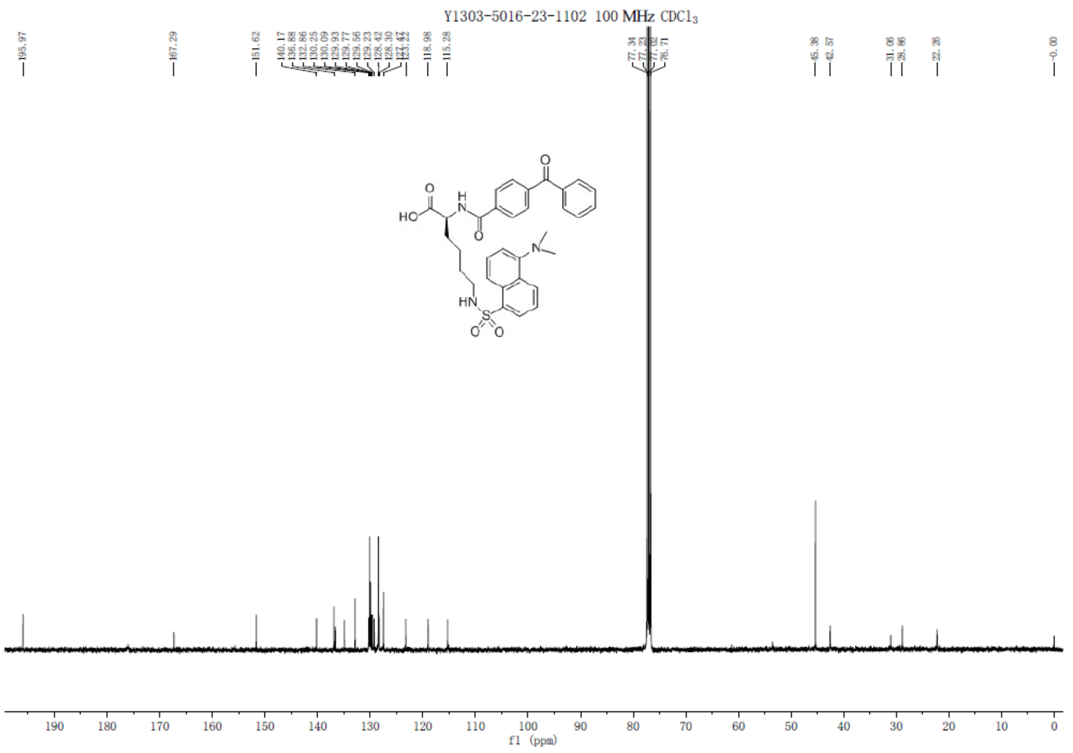

^{1}H NMR spectra of compound **27**

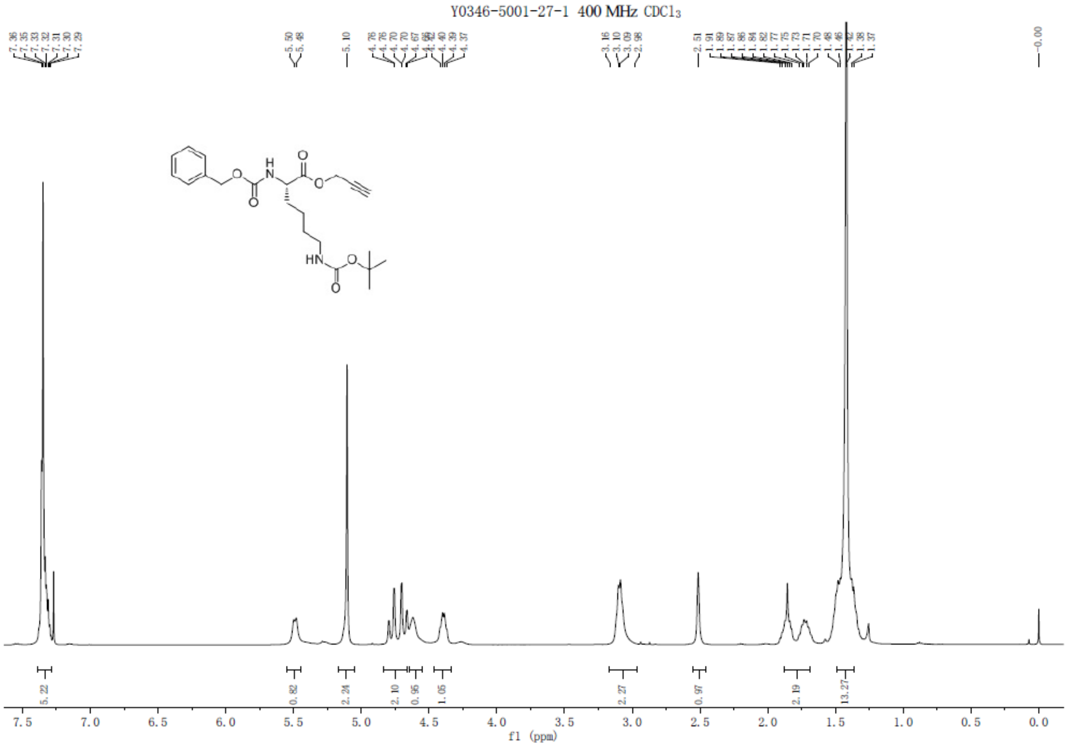

^{13}C NMR spectra of compound **27**

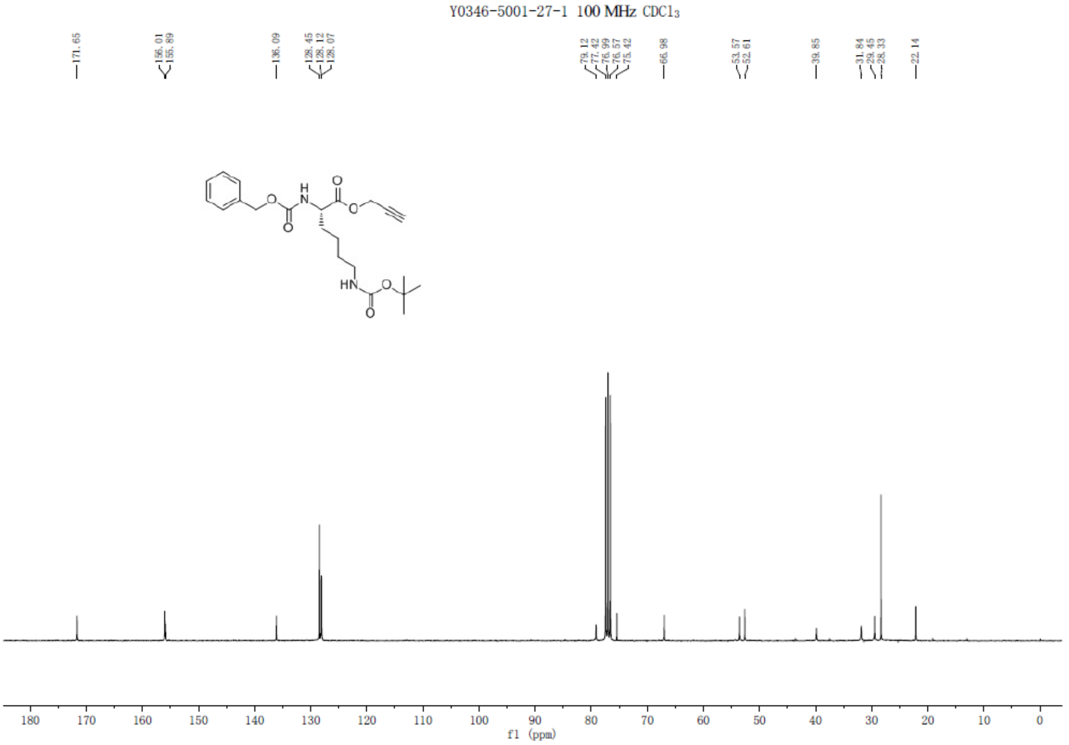

^{1}H NMR spectra of compound **28**

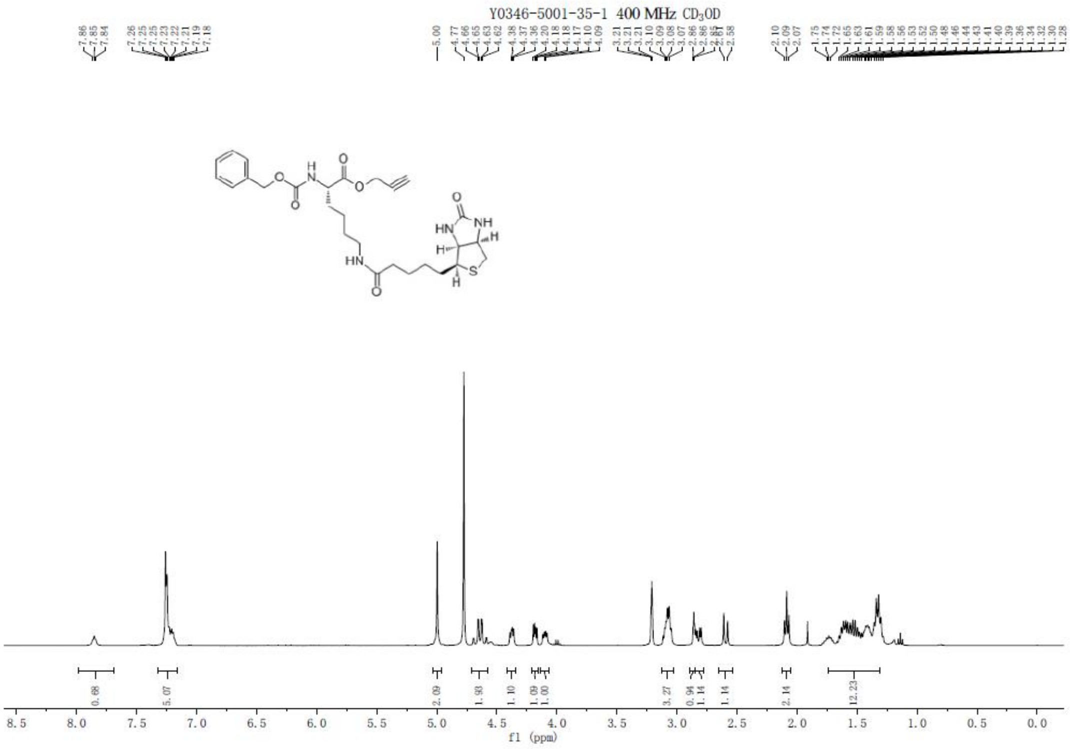

^{13}C NMR spectra of compound **28**

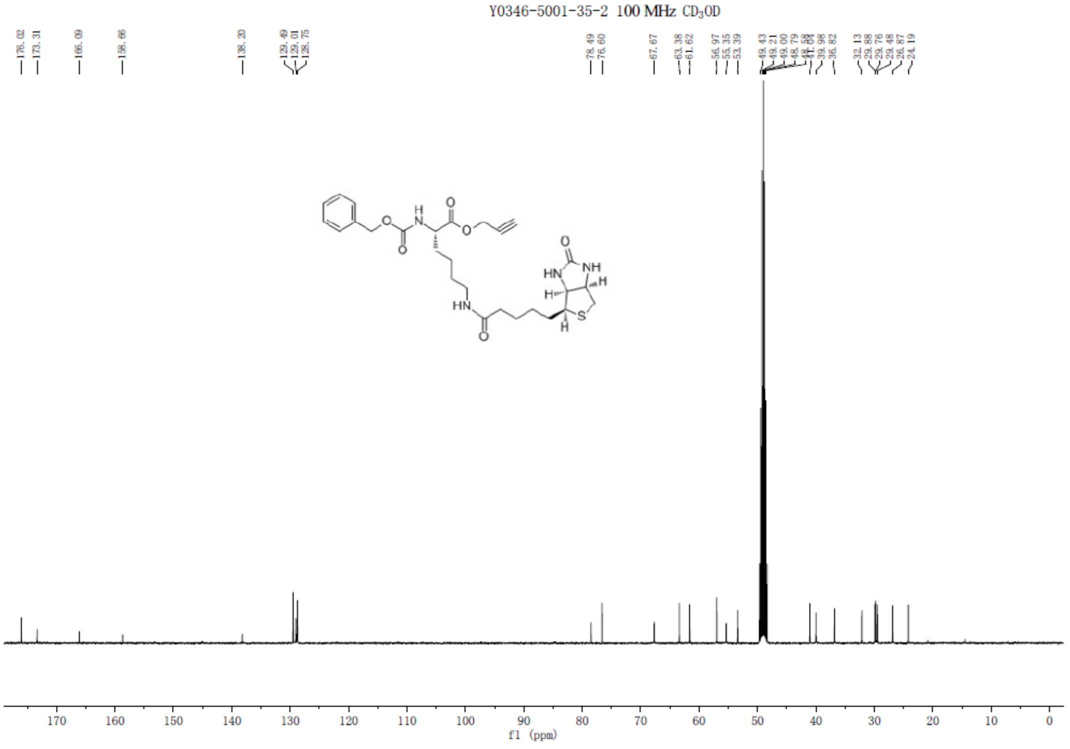

^{1}H NMR spectra of compound **30**

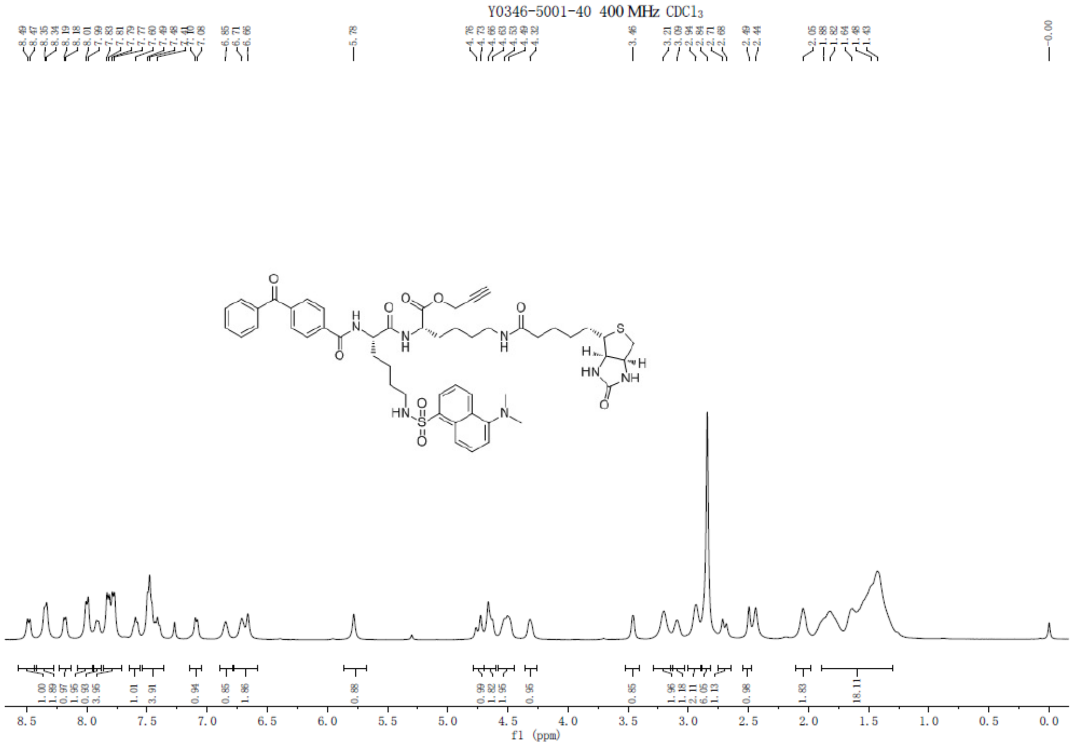

^{13}C NMR spectra of compound **30**

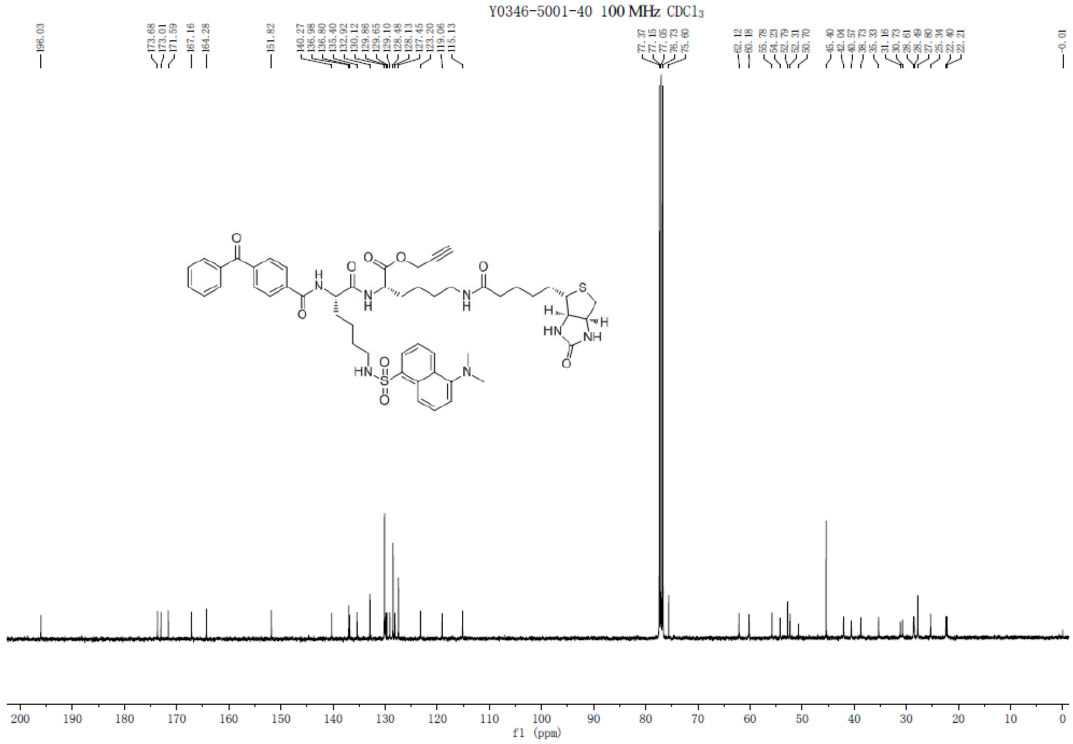

^{1}H NMR spectra of compound **2a**

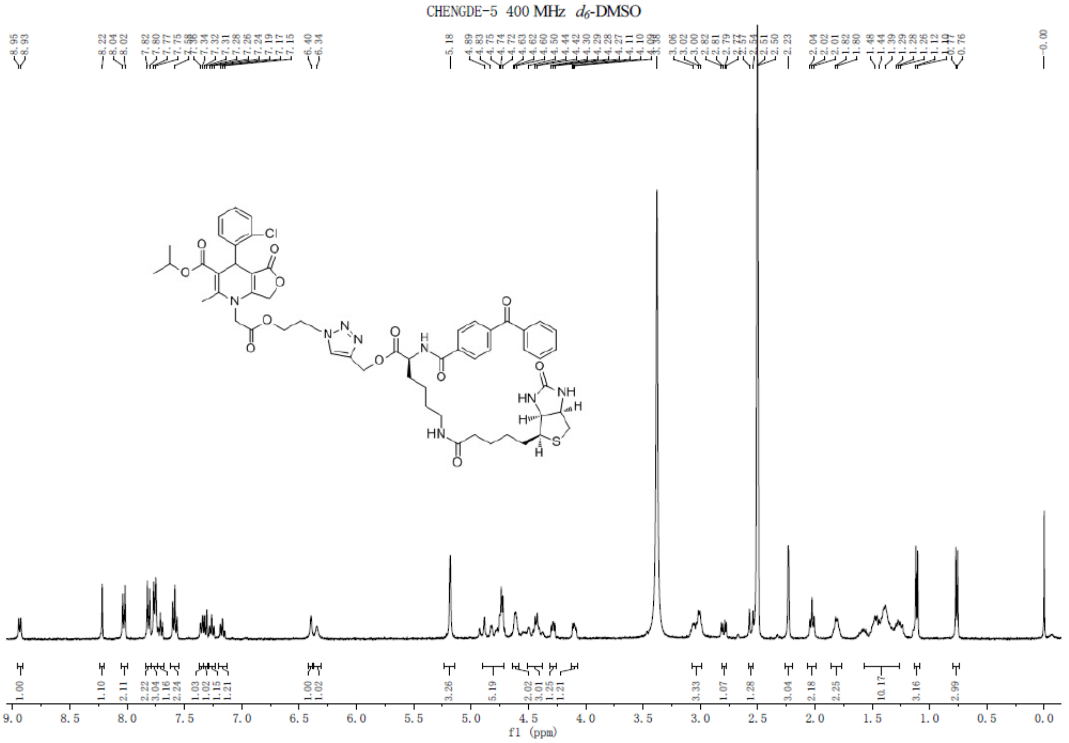

^{13}C NMR spectra of compound **2a**

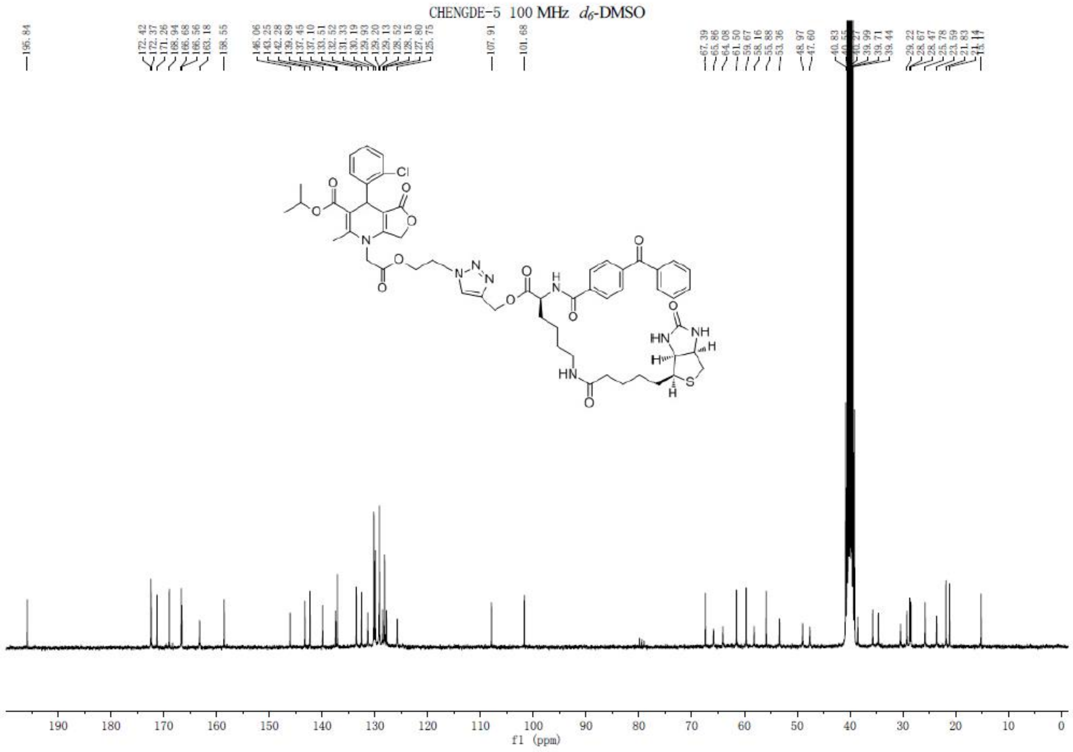

^{1}H NMR spectra of compound **2b**

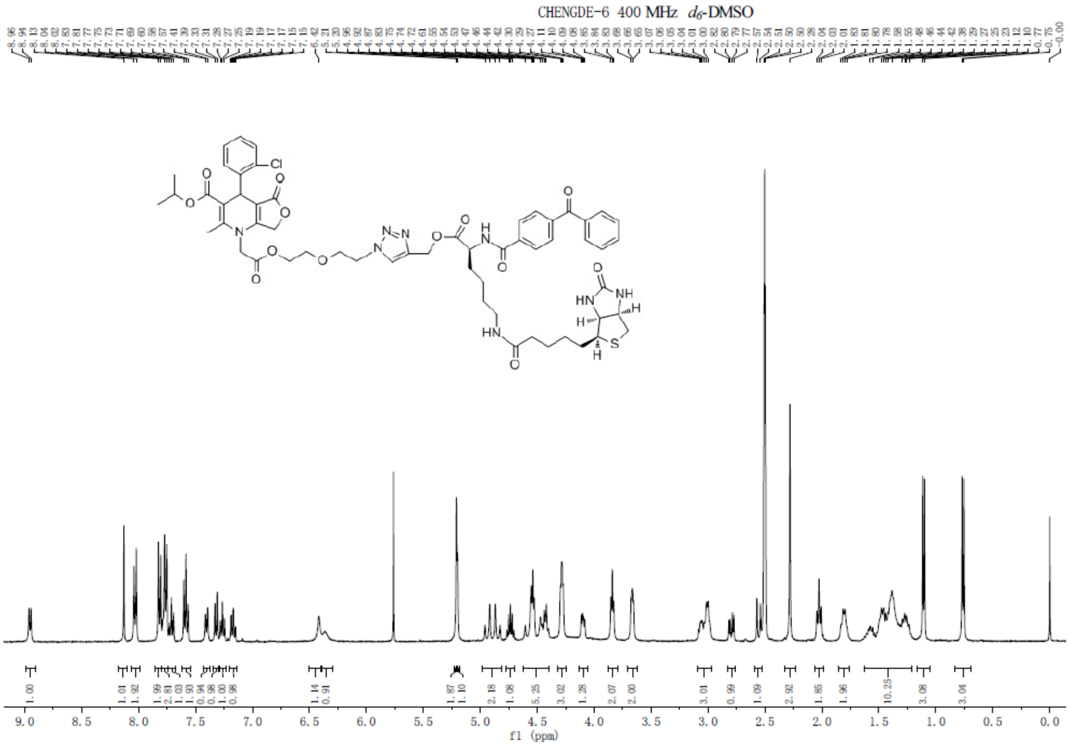

^{13}C NMR spectra of compound **2b**

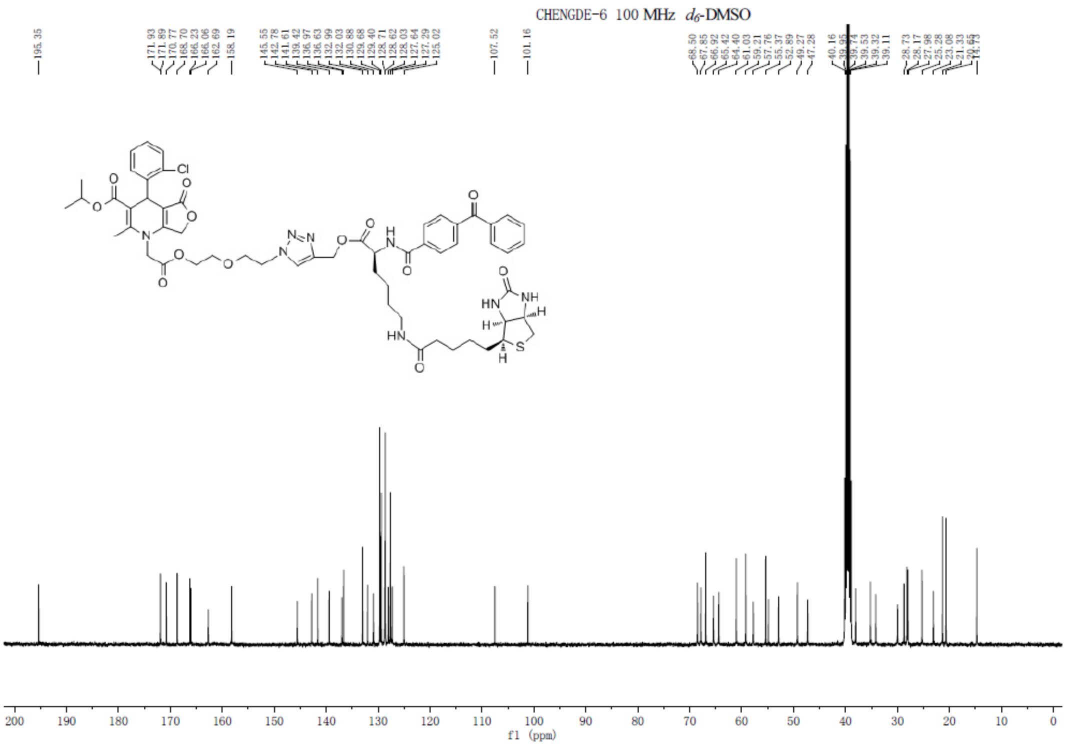

^{1}H NMR spectra of compound **2c**

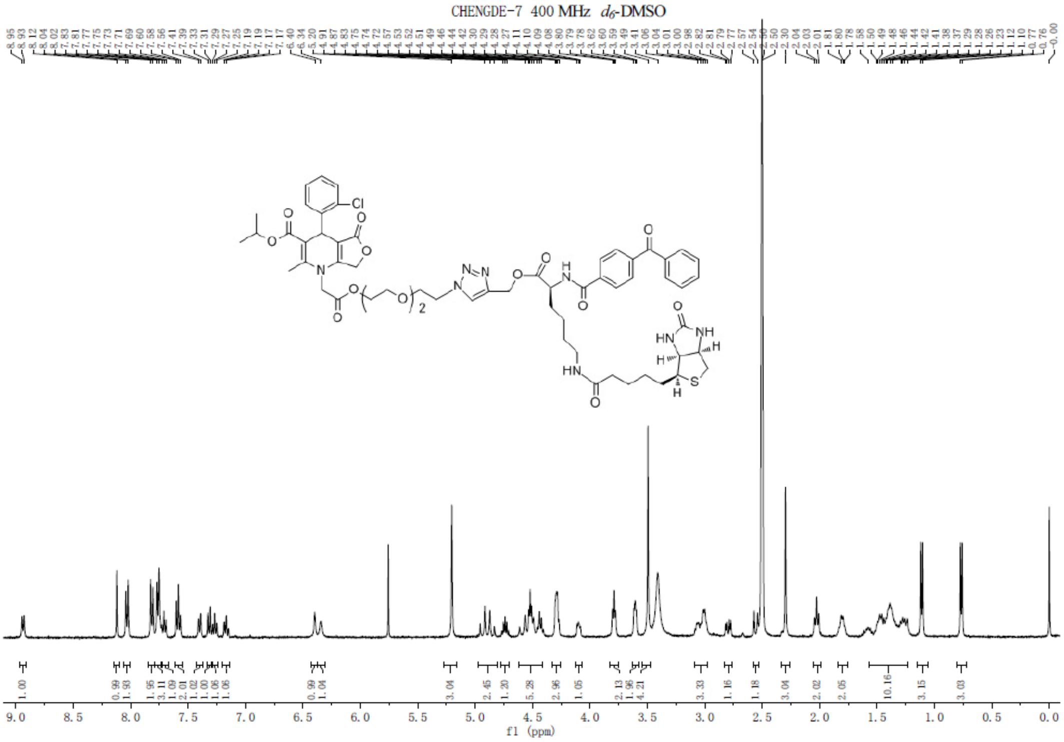

^{13}C NMR spectra of compound **2c**

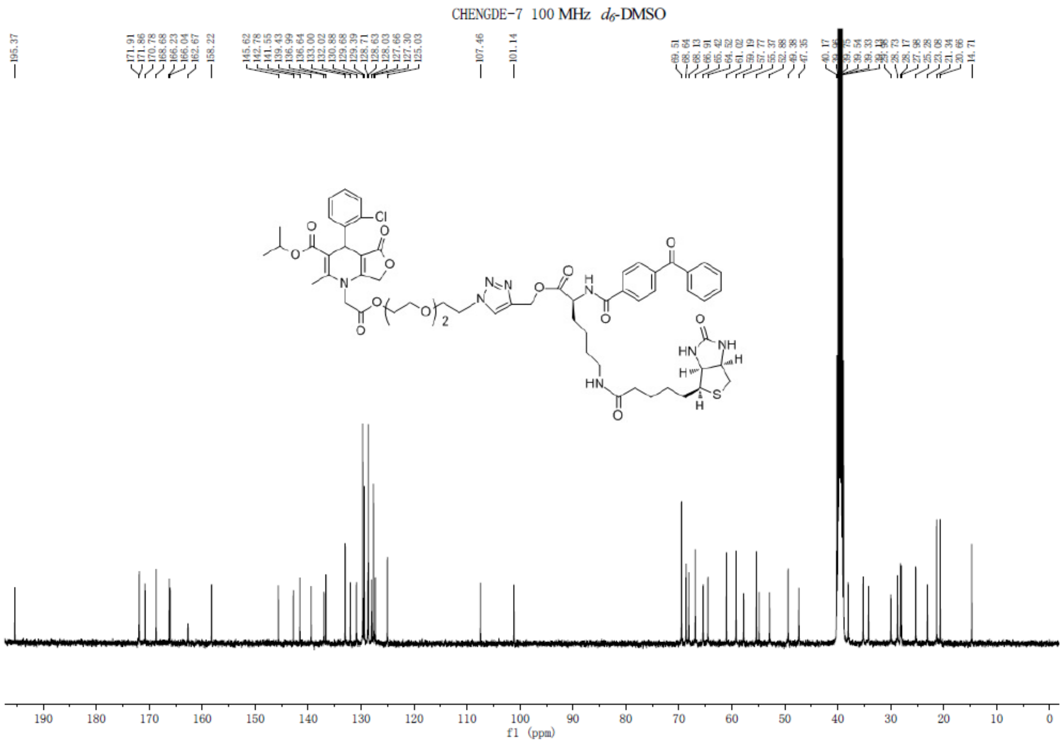

^{1}H NMR spectra of compound **2d**

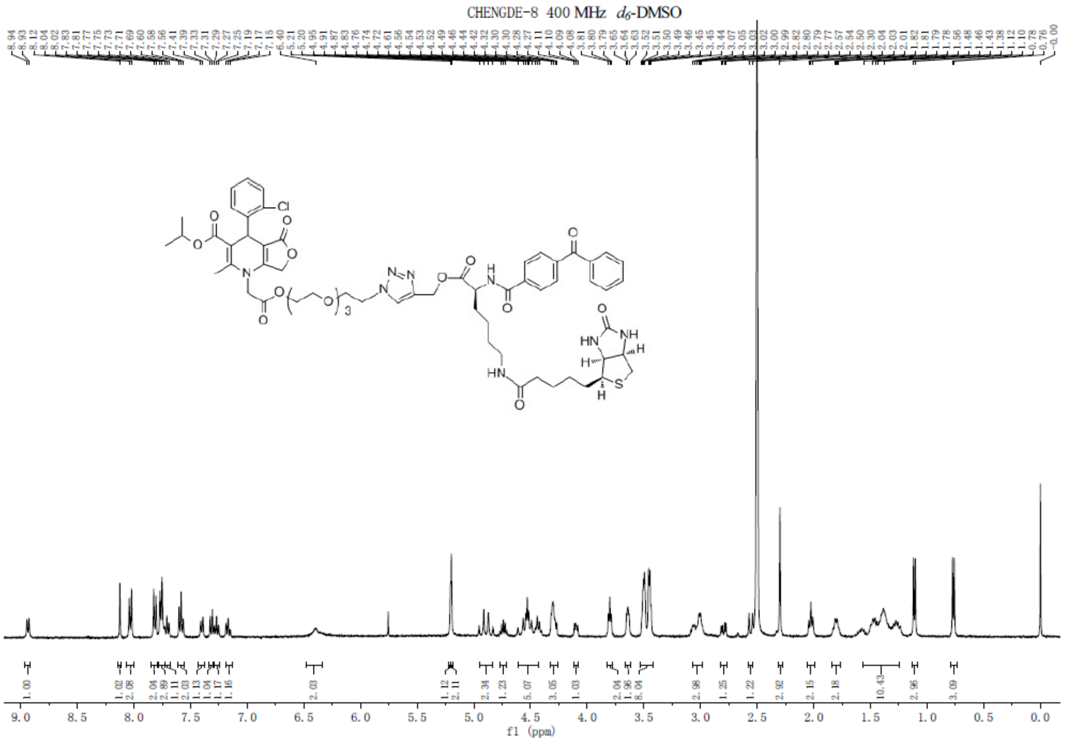

^{13}C NMR spectra of compound **2d**

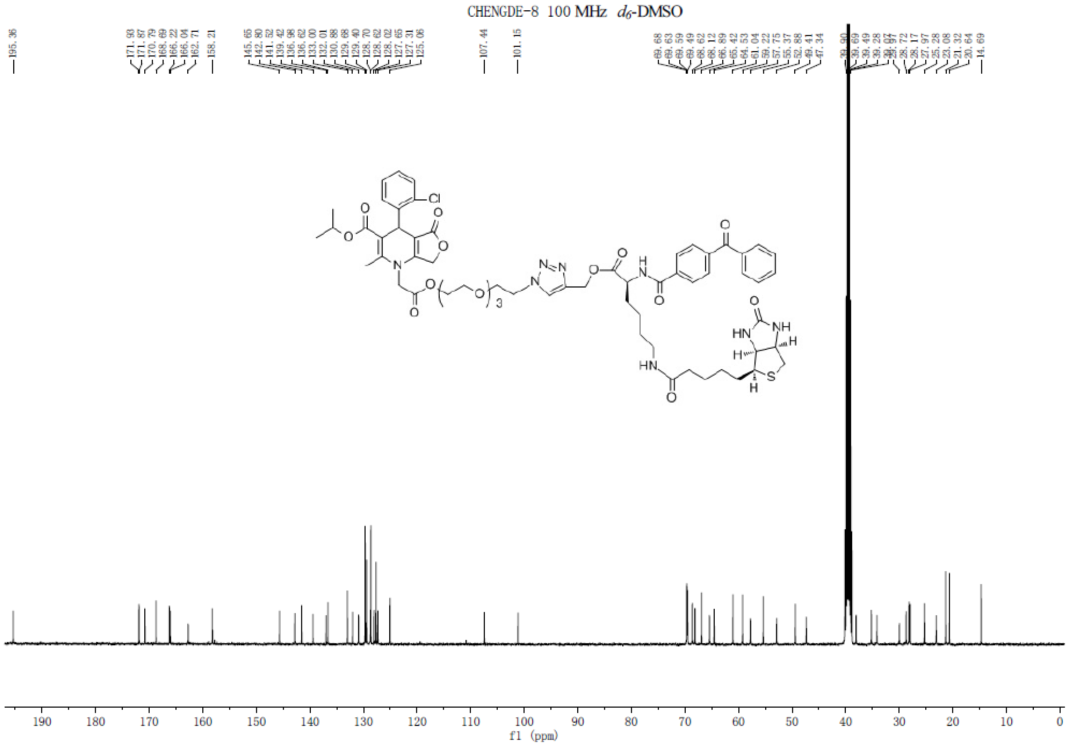

^{1}H NMR spectra of compound **3a**

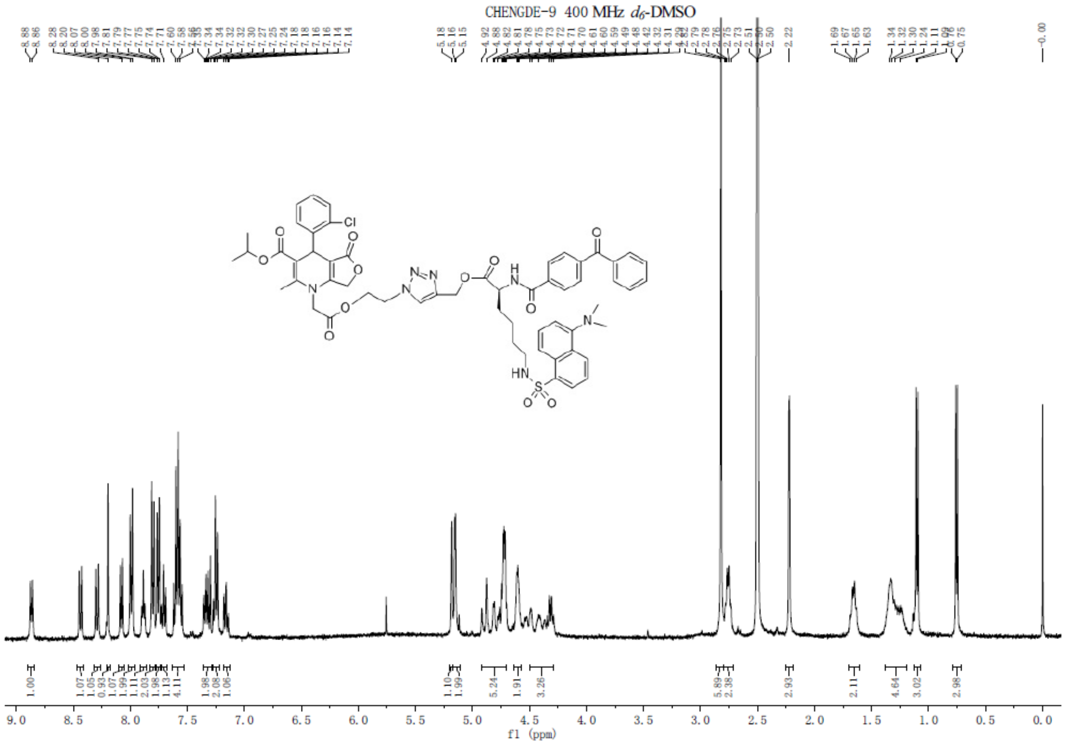

^{13}C NMR spectra of compound **3a**

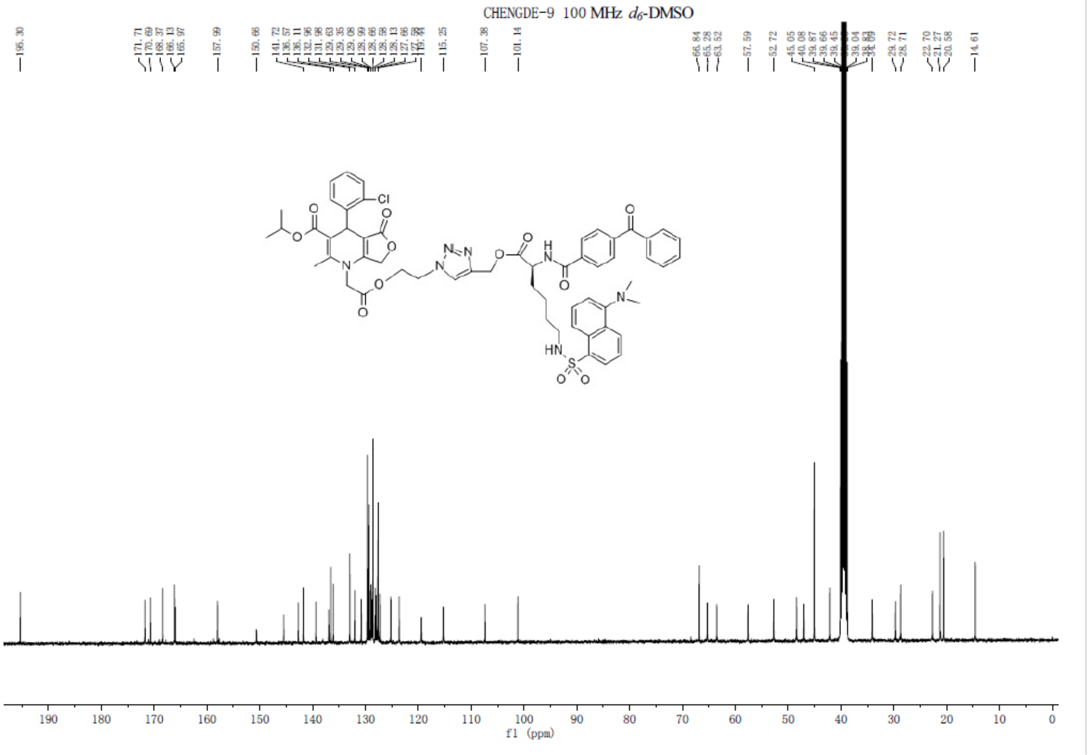

^{1}H NMR spectra of compound **3b**

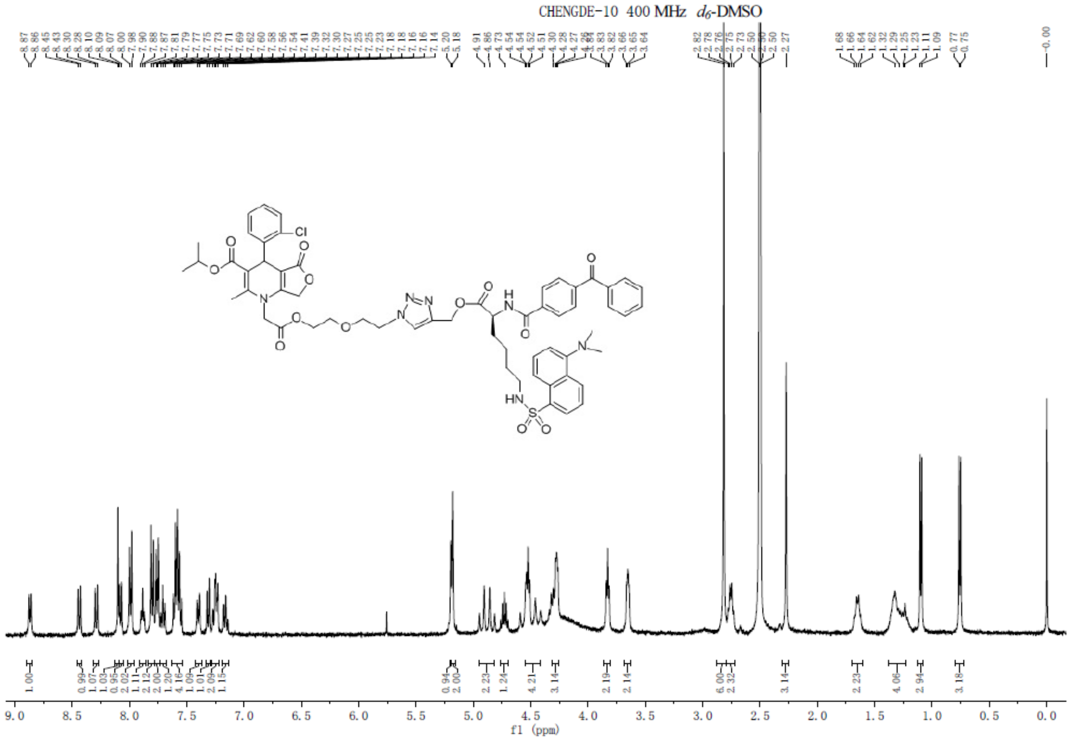

^{13}C NMR spectra of compound **3b**

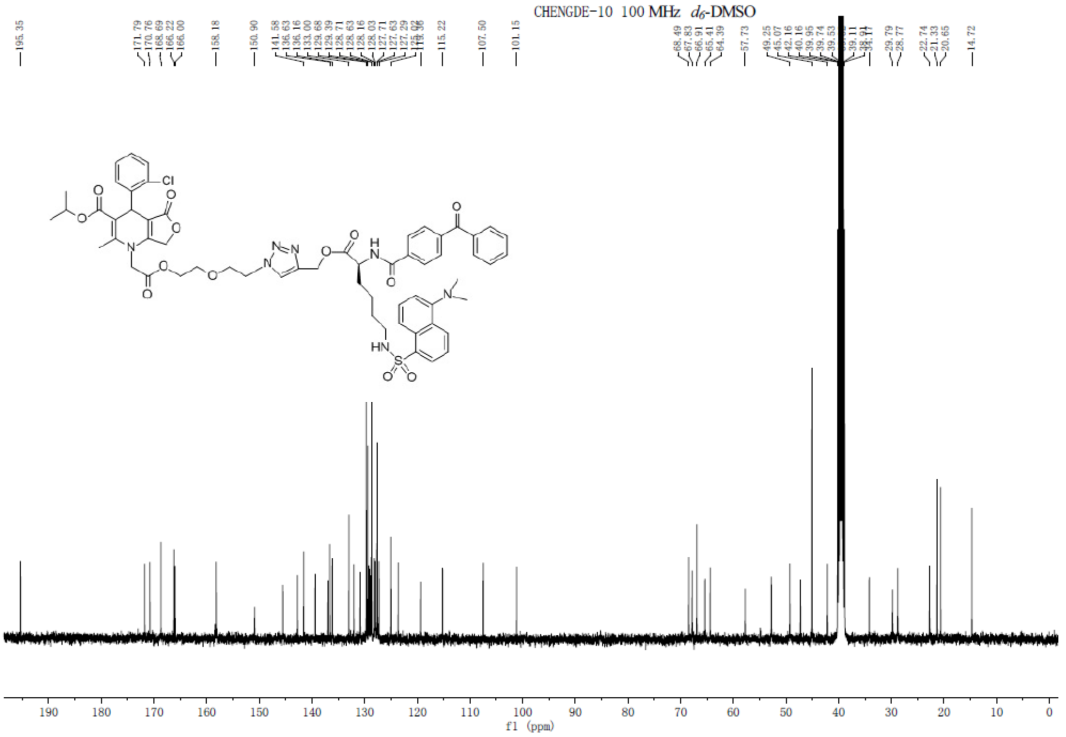

^{1}H NMR spectra of compound **3c**

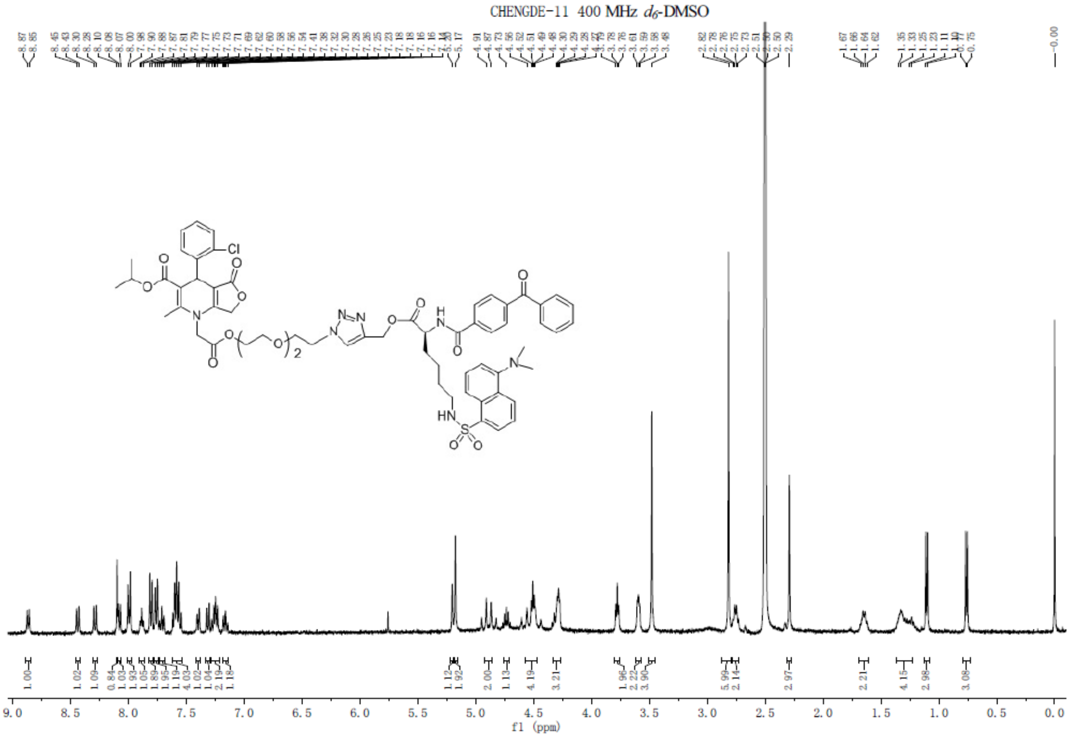

^{13}C NMR spectra of compound **3c**

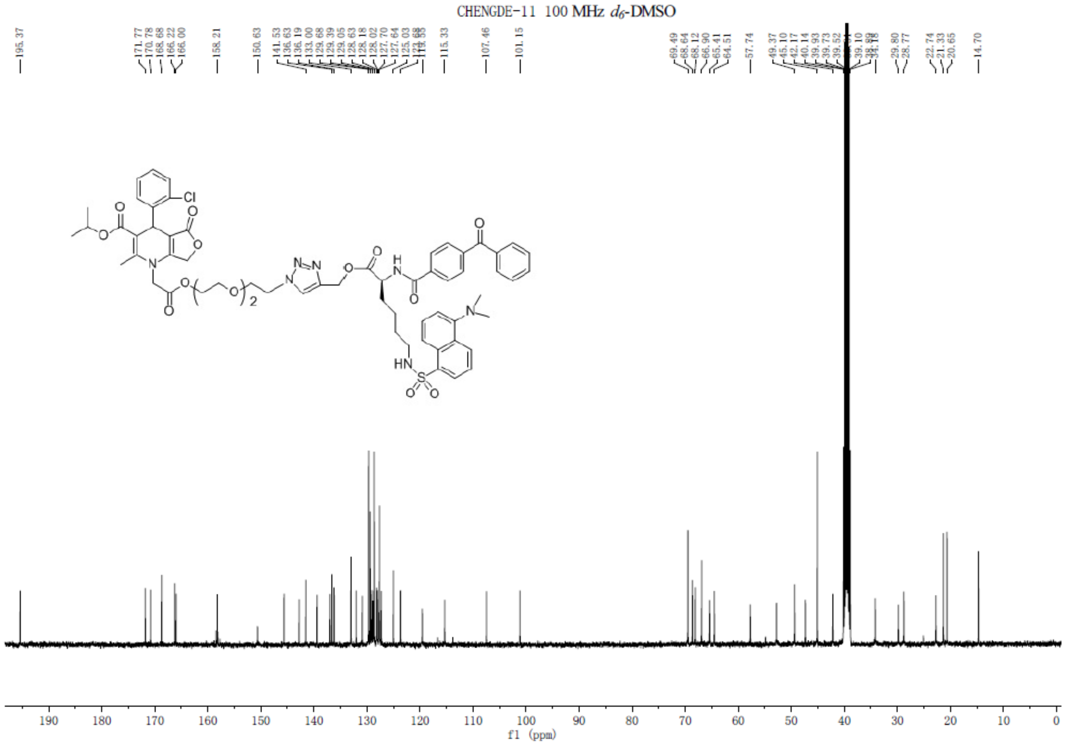

^{1}H NMR spectra of compound **3d**

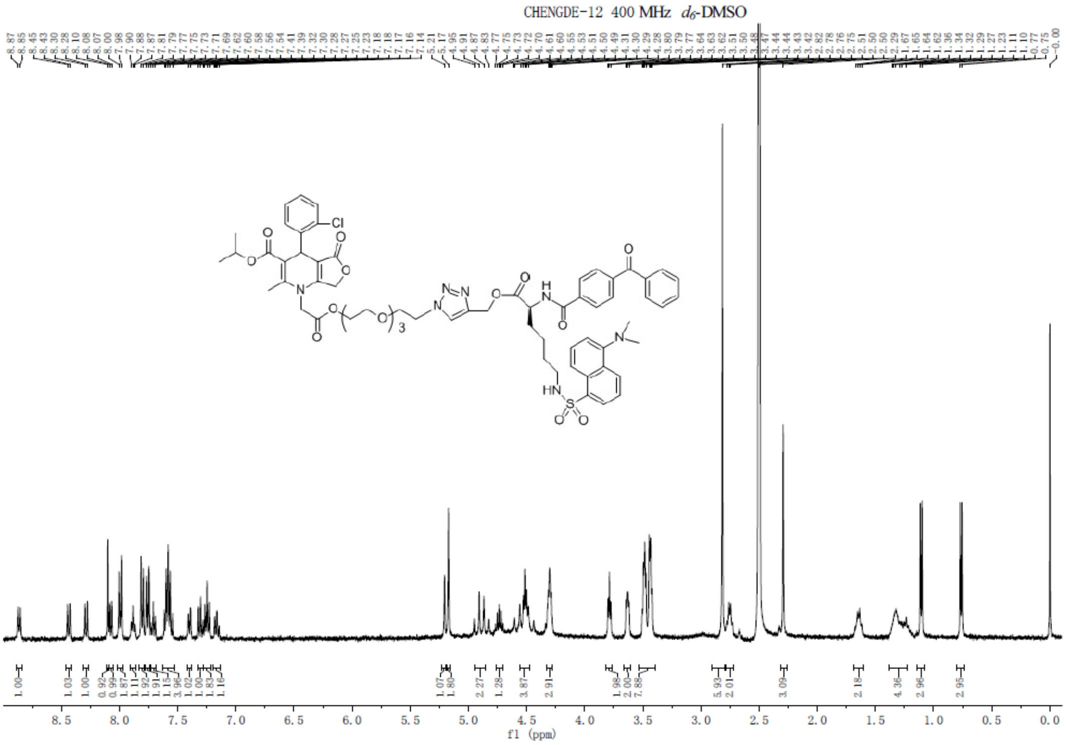

^{13}C NMR spectra of compound **3d**

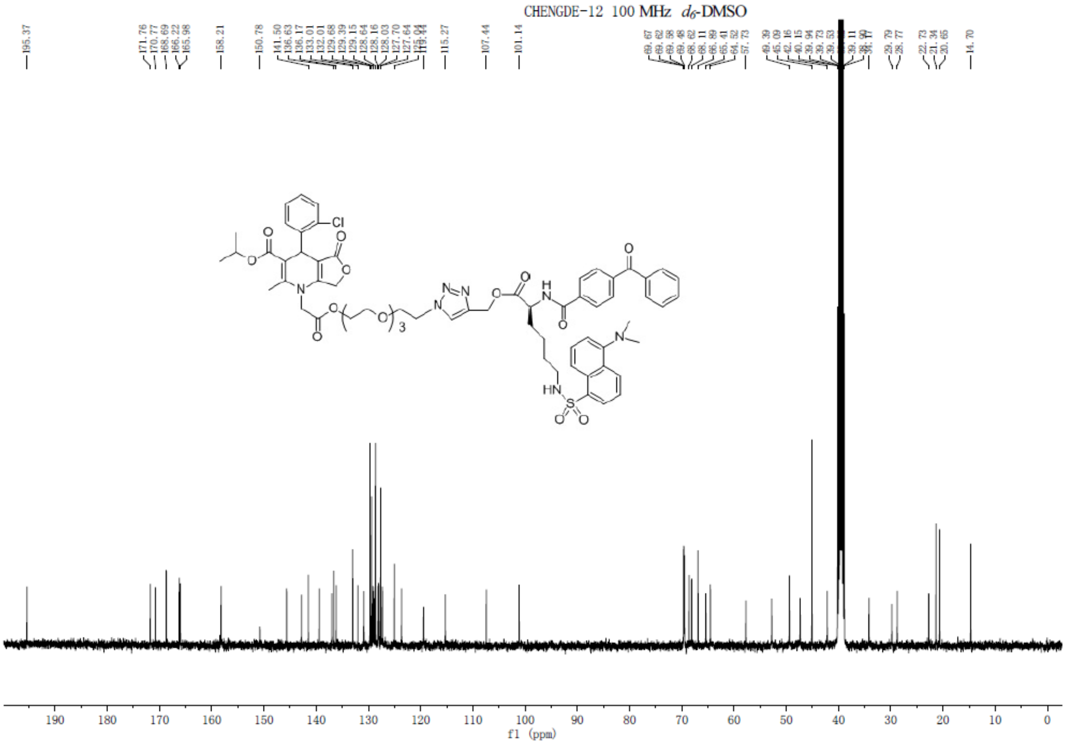

^{1}H NMR spectra of compound **4a**

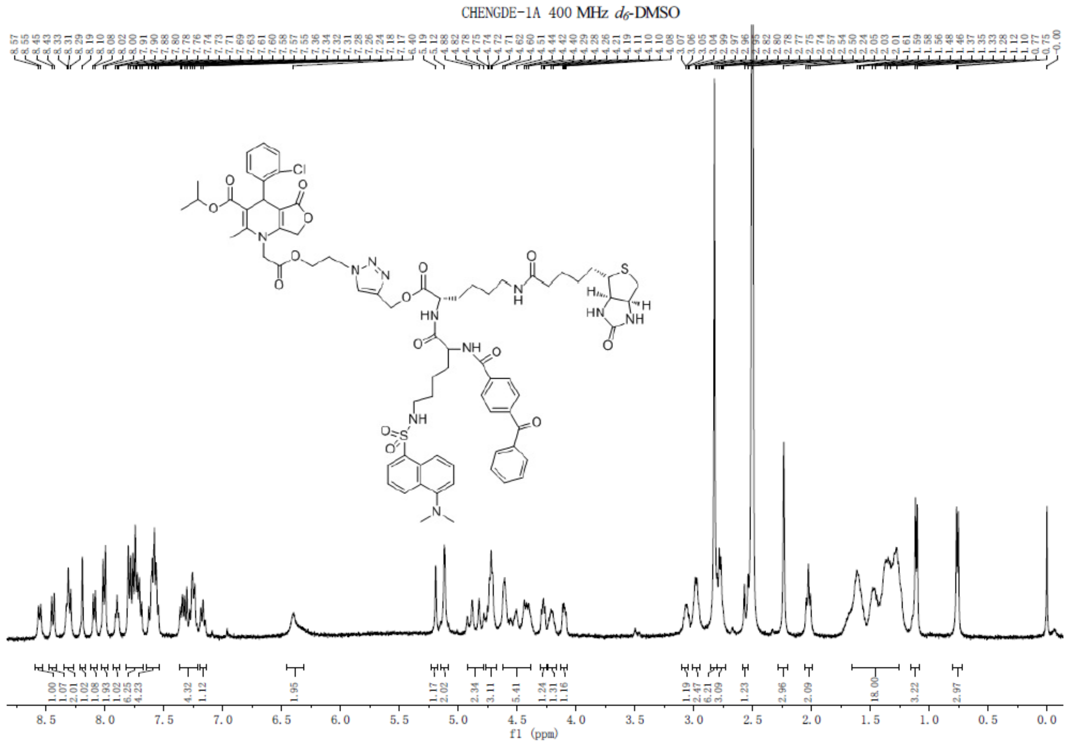

^{13}C NMR spectra of compound **4a**

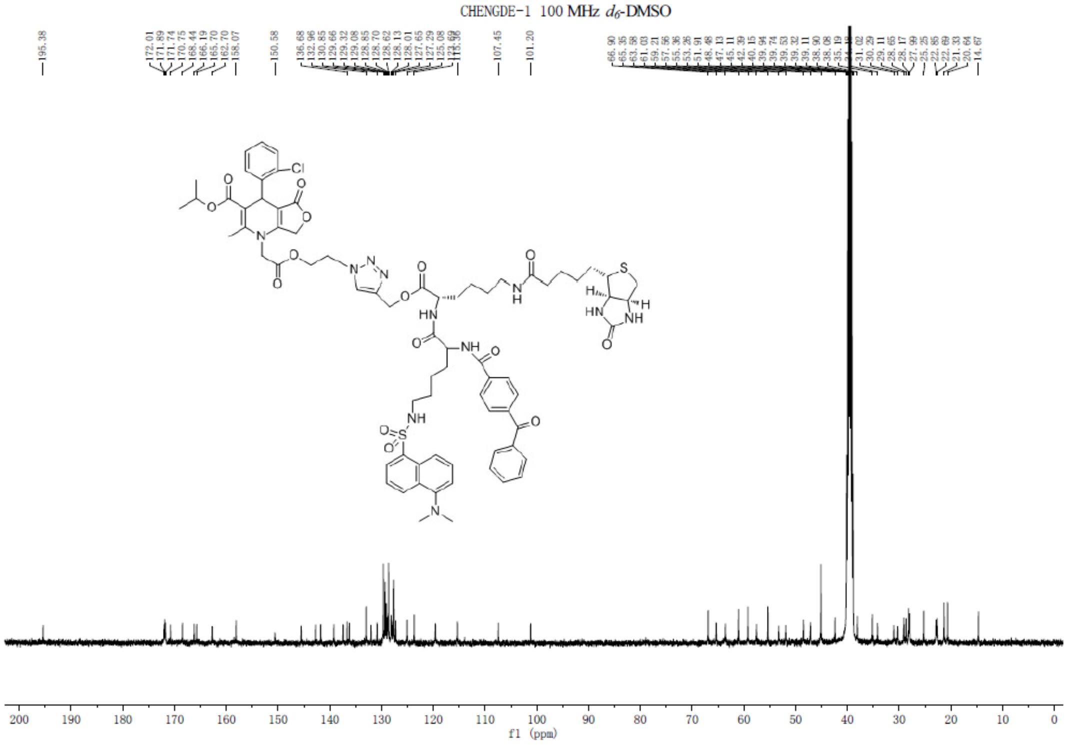

^{1}H NMR spectra of compound **4b**

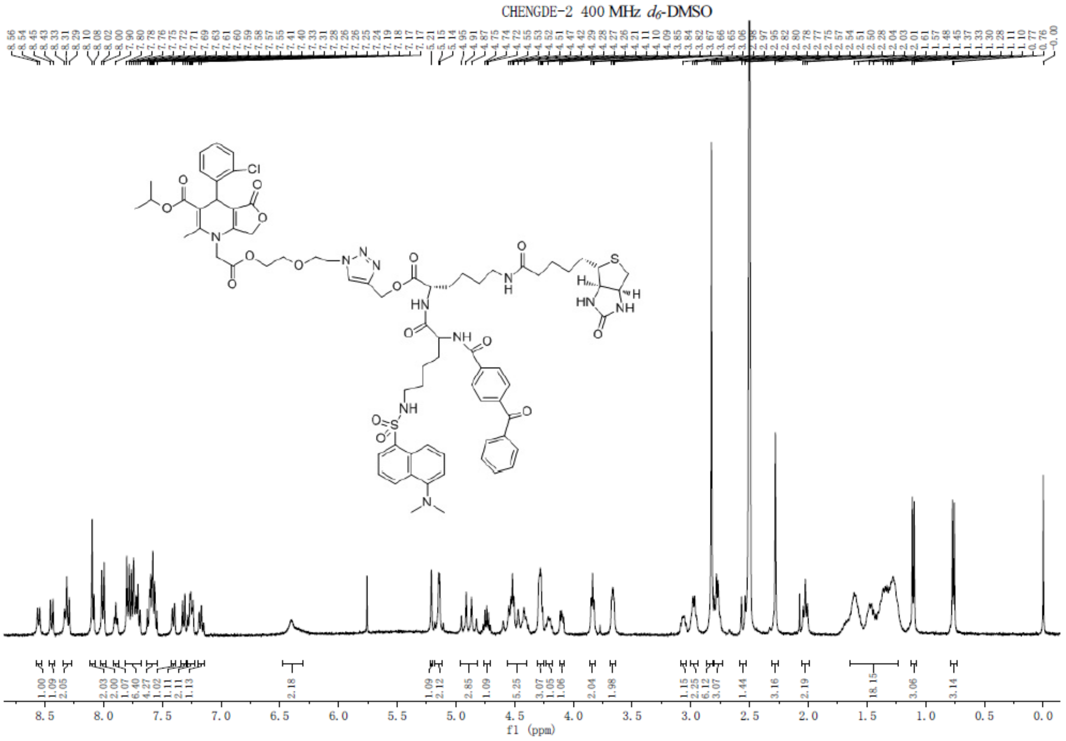

^{13}C NMR spectra of compound **4b**

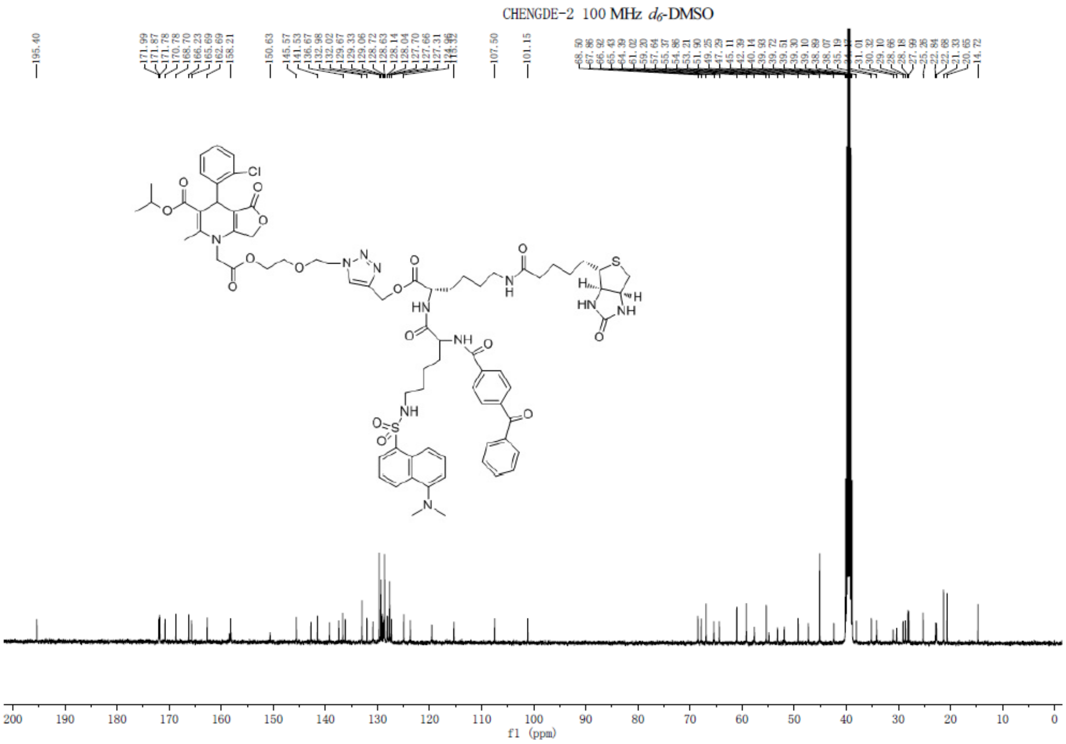

^{1}H NMR spectra of compound **4c**

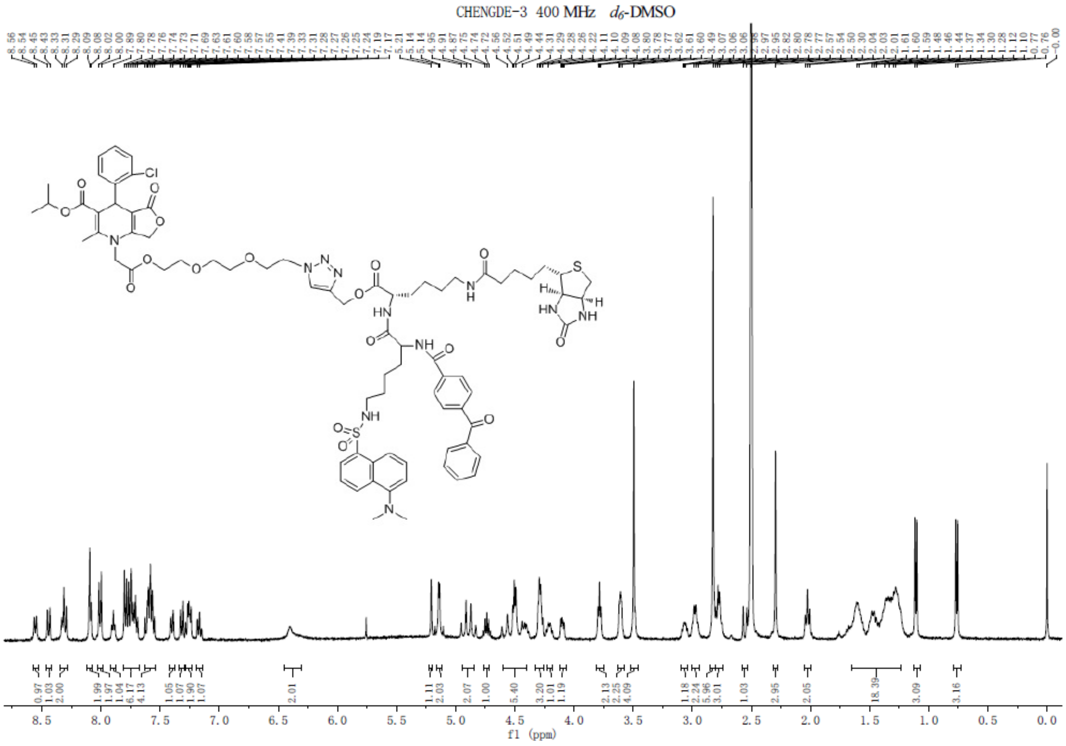

^{13}C NMR spectra of compound **4c**

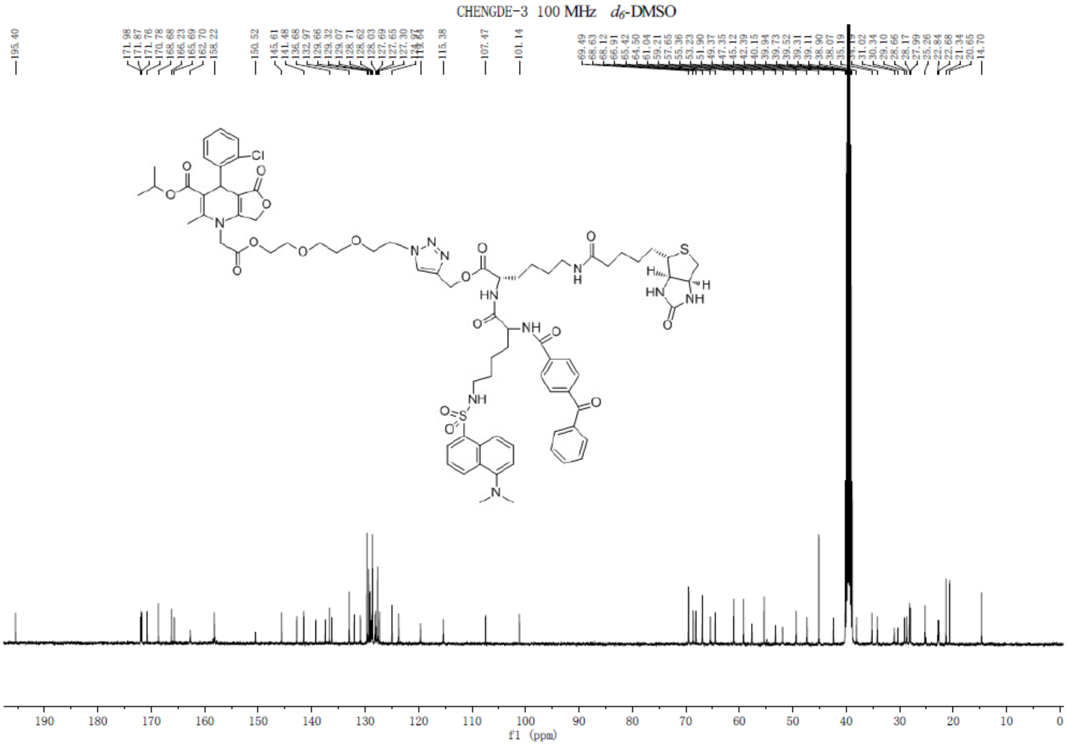

^{1}H NMR spectra of compound **4d**

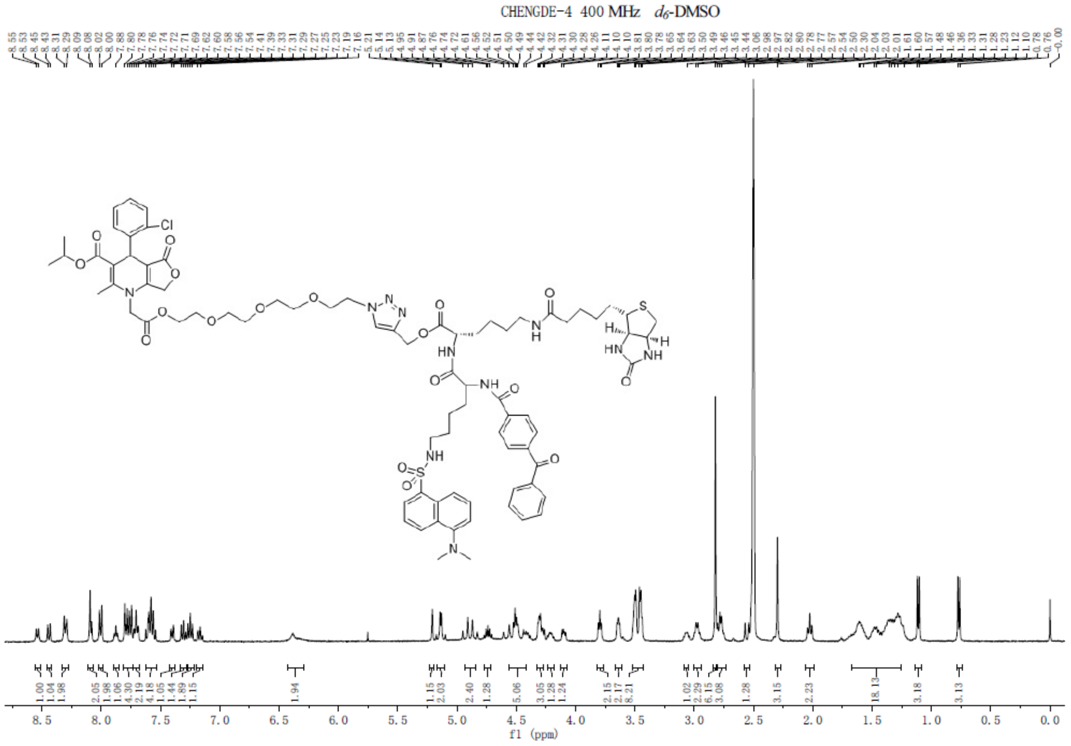

^{13}C NMR spectra of compound **4d**

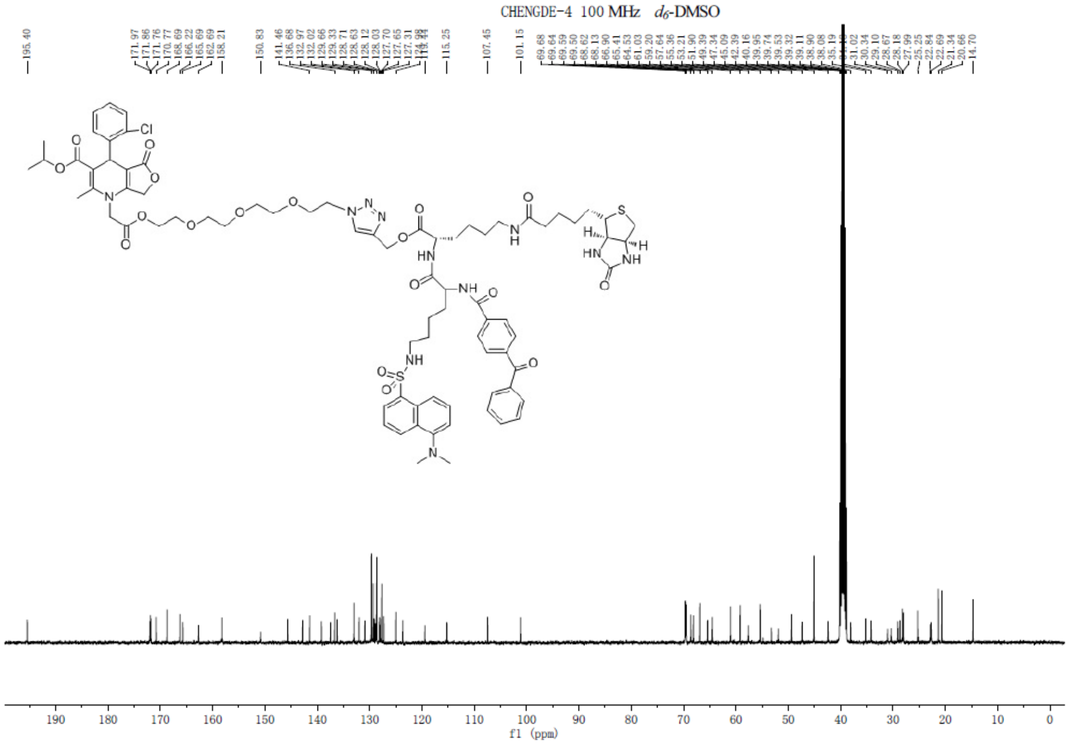

^{1}H NMR spectra of compound **31a**

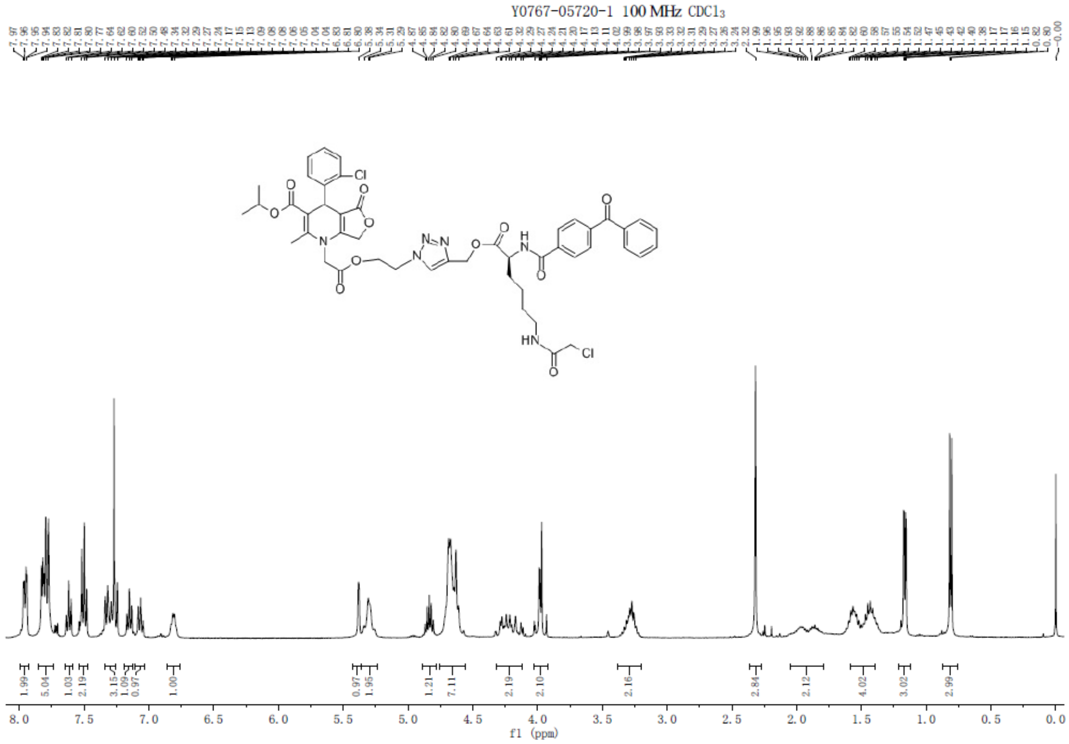

^{13}C NMR spectra of compound **31a**

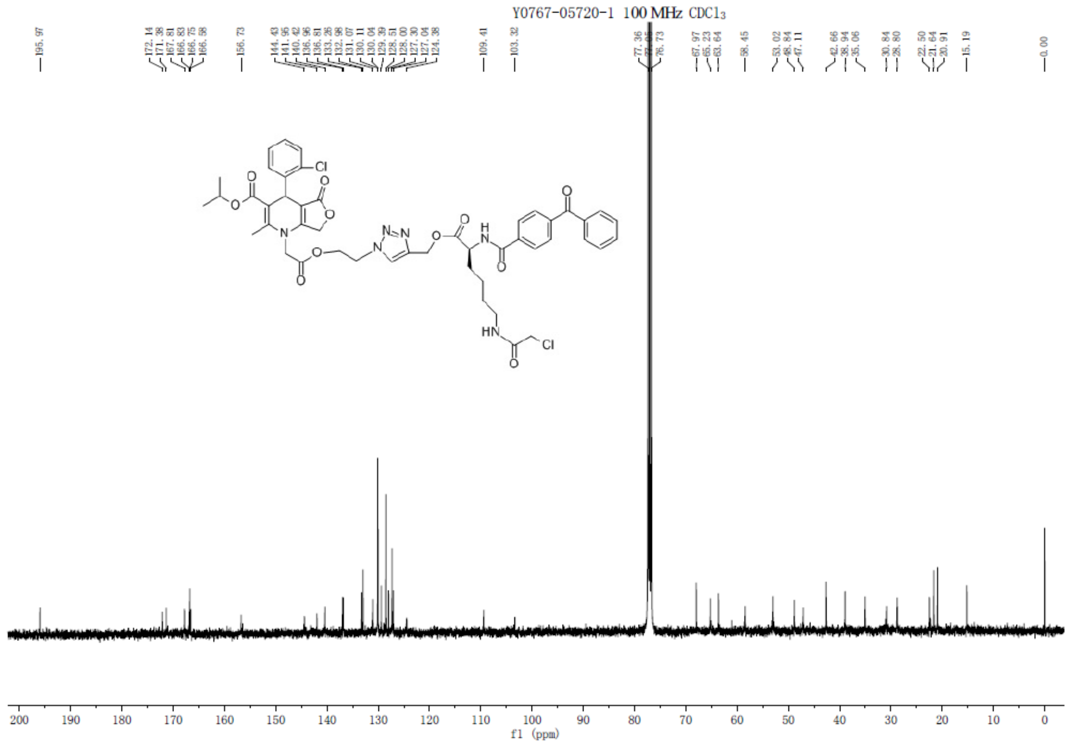

^{1}H NMR spectra of compound **31b**

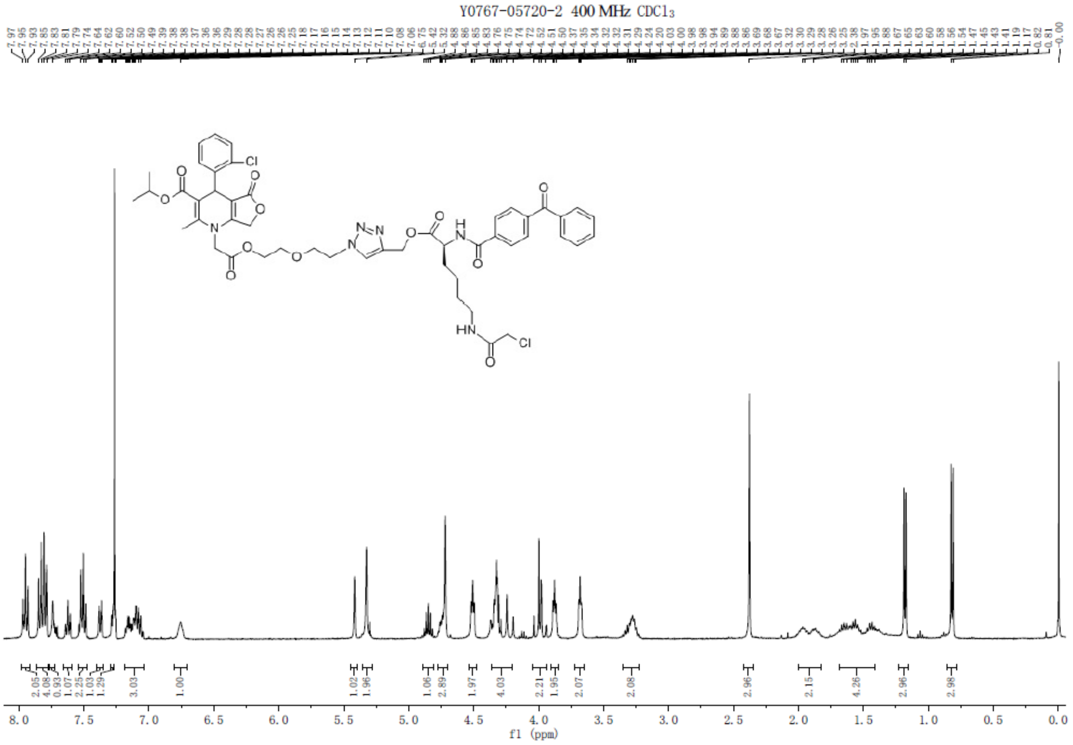

^{13}C NMR spectra of compound **31b**

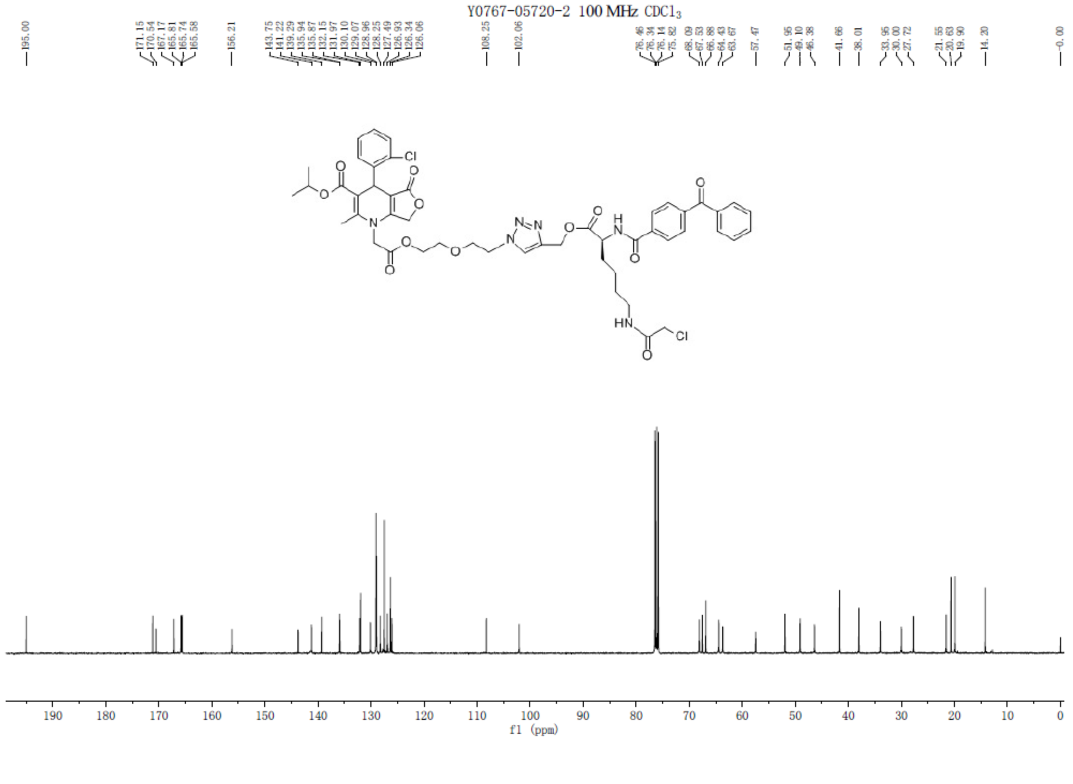

^{1}H NMR spectra of compound **31c**

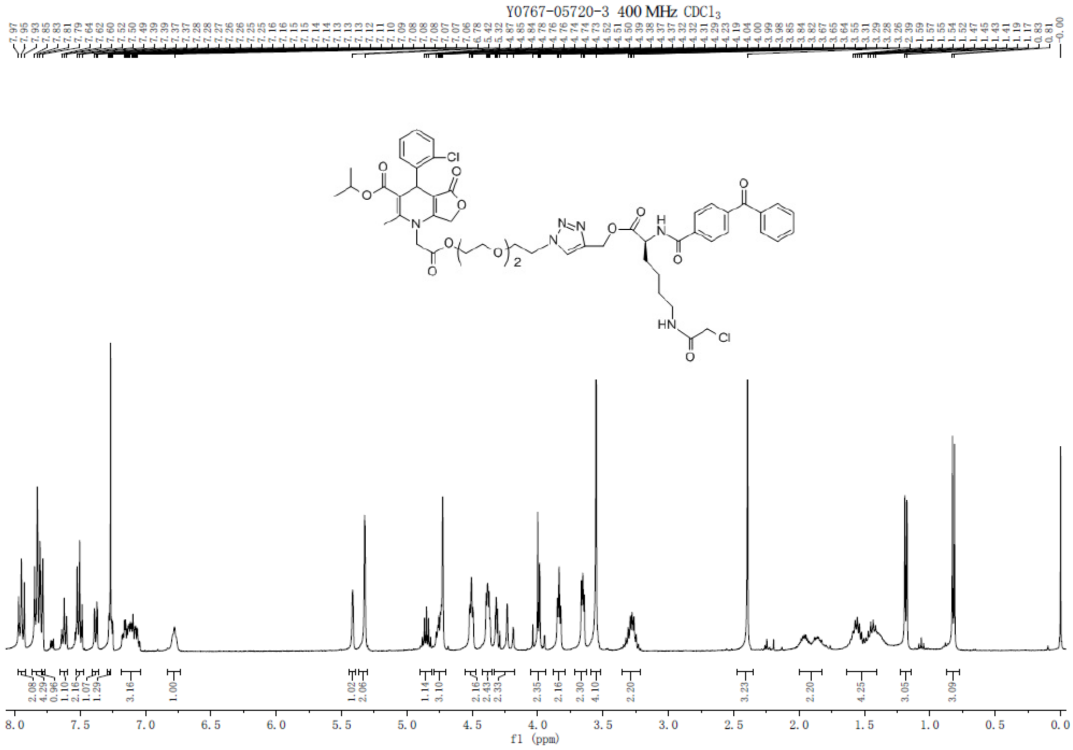

^{13}C NMR spectra of compound **31c**

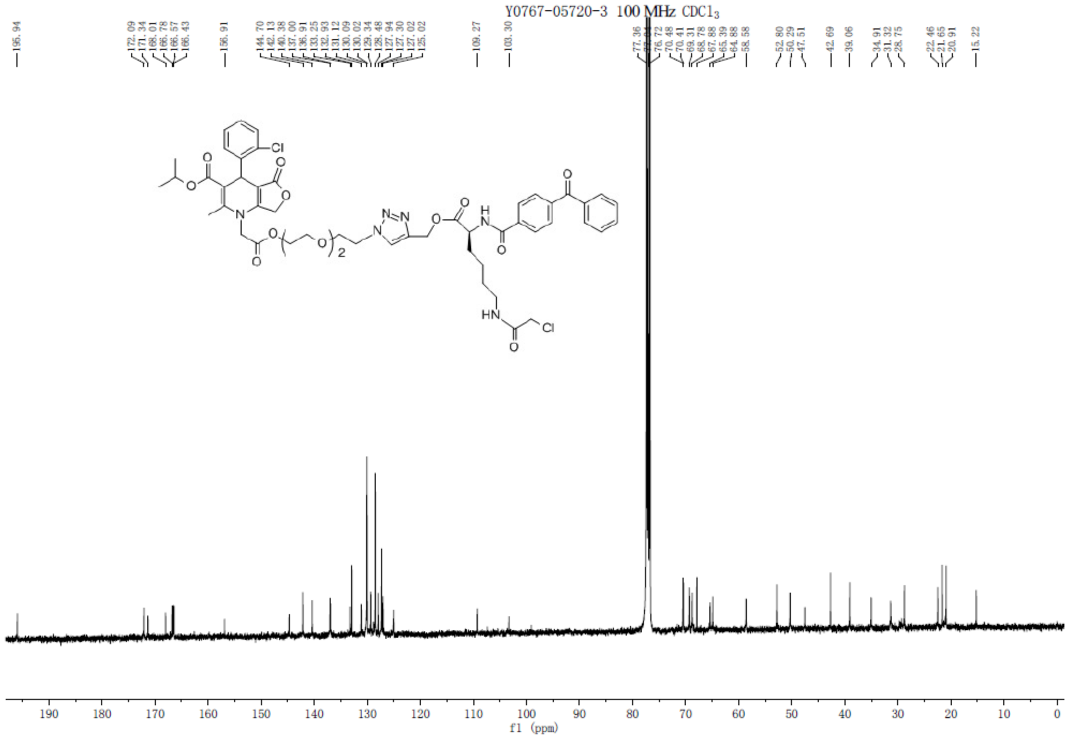

^{1}H NMR spectra of compound **31d**

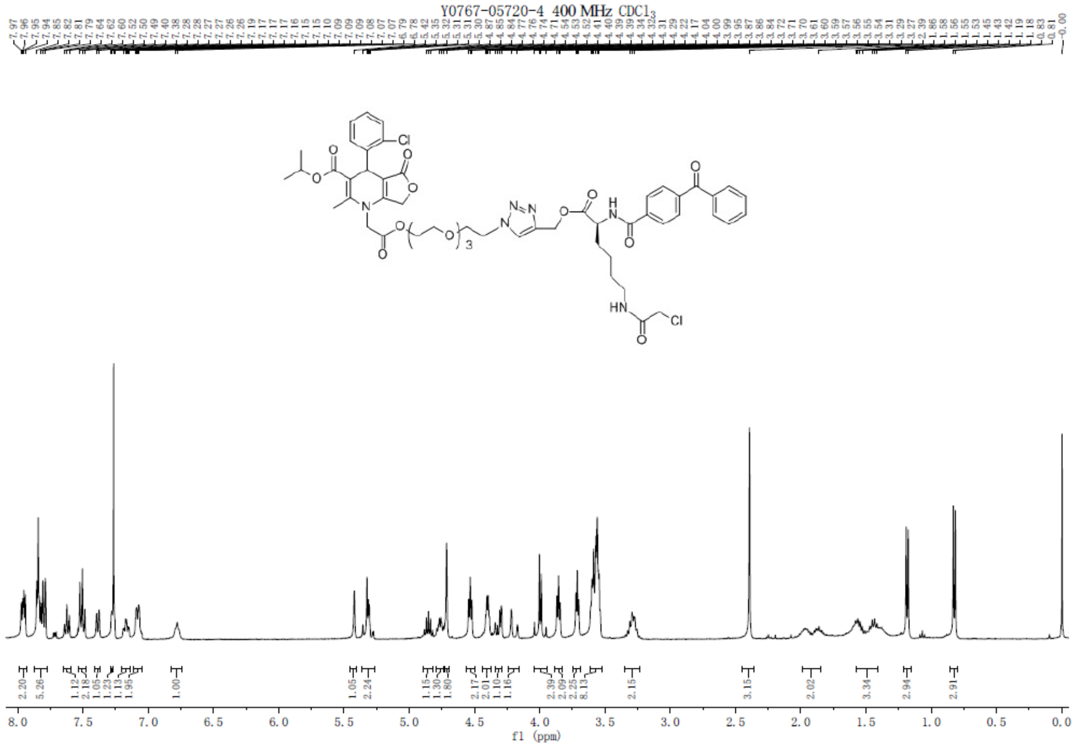

^{13}C NMR spectra of compound **31d**

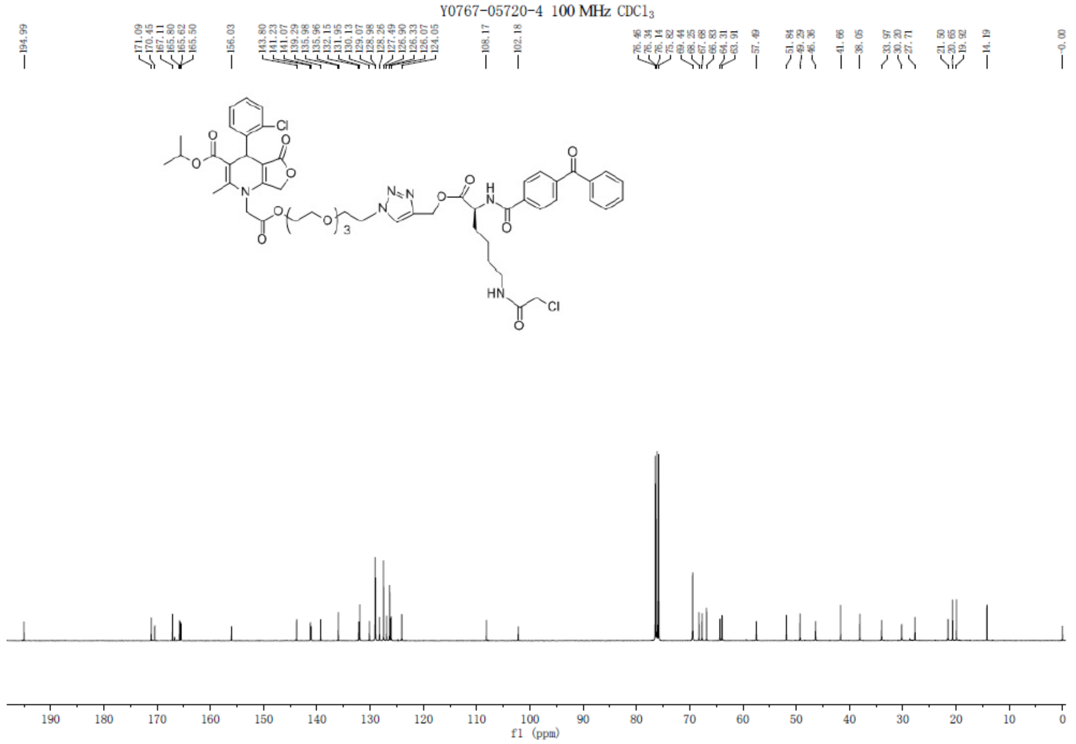

^{1}H NMR spectra of compound **32**

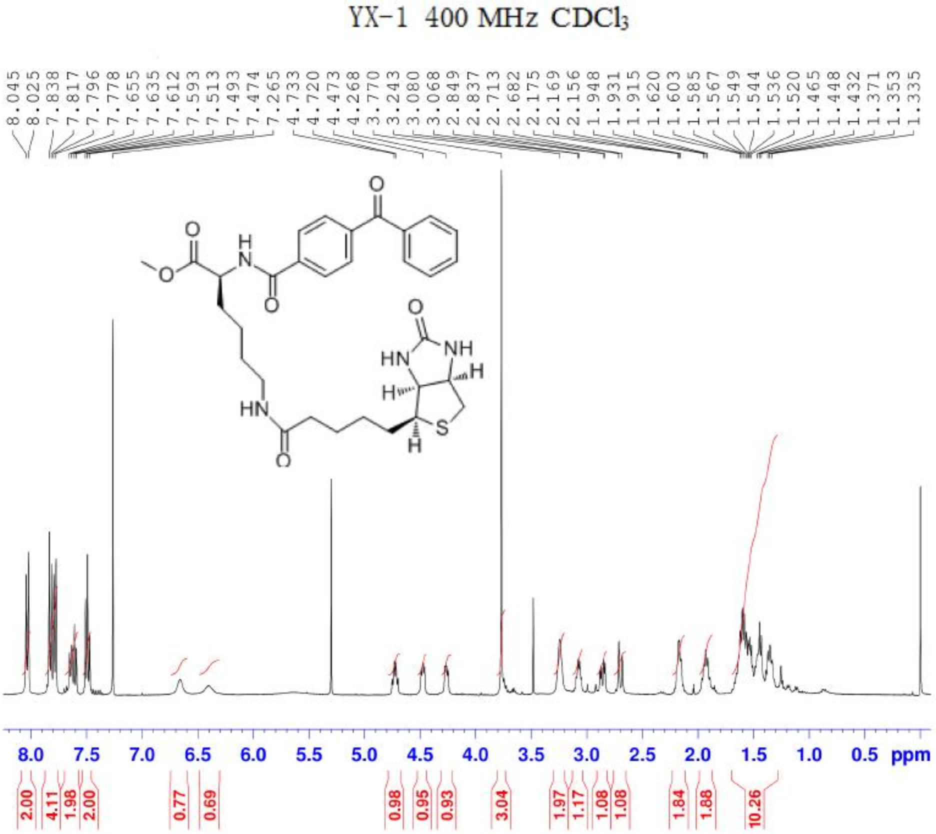

^{13}C NMR spectra of compound **32**

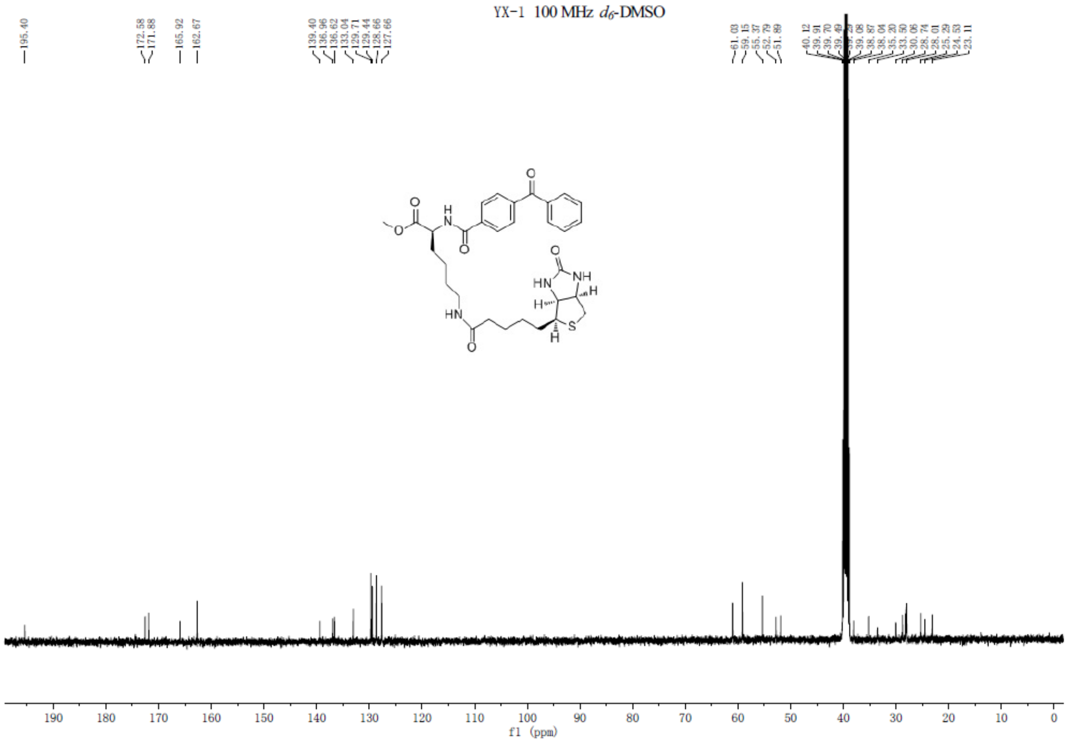

^{1}H NMR spectra of compound **5a**

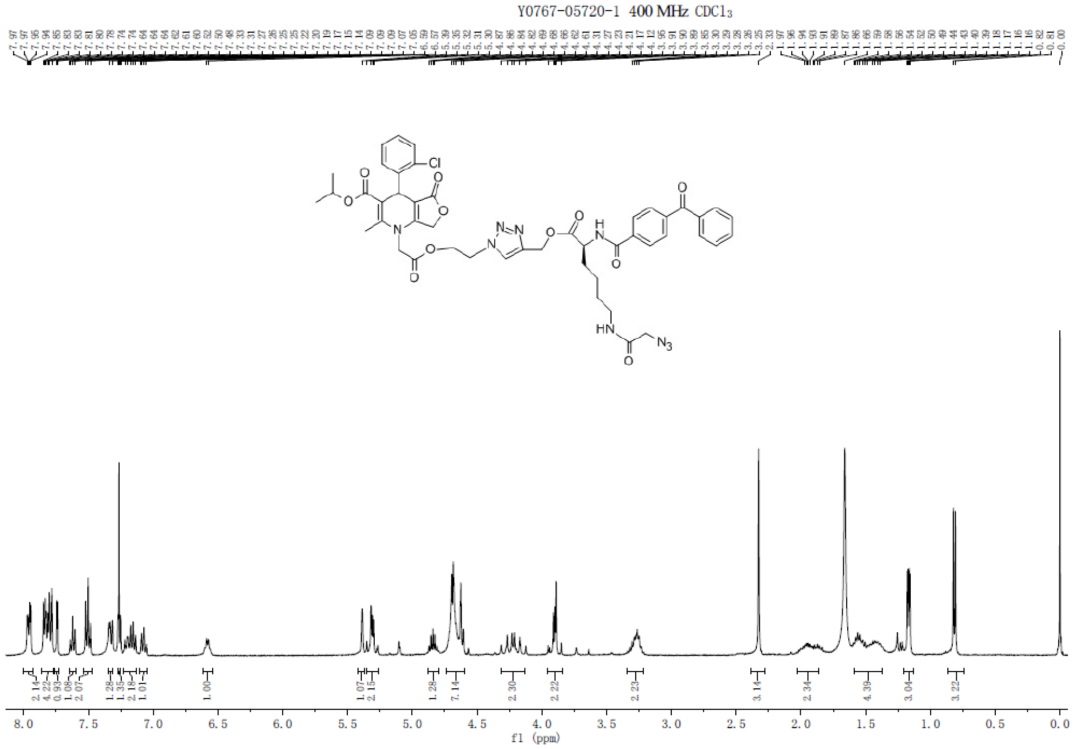

^{13}C NMR spectra of compound **5a**

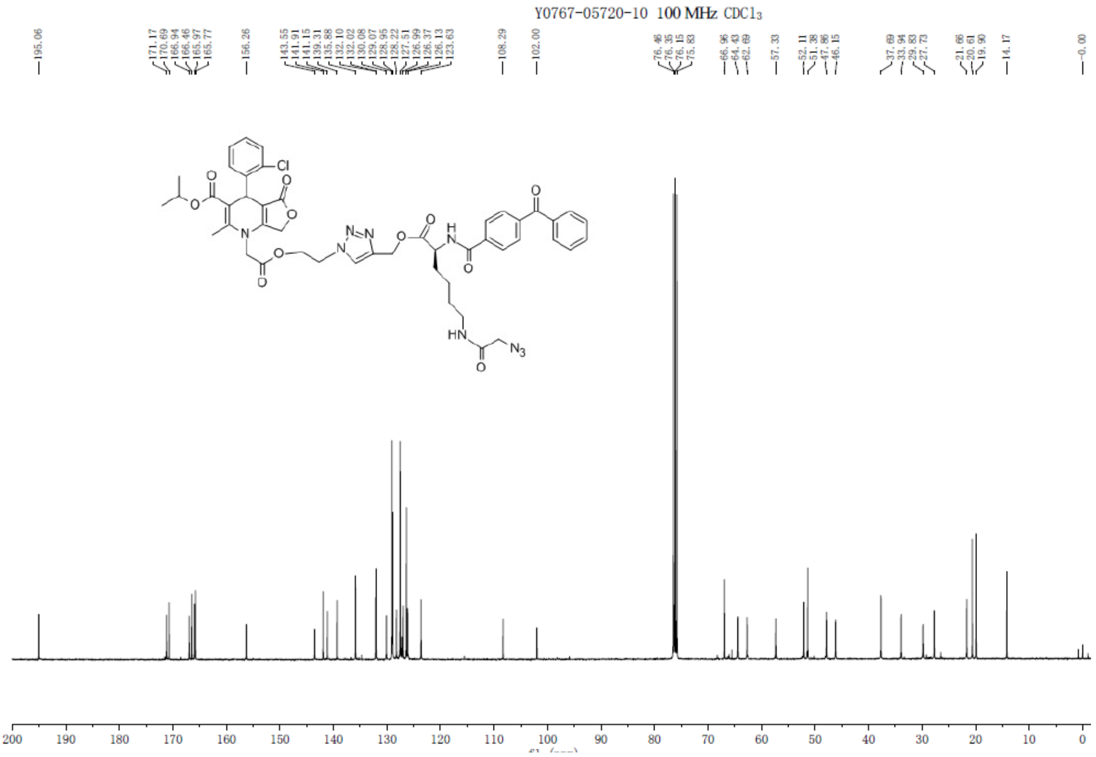

^{1}H NMR spectra of compound **5b**

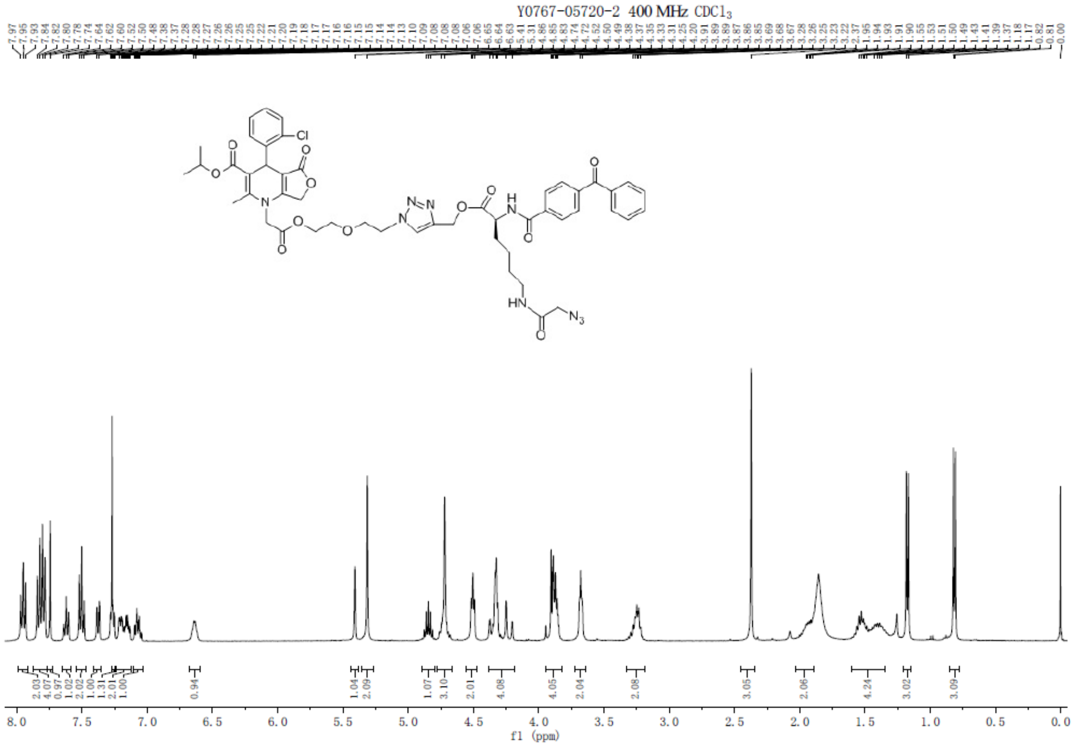

^{13}C NMR spectra of compound **5b**

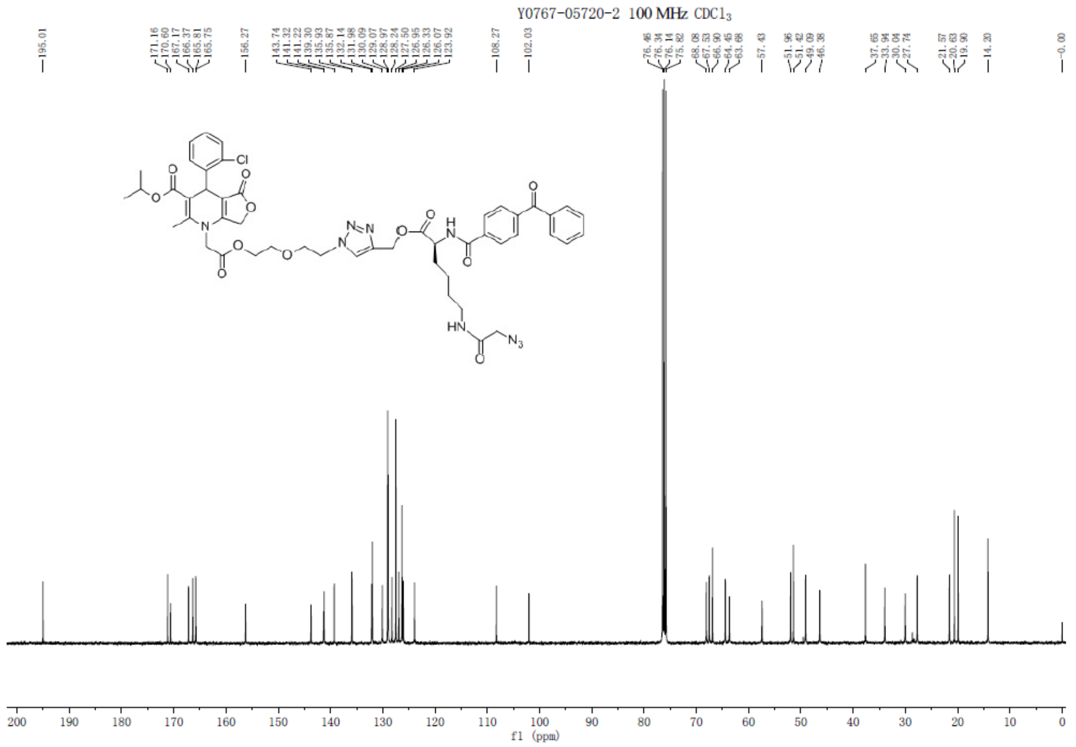

^{1}H NMR spectra of compound **5c**

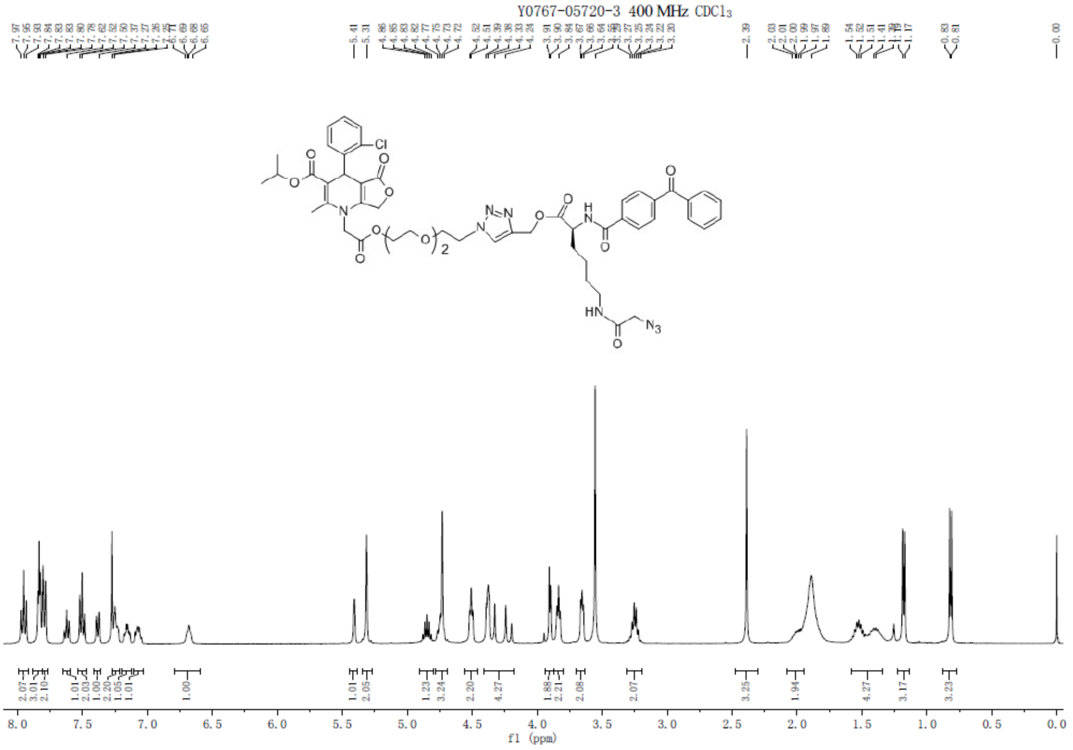

^{13}C NMR spectra of compound **5c**

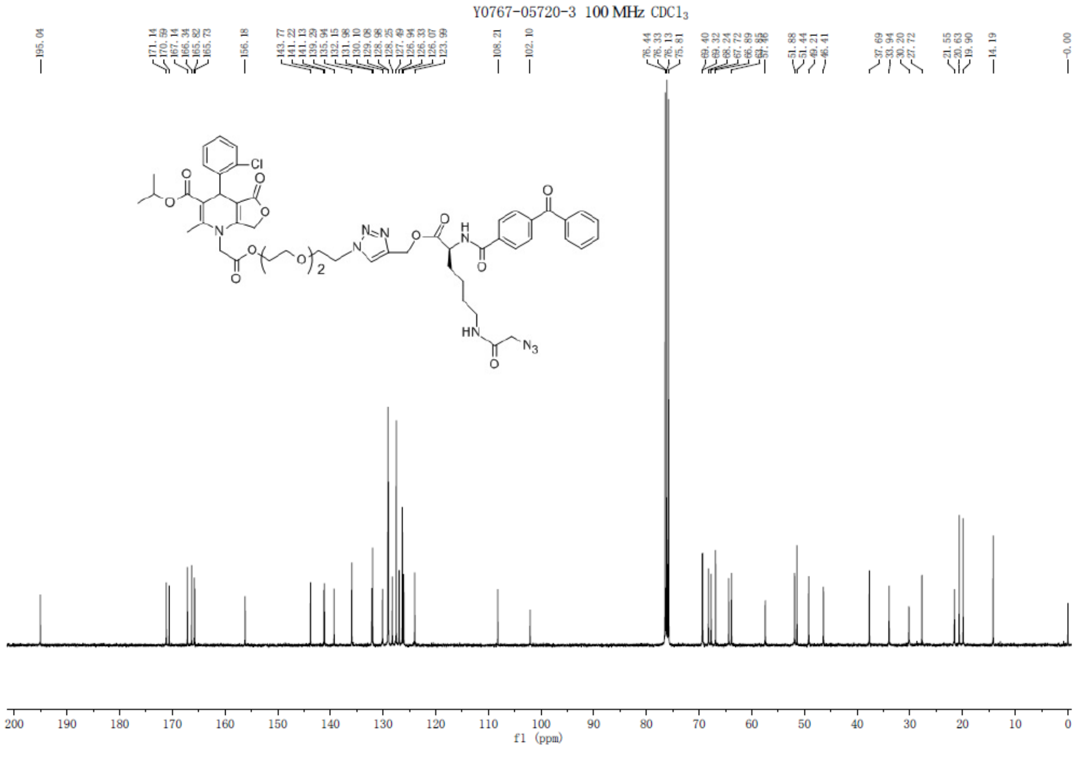

^{1}H NMR spectra of compound **5d**

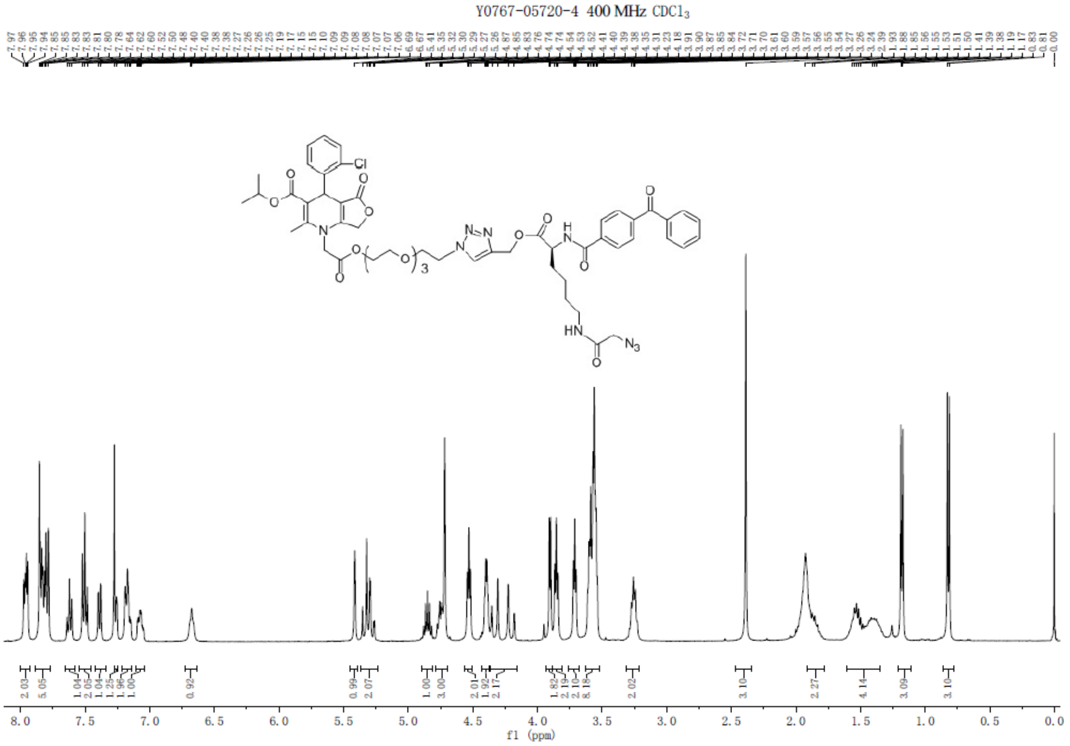

^{13}C NMR spectra of compound **5d**

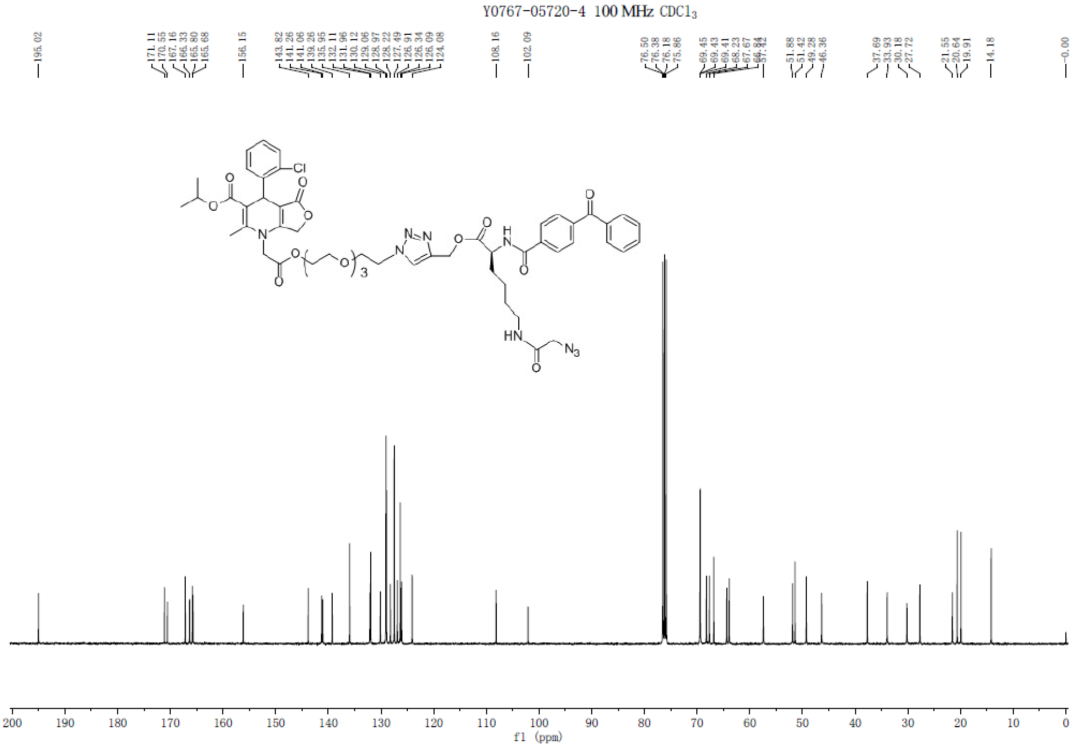

^{1}H NMR spectra of compound **33**

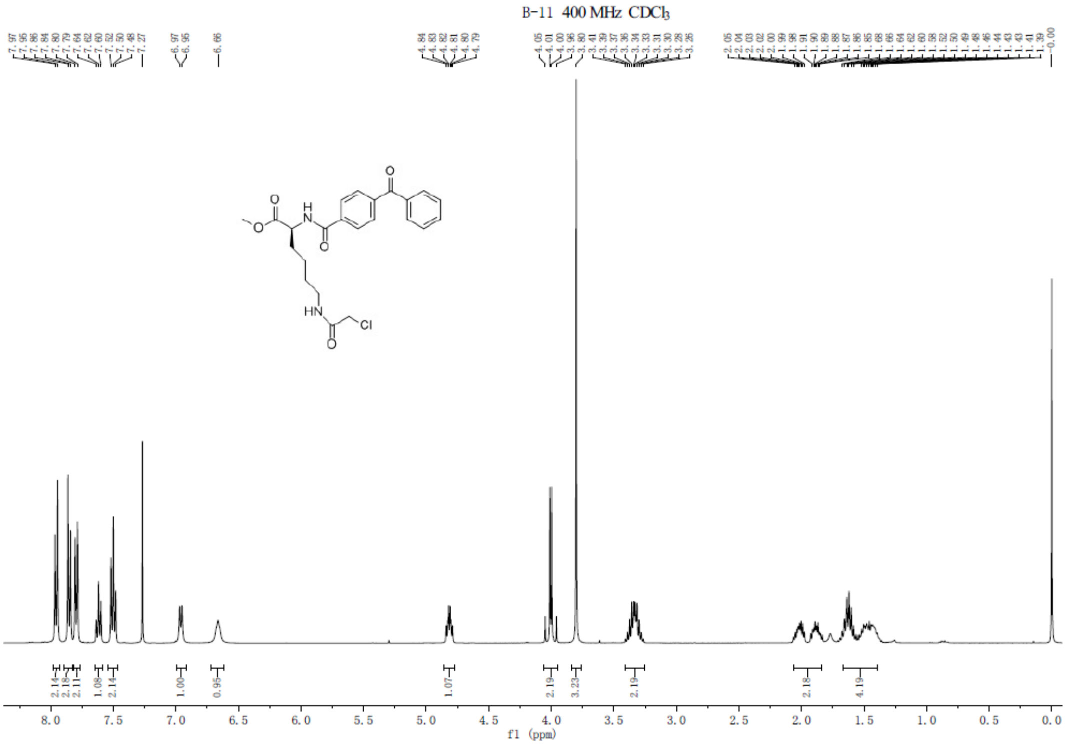

^{13}C NMR spectra of compound **33**

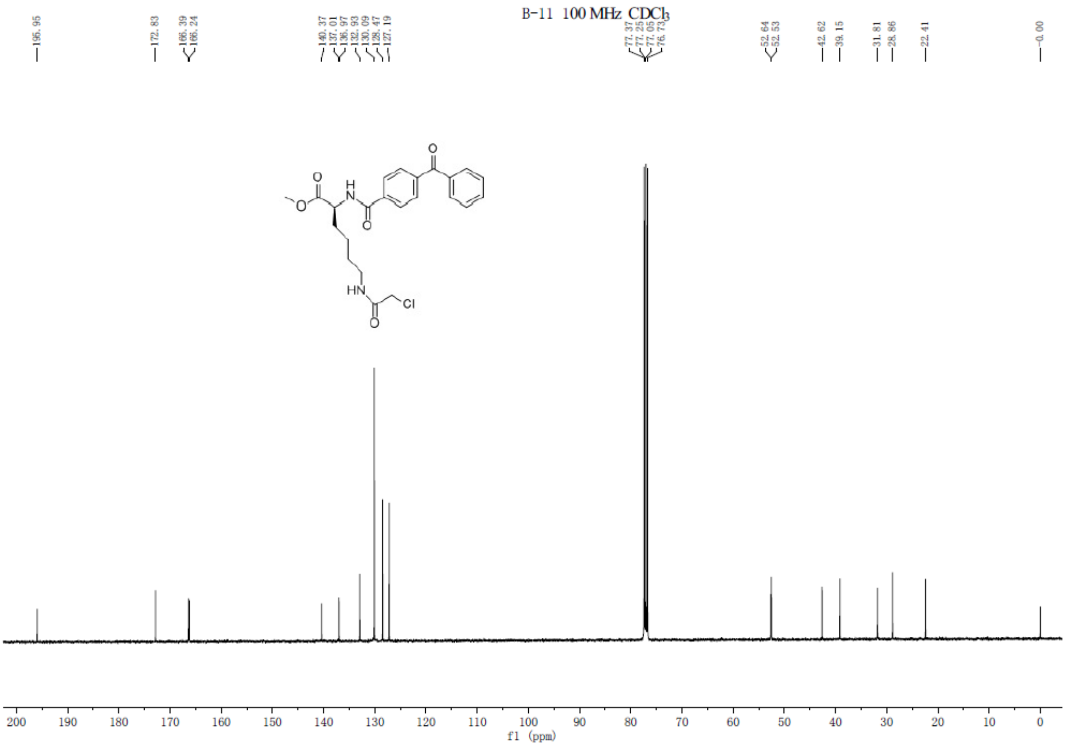

^{1}H NMR spectra of compound **34**

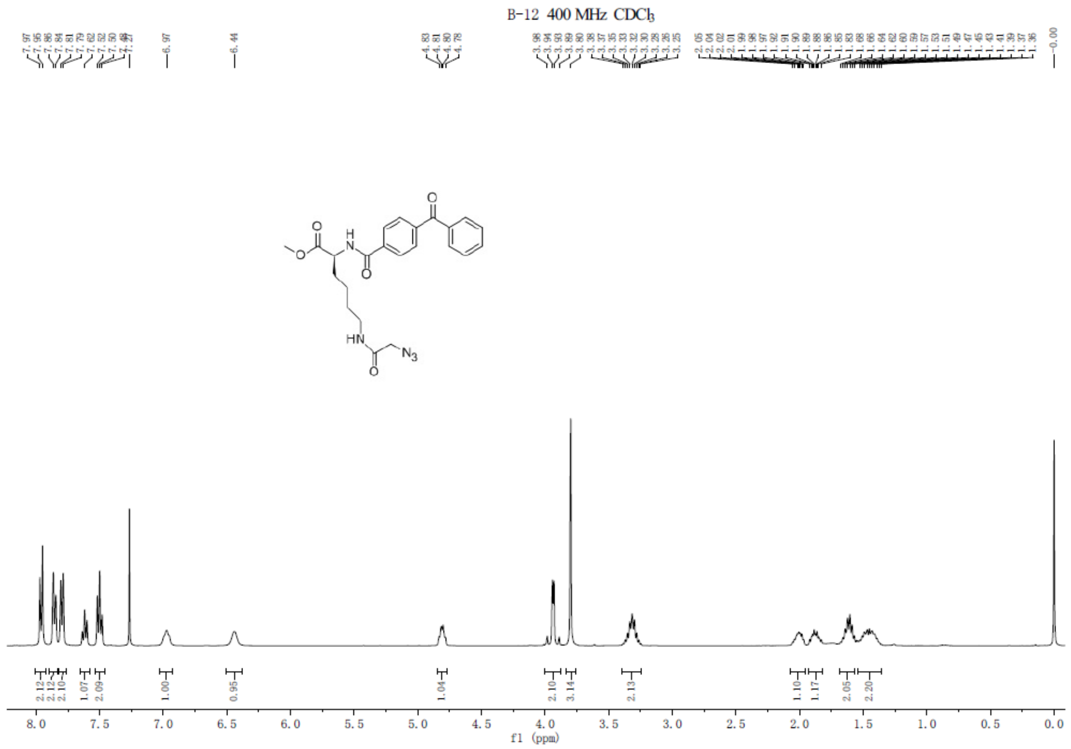

^{13}C NMR spectra of compound **34**

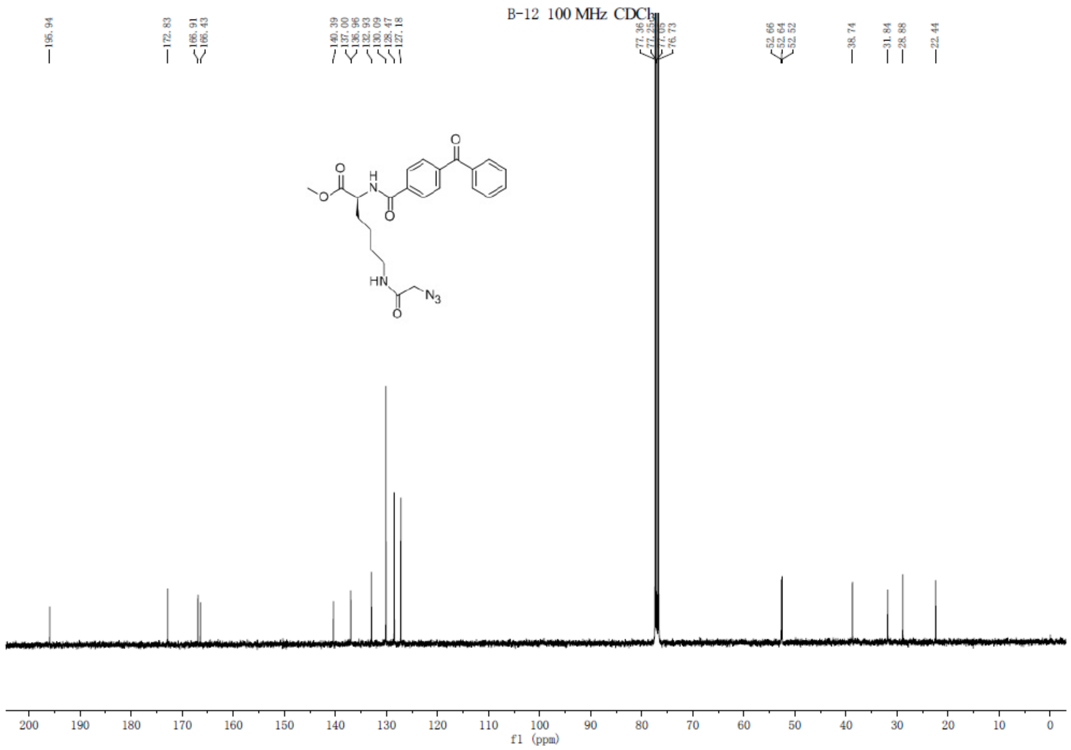

^{1}H NMR spectra of compound **36**

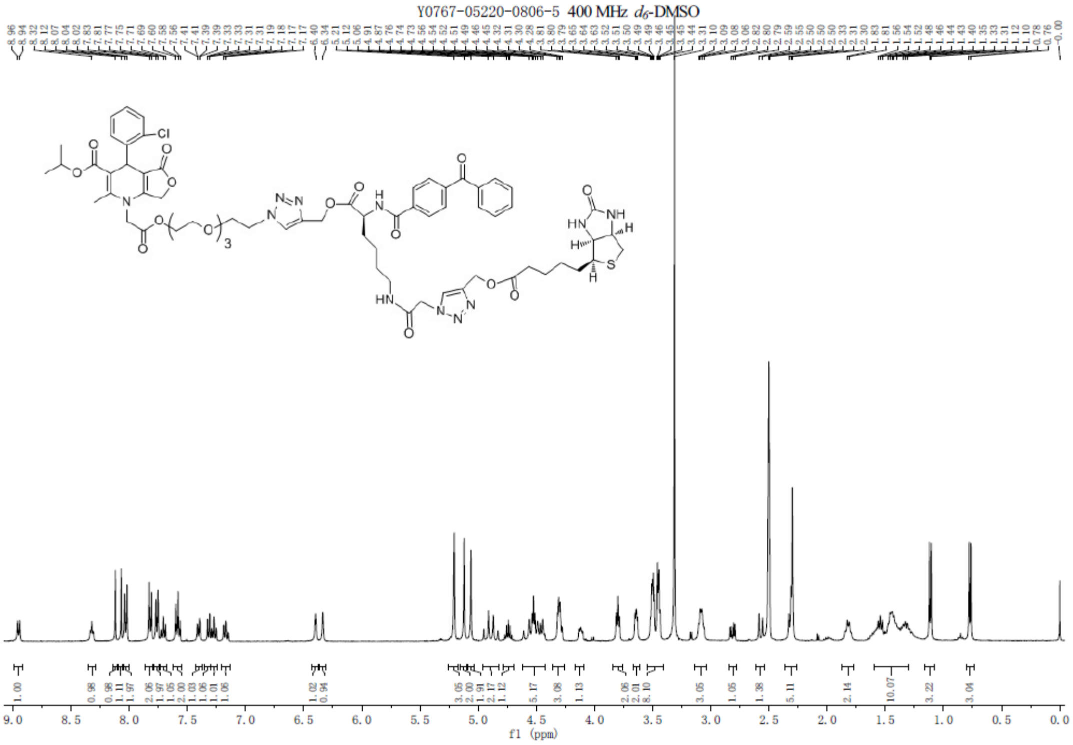

^{13}C NMR spectra of compound **36**

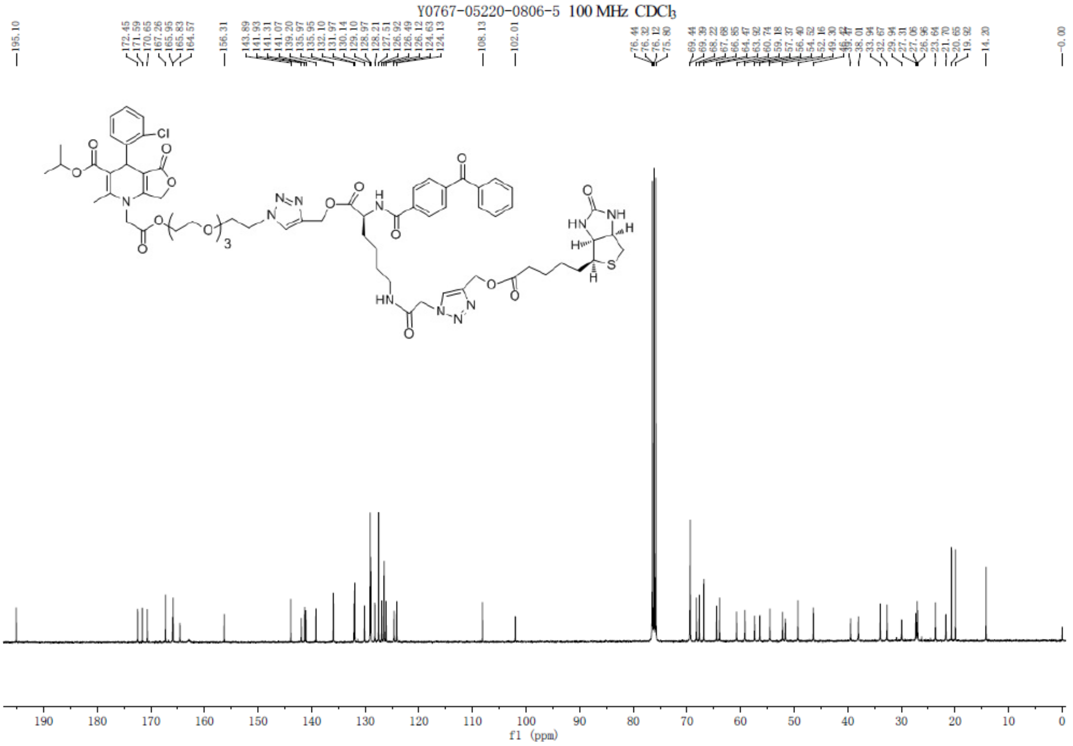

Figure 1. Image of the SDS-PAGE gel stained with Coomassie R-250.

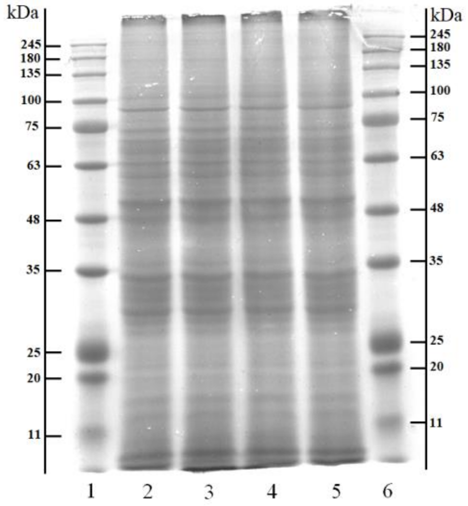

Photoaffinity labeling of the soluble proteomes prepared from HepG2 cells followed SDS-PAGE electrophoresis separated and stained with Coomassie blue (CB) as described in the Experimental procedures. Samples were prepared by incubating 2.0 mg/mL proteomes at different conditions: (Lane 1 and 6) Marker; **(**Lane 2) With 10 μM probe **2d** and exposed to UV light for 30 min; (Lane 3) With 10 μM probe **2d** and **BAY R3401** then exposed to UV light for 30 min; (Lane 4) With 10 μM control compound **32** and exposed to UV light for 30 min; (Lane 5) With 0 μM probe **2d** and exposed to UV light for 30 min.

References

Yu, Y.; Qin, A.; Feng, C.; Lu, P.; Ng, K.M.; Luo, K.Q.; Tang, B.Z. An amine-reactive tetraphenylethylene derivative for protein detection in SDS-PAGE. *Analyst.* **137**, 5592-5596. (2012).
